# Synthesis, Structural Characterization, and DFT Investigations of [M_*x*_M′_5–*x*_Fe_4_(CO)_16_]^3–^ (M, M′ = Cu, Ag, Au; M ≠ M′) 2-D Molecular Alloy Clusters

**DOI:** 10.1021/acs.inorgchem.0c02443

**Published:** 2020-10-20

**Authors:** Beatrice Berti, Marco Bortoluzzi, Cristiana Cesari, Cristina Femoni, Maria Carmela Iapalucci, Leonardo Soleri, Stefano Zacchini

**Affiliations:** † Dipartimento di Chimica Industriale “Toso Montanari”, University of Bologna, Viale Risorgimento 4, I-40136 Bologna, Italy; ‡ Dipartimento di Scienze Molecolari e Nanosistemi, Ca’ Foscari University of Venice, Via Torino 155, 30175 Mestre (Ve), Italy

## Abstract

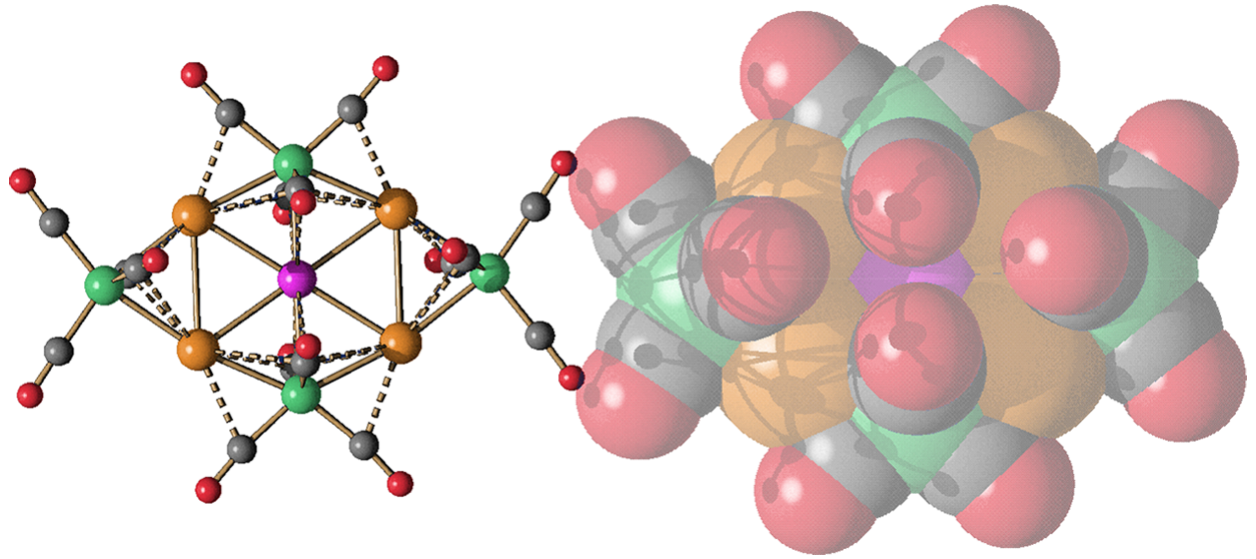

Miscellaneous 2-D molecular alloy clusters of the type [M_*x*_M′_5–*x*_Fe_4_(CO)_16_]^3–^ (M, M′ = Cu, Ag, Au; M ≠ M′) have been prepared through the reactions of [Cu_3_Fe_3_(CO)_12_]^3–^, [Ag_4_Fe_4_(CO)_16_]^4–^ or [M_5_Fe_4_(CO)_16_]^3–^ (M = Cu, Ag) with M′(I) salts (M′ = Cu, Ag, Au). Their formation involves a combination of oxidation, condensation, and substitution reactions. The total structures of several [M_*x*_M′_5–*x*_Fe_4_(CO)_16_]^3–^ clusters with different compositions have been determined by means of single crystal X-ray diffraction (SC-XRD) and their nature in solution elucidated by electron spray ionization mass spectrometry (ESI-MS) and IR and UV–visible spectroscopy. Substitutional and compositional disorder is present in the solid state structures, and ESI-MS analyses point out that mixtures of isostructural clusters differing by a few M/M′ coinage metals are present. SC-XRD studies indicate some site preferences of the coinage metals within the metal cores of these clusters, with Au preferentially in corner sites and Cu in the central site. DFT studies give theoretical support to the experimental structural evidence. The site preference is mainly dictated by the strength of the Fe–M bonds that decreases in the order Fe–Au > Fe–Ag > Fe–Cu, and the preferred structure is the one that maximizes the number of stronger Fe–M interactions. Overall, the molecular nature of these clusters allows their structures to be fully revealed with atomic precision, resulting in the elucidation of the bonding parameters that determine the distribution of the different metals within their metal cores.

## Abstract 2

The distribution of coinage metals in 2-D molecular alloy clusters has been investigated by IR, UV−visible, ESI-MS, X-ray crystallography, and DFT methods, revealing substitutional and compositional disorder as well as some site preference.

## 1 Introduction

In the recent years, alloying has attracted considerable interest in the field of molecular metal clusters and nanoclusters from both a fundamental and applicative point of view.^1−6^ Indeed, mixing different metals with atomic control may lead to nanomaterials with new physical or chemical properties.^7−10^ Different methods may be used for the synthesis of alloy clusters, including direct reduction, redox condensation, and metal exchange.^11,12^ Redox condensation has been widely exploited in the field of molecular metal carbonyl clusters.^13−18^ Conversely, direct metal reduction and metal exchange have been mainly applied to Au nanoclusters. The former method often does not allow the structure of the resulting alloy nanoclusters to be controlled. Better results are obtained by selective metal exchange in preformed atomically precise Au nanoclusters.^1,2,9,11,12^

Thiolate protected alloy nanoclusters and, generally speaking, Au-based alloy nanoclusters have been largely investigated in recent years, as part of the great interest for atomically precise and ultrasmall Au nanoparticles.^19−27^ Within this framework, we reported some years ago a few examples of molecular Au nanoclusters protected by Fe-carbonyl fragments, that is, [Au_21_{Fe(CO)_4_}_10_]^5–^, [Au_22_{Fe(CO)_4_}_12_]^6–^, [Au_28_{Fe(CO)_3_}_4_{Fe(CO)_4_}_10_]^8–^, and [Au_34_{Fe(CO)_3_}_6_{Fe(CO)_4_}_8_]^10–^.^28^ This may be viewed as an organometallic approach to metal nanoparticles, that includes also the [Ni_32_Au_6_(CO)_44_]^6–^ and [Ni_12_Au_6_(CO)_24_]^2–^ clusters containing a Au_6_ core stabilized by Ni–CO moieties^29,30^ and Pd clusters protected by Co-carbido-carbonyl fragments, that is, [H_4–*n*_Co_20_Pd_16_C_4_(CO)_48_]^*n*−^ (*n* = 2–4), [H_3–*n*_Co_15_Pd_9_C_3_(CO)_38_]^*n*−^ (*n* = 0–3), and [Co_13_Pd_3_C_3_(CO)_29_]^−^.^31^ Thus, planar organometallic species such as [M_3_Fe_3_(CO)_12_]^3–^ (M = Cu, Ag, Au),^32,33^ [M_4_Fe_4_(CO)_16_]^4–^ (M = Ag, Au),^34,35^ and [M_5_Fe_4_(CO)_16_]^3–^ (M = Cu, Ag, Au)^28,32,34^ may be viewed as 2-D molecular clusters, consisting of triangular M_3_, square M_4_, or centered rectangular M_5_ 2-D cores stabilized by Fe(CO)_4_ fragments. Moreover, Ag_4_Au_4_Fe_4_(CO)_16_(dppe)^36^ and Cu_2_Au_6_Fe_4_(CO)_16_(dppe) (dppe = Ph_2_PCH_2_CH_2_PPh_2_)^37^ represent examples of 2-D molecular alloy heteroleptic organomentallic clusters, which have been obtained by condensation of preformed anionic clusters with metal salts.

**Figure 1:**
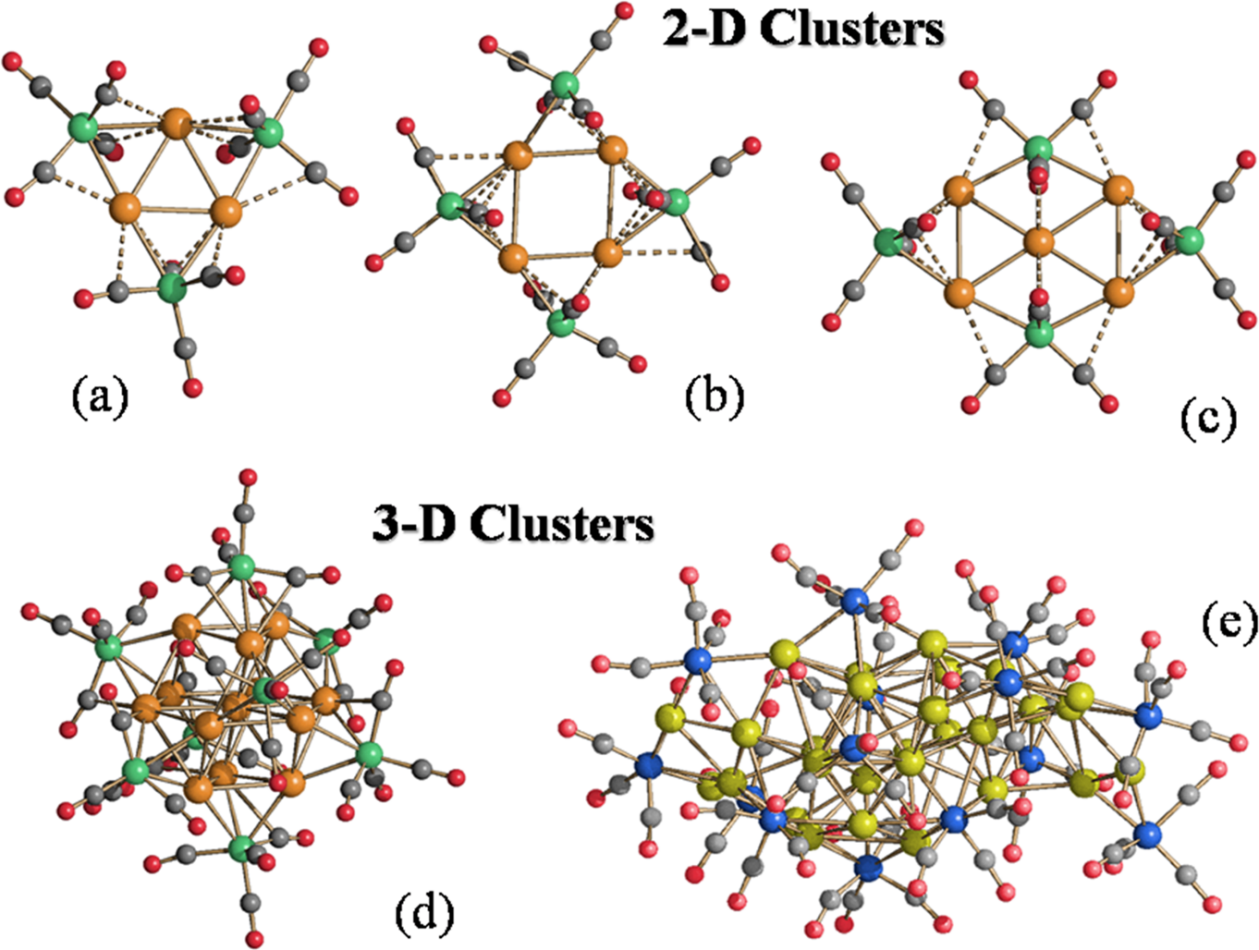
Molecular structures of some representative (a–c) 2-D clusters and (d, e) 3-D clusters. (a) [M_3_Fe_3_(CO)_12_]^3–^ (M = Cu, Ag, Au),^32,33^ (b) [M_4_Fe_4_(CO)_16_]^4–^ (M = Ag, Au),^34,35^ (c) [M_5_Fe_4_(CO)_16_]^3–^ (M = Cu, Ag, Au),^28,32,34^ (d) [Ag_13_Fe_8_(CO)_32_]^3–^,^40,41^ (e) [Au_28_{Fe(CO)_3_}_4_{Fe(CO)_4_}_10_]^8–^.^28^

**Scheme 1:**
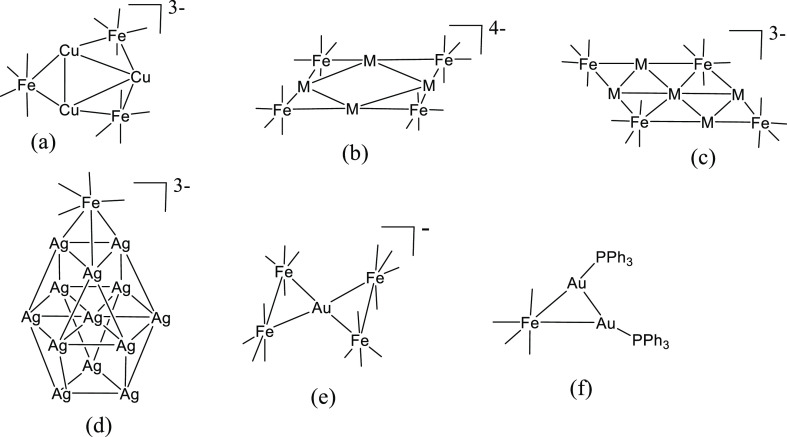
Schematic Structures of the Clusters Discussed in the Paper (a) [Cu_3_Fe_3_(CO)_12_]^3–^. (b) [M_4_Fe_4_(CO)_16_]^4–^ (M = Ag, Au). (c) [M_5_Fe_4_(CO)_16_]^3–^ (M = Cu, Ag, Au). (d) [Ag_13_Fe_8_(CO)_32_]^3–^ (only one Fe(CO)_4_ is represented). (e) [AuFe_4_(CO)_16_]^−^. (f) Fe(CO)_4_(AuPPh_3_)_2_. Carbonyl ligands are represented as lines.

Within this context, 2-D molecular clusters are molecular planar clusters, that is, molecular clusters whose metal atoms lie on the same plane (Figure 1 and Scheme 1). In contrast, 3-D molecular clusters are molecular clusters whose metal atoms form a tridimensional metal core, such as tetrahedron, octahedron, icosahedron, and larger polyhedra or more complex and irregular nonplanar metal aggregates. 2-D molecular clusters are rather interesting,^38,39^ since they represent an alternative to the more common 3-D growth of molecular clusters and nanoparticles. Moreover, they can be viewed as models of metal surfaces and monolayers. Nonetheless, their study is still rather limited compared to 3-D clusters, since their synthesis is not straightforward and, at the moment, general strategies are not available.

Herein, we report a systematic study on [M_*x*_M′_5–*x*_Fe_4_(CO)_16_]^3–^ (M, M′ = Cu, Ag, Au; M ≠ M′; *x* = 0–5) 2-D alloy molecular clusters obtained by a combination of oxidation, condensation, and substitution reactions. Their total structures have been determined by means of single crystal X-ray diffraction (SC-XRD) methods. In addition, they have been investigated by a combination of electron spray ionization mass spectrometry (ESI-MS) and IR and UV–visible spectroscopy. The relative stability of different isomers was rationalized on the basis of DFT calculations.

## 2 Results and Discussion

### 2.1 Synthesis of Ternary [M_*x*_M′_5–*x*_Fe_4_(CO)_16_]^3–^ Clusters (M, M′ = Cu, Ag, Au; M ≠ M′; *x* = 0–5)

**Scheme 2:**
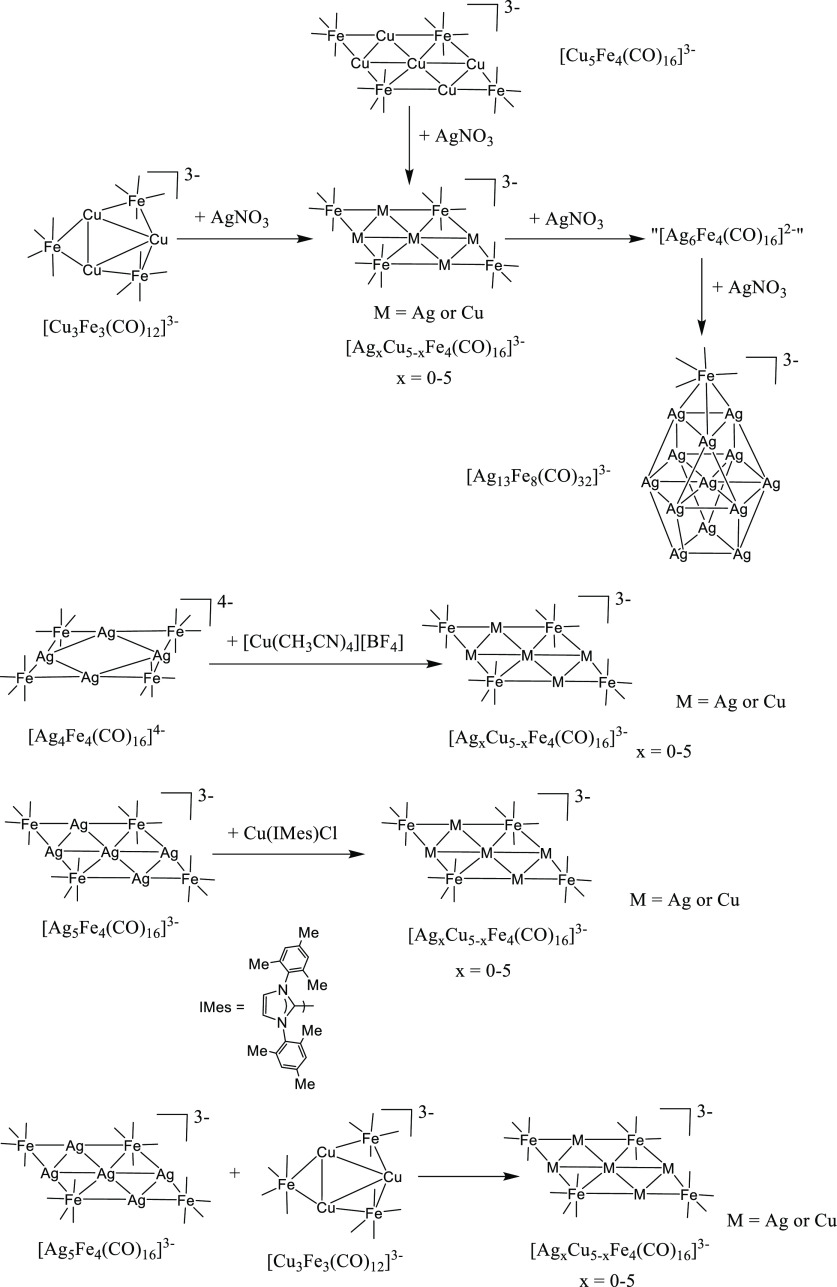
Synthesis of [Ag_*x*_Cu_5–*x*_Fe_4_(CO)_16_]^3–^ All of the reactions have been carried out in CH_3_CN solution at room temperature. The reagents (AgNO_3_, [Cu(CH_3_CN)_4_][BF_4_], Cu(IMes)Cl) have been slowly added to the starting cluster solutions and the reactions monitored through IR spectroscopy. The stoichiometric ratios employed are summarized in Table 1. Complete details are given in the Experimental Section. The structure of “[Ag_6_Fe_4_(CO)_16_]^2–^” is represented in Scheme 3. Only one Fe(CO)_4_ group is included in the schematic representation of [Ag_13_Fe_8_(CO)_32_]^3–^. The complete structure is reported in Figure 1. Carbonyl ligands are represented as lines.

The reactions of [Cu_3_Fe_3_(CO)_12_]^3–^ with increasing amounts of AgNO_3_ resulted in the formation of the ternary [Ag_*x*_Cu_5–*x*_Fe_4_(CO)_16_]^3–^ clusters (Scheme 2 and Table 1), with *x* that increased as more AgNO_3_ was added (up to 1 equiv). This point was evidenced by the fact that the ν_CO_ bands of [Cu_3_Fe_3_(CO)_12_]^3–^ (1921 and 1843 cm^–1^) were replaced by two intense ν_CO_ bands comprised between those of [Cu_5_Fe_4_(CO)_16_]^3–^ (1940 and 1888 cm^–1^) and [Ag_5_Fe_4_(CO)_16_]^3–^ (1949 and 1878 cm^–1^), depending on the Ag content.

**Table 1:** Experimental Conditions for the Synthesis of [NEt_4_]_3_[Ag_*x*_Cu_5–*x*_Fe_4_(CO)_16_] (*x* = 0–5)^a^

			composition of the reagents		composition of the products	
entry		crystallization solvent	Ag	Cu	Ag	Cu
[Cu_3_Fe_3_(CO)_12_]^3–^ + *n*AgNO_3_						
1	*n* = 0.8	CH_3_CN	1.05	3.95	1.02	3.98
2	*n* = 1.3	CH_3_CN	1.51	3.49	5.00	0.00
3		dmf			4.25	0.75
4	*n* = 2.1	CH_3_CN	2.06	2.94	4.88	0.12
5	*n* = 2.3	dmf	2.17	2.83	4.92	0.08
[Cu_5_Fe_4_(CO)_16_]^3–^ + 2.5 AgNO_3_						
6		CH_3_CN	1.67	3.33	5.00	0.00
7		dmf			4.81	0.19
[Ag_4_Fe_4_(CO)_16_]^4–^ + 1.06[Cu(CH_3_CN)_4_][BF_4_]						
8		CH_3_CN	3.95	1.05	4.37	0.63
[Ag_5_Fe_4_(CO)_16_]^3–^ + 3Cu(IMes)Cl (IMes = C_3_N_2_H_2_(C_6_H_2_Me_3_)_2_)						
9		dmf	3.12	1.88	4.90	0.10
[Ag_5_Fe_4_(CO)_16_]^3–^ + [Cu_3_Fe_3_(CO)_12_]^3–^						
10		acetone	3.12	1.88	3.30	1.70
11		CH_3_CN			3.45	1.55

a See Scheme 2.

As a general procedure, the reactions were conducted with different stoichiometric amounts of the reagents ([Cu_3_Fe_3_(CO)_12_]^3–^ and AgNO_3_; [Cu_5_Fe_4_(CO)_16_]^3–^ and AgNO_3_; or other reagents as summarized in Schemes 2, 4, and 5 and Tables 1–3; complete details may be found in the Experimental Section). At the end of the reaction, the solvent was removed under reduced pressure, and the residue was washed with H_2_O and toluene and extracted with solvents of increasing polarity (acetone, CH_3_CN, dmf). The resulting solutions were analyzed by IR spectroscopy and, eventually, layered with an appropriate solvent (acetone/*n*-hexane; CH_3_CN/*n*-hexane/di-iso-propyl-ether; dmf/isopropanol) in the attempt to obtain crystals suitable for X-ray diffraction. Yields have been determined on the isolated crystals, which have been, then, analyzed by SC-XRD, elemental analysis, microwave plasma-atomic emission spectrometry, ESI-MS, and IR and UV–visible spectroscopy.

Formation of [Ag_*x*_Cu_5–*x*_Fe_4_(CO)_16_]^3–^ (*x* = 0–5) under the conditions described above could be explained by considering a combination of oxidation (eq 1), condensation (eq 2), and substitution (eq 3–7) reactions. The occurrence of the substitution reactions was directly confirmed by reacting preformed [Cu_5_Fe_4_(CO)_16_]^3–^ with increasing amounts of AgNO_3_. Also, in these cases, formation of [Ag_*x*_Cu_5–*x*_Fe_4_(CO)_16_]^3–^ (*x* = 0–5) was observed, indicating that Ag^+^ ions can replace Cu^+^ ions within the structure of [Cu_5_Fe_4_(CO)_16_]^3–^. Oxidation/condensation reactions are likely to occur almost in parallel followed by substitution reactions. This point was assessed by ESI-MS analyses (see section 2.3), which clearly revealed the formation at first of [Cu_5_Fe_4_(CO)_16_]^3–^ and [AgCu_4_Fe_4_(CO)_16_]^3–^, followed by [Ag_*x*_Cu_5–*x*_Fe_4_(CO)_16_]^3–^ species richer in Ag (*x* ≥ 2). Formation of [Cu_5_Fe_4_(CO)_16_]^3–^ directly supports the oxidation reaction, as well as the appearance of a silver mirror on the reaction flask accompanied by some gas evolution. The fact that oxidation formally requires more AgNO_3_ than condensation, in accord with eqs 1 and 2, may be explained assuming that there is competition between the two reactions which actually proceed in a parallel way. The stoichiometries depicted in eqs 1 and 2 are those required in the case that a single reaction occurred up to completion. Actually, both reactions start as soon as some AgNO_3_ is added to [Cu_3_Fe_3_(CO)_12_]^3–^. Then, as some [Cu_5_Fe_4_(CO)_16_]^3–^ and [AgCu_4_Fe_4_(CO)_16_]^3–^ are formed, also substitution reactions take place. Assuming that they are all equilibria, the total amount of AgNO_3_ added determines the final distribution of the products as the result of all equilibria. This usually results in one to three species as the main ones present in solution, as evidenced by ESI-MS analyses.

Oxidation:

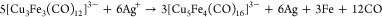
1Condensation:

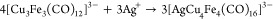
2Substitution:

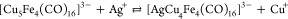
3
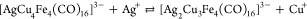
4
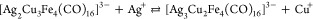
5
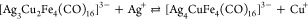
6
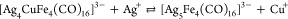
7

**Scheme 3:**
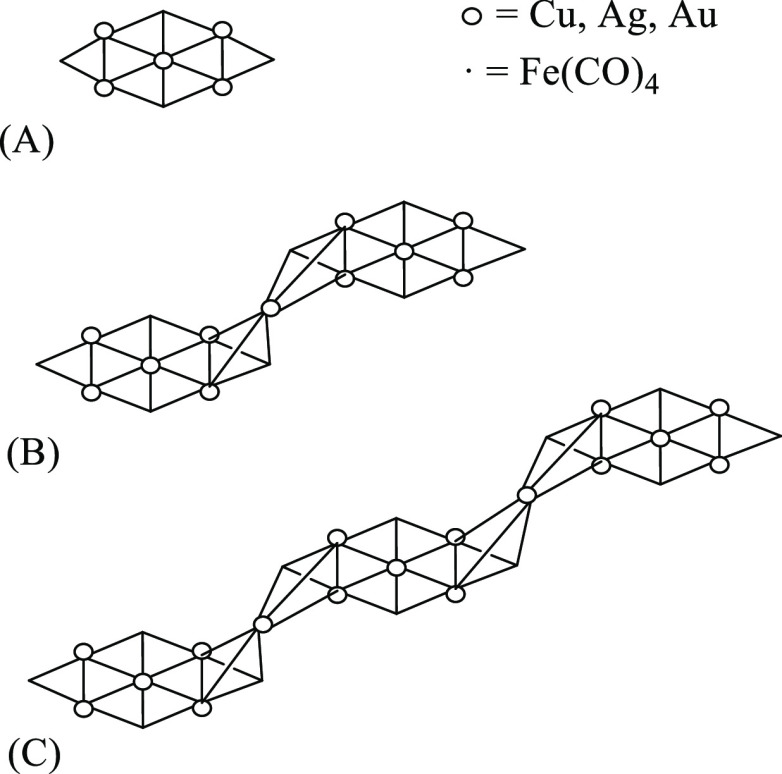
Growth Scheme of the [M_6_Fe_4_(CO)_16_]^2–^ (M = Cu, Ag, Au) Cluster in the Oligomeric Form: (A) [M_5_Fe_4_(CO)_16_]^3–^ Unit; (B) [M{M_5_Fe_4_(CO)_16_}_2_]^5–^ Dimer; (C) [M_2_{M_5_Fe_4_(CO)_16_}_3_]^7–^ Trimer

By adding further AgNO_3_ (>1 equiv per [Cu_3_Fe_3_(CO)_12_]^3–^), two new ν_CO_ bands at 1970 and 1890 cm^–1^, attributable to [Ag_6_Fe_4_(CO)_16_]^2–^,^34,40^ appeared besides those of [Ag_*x*_Cu_5–*x*_Fe_4_(CO)_16_]^3–^ (*x* = 0–5) and rapidly became the major ones. Formation of [Ag_6_Fe_4_(CO)_16_]^2–^ was accompanied by the precipitation of an amorphous solid. Indeed, it was previously reported in the literature that [Ag_6_Fe_4_(CO)_16_]^2–^ was actually a mixture of almost nonsoluble polymeric species (Scheme 3).^34,40^ Their formation was due to different equilibria; some representative ones were depicted in eqs 8–11. Because of such equilibria, it was possible during the workup of these reaction mixtures to extract in polar solvents and, then, crystallize Ag-rich [Ag_*x*_Cu_5–*x*_Fe_4_(CO)_16_]^3–^ (*x* = 0–5) species. Indeed, [Ag_6_Fe_4_(CO)_16_]^2–^ is in equilibrium with [Ag_5_Fe_4_(CO)_16_]^3–^ through eqs 9–11, and in turn, [Ag_5_Fe_4_(CO)_16_]^3–^ is in equilibrium with [Ag_*x*_Cu_5–*x*_Fe_4_(CO)_16_]^3–^ (*x* = 0–5) through eqs 3–7.
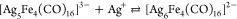
8
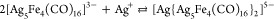
9
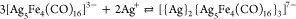
10
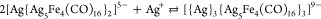
11

Eventually, by employing 2.5 or more equivalents of AgNO_3_ per mole of [Cu_3_Fe_3_(CO)_12_]^3–^, [Ag_13_Fe_8_(CO)_32_]^3–^ was obtained as the final product.^40,41^
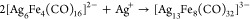
12

Similar results were obtained by employing [Cu_5_Fe_4_(CO)_16_]^3–^ instead of [Cu_3_Fe_3_(CO)_12_]^3–^, or Ag(dppe)(NO_3_) instead of AgNO_3_. During the workup of all of these reactions, it was possible to separate and crystallize several salts of the type [NEt_4_]_3_[Ag_*x*_Cu_5–*x*_Fe_4_(CO)_16_] (*x* = 0–5). As evidenced in Table 1, Ag very rapidly substituted Cu in such clusters, which were always richer in Ag than the reagents. These were the only ternary clusters obtained. For instance, by reacting [Cu_5_Fe_4_(CO)_16_]^3–^ with 3 equiv of Ag(dppe)(NO_3_), crystals of [Cu(dppe)_2_]_3_[Ag_13_Fe_8_(CO)_32_] were obtained, that contained a binary Ag–Fe cluster. A few crystals of Cu_3_Br_3_(dppe)_3_ were also obtained as a side product and mechanically separated from [Cu(dppe)_2_]_3_[Ag_13_Fe_8_(CO)_32_]. The structure of Cu_3_Br_3_(dppe)_3_ as acetone solvate as well as the isostructural Cu_3_Cl_3_(dppe)_3_ were previously reported.^42^

Ternary [Ag_*x*_Cu_5–*x*_Fe_4_(CO)_16_]^3–^ (*x* = 0–5) clusters could also be obtained from the reactions of [Ag_4_Fe_4_(CO)_16_]^4–^ or [Ag_5_Fe_4_(CO)_16_]^3–^ with Cu(I) salts such as [Cu(CH_3_CN)_4_][BF_4_] and Cu(IMes)Cl (IMes = C_3_N_2_H_2_(C_6_H_2_Me_3_)_2_), or by mixing together [Cu_3_Fe_3_(CO)_12_]^3–^ and [Ag_5_Fe_4_(CO)_16_]^3–^. Some representative examples were reported in Table 1 and Scheme 2. [Cu(CH_3_CN)_4_][BF_4_] resulted in being more reactive than Cu(IMes)Cl toward these clusters. Therefore, both reagents have been employed in order to better control the composition of the final [Ag_*x*_Cu_5–*x*_Fe_4_(CO)_16_]^3–^ (*x* = 0–5) clusters. This is a general strategy employed in this work, and other examples are AgNO_3_/Ag(dppe)(NO_3_) and Au(Et_2_S)Cl/Au(PPh_3_)Cl (for each couple, the first reagent is the more reactive one). Indeed, more reactive reagents (AgNO_3_, [Cu(CH_3_CN)_4_][BF_4_], Au(Et_2_S)Cl) favor the substitution of the coinage metal present in the starting cluster with that present in the reagent, whereas substitution proceeds to a minor extent with less reactive reagents (Ag(dppe)(NO_3_), Cu(IMes)Cl, Au(PPh_3_)Cl).

It must be remarked that the fractionary indices of [Ag_*x*_Cu_5–*x*_Fe_4_(CO)_16_]^3–^(*x* = 0–5) indicated that they were actually mixtures of species differing for a few Ag/Cu atoms. Indeed, in some cases, it was possible to separate species with slightly different compositions (entries 2–3, 6–7, and 10–11 in Table 1), by extraction with solvents of different polarities during workup of the same reaction.

**Scheme 4:**
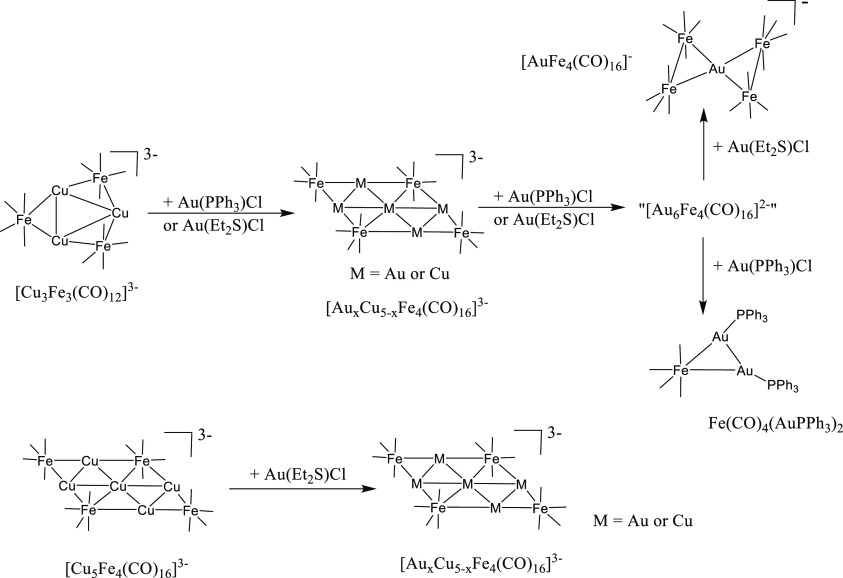
Synthesis of [Au_*x*_Cu_5–*x*_Fe_4_(CO)_16_]^3–^ All of the reactions have been carried out in CH_3_CN solution at room temperature. The reagents (Au(PPh_3_)Cl or Au(Et_2_S)Cl) have been slowly added to the starting cluster solutions and the reactions monitored through IR spectroscopy. The stoichiometric ratios employed are summarized in Table 2. Complete details are given in the Experimental Section. The structure of “[Au_6_Fe_4_(CO)_16_]^2–^” is represented in Scheme 3. Carbonyl ligands are represented as lines.

The reactions of [Cu_3_Fe_3_(CO)_12_]^3–^ and [Cu_5_Fe_4_(CO)_16_]^3–^ with Au(I) salts were very similar to those employing Ag(I) salts (Scheme 4 and Table 2). Thus, ternary [Au_*x*_Cu_5–*x*_Fe_4_(CO)_16_]^3–^ (*x* = 0–5) clusters were formed at first, followed by [Au_6_Fe_4_(CO)_16_]^2–^. Then, further addition of Au(I) resulted in mixtures of “gold browns” such as [Au_21_{Fe(CO)_4_}_10_]^5–^, [Au_22_{Fe(CO)_4_}_12_]^6–^, [Au_28_{Fe(CO)_3_}_4_{Fe(CO)_4_}_10_]^8–^, [Au_34_{Fe(CO)_3_}_6_{Fe(CO)_4_}_8_]^10–^,^28^ and, eventually, [AuFe_4_(CO)_16_]^−^ or Fe(CO)_4_(AuPPh_3_)_2_,^43−45^ depending on the fact that an excess of Au(Et_2_S)Cl or Au(PPh_3_)Cl was used. Also, in this case, [Au_*x*_Cu_5–*x*_Fe_4_(CO)_16_]^3–^ (*x* = 0–5) were the only ternary species isolated.

**Table 2:** Experimental Conditions for the Synthesis of [NEt_4_]_3_[Au_*x*_Cu_5–*x*_Fe_4_(CO)_16_] (*x* = 0–5)^a^

			composition of the reagents		composition of the products	
entry		crystallization solvent	Au	Cu	Au	Cu
[Cu_3_Fe_3_(CO)_12_]^3–^ + *n*Au(PPh_3_)Cl						
12	*n* = 1.4	acetone	1.59	3.41	1.15	3.85
13	*n* = 1.5	acetone	1.67	3.33	1.31	3.69
14		dmf			1.67	3.33
15	*n* = 3.0	dmf	2.5	2.5	2.48	2.52
[Cu_3_Fe_3_(CO)_12_]^3–^ + *n*Au(Et_2_S)Cl						
16	*n* = 0.7	CH_3_CN	0.95	4.05	2.18	2.82
17					2.73	2.27
18	*n* = 1.9	CH_3_CN	1.94	3.06	4.59	0.41
19					4.62	0.38
[Cu_5_Fe_4_(CO)_16_]^3–^ + 1.2Au(Et_2_S)Cl						
20		acetone	0.97	4.03	1.09	3.91

a See Scheme 4.

Replacement of Cu with Au in [Au_*x*_Cu_5–*x*_Fe_4_(CO)_16_]^3–^ (*x* = 0–5) was more gradual than in the related Ag–Cu–Fe clusters. As a consequence, it was possible to obtain species with a more continuous distribution of the two metals (Table 2). Moreover, Cu-substitution was favored by using Au(Et_2_S)Cl compared to Au(PPh_3_)Cl. This might be due to the fact that the phosphine complex was less reactive.

**Scheme 5:**
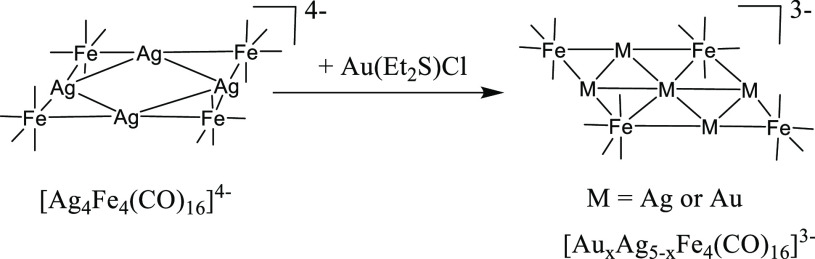
Synthesis of [Au_*x*_Ag_5–*x*_Fe_4_(CO)_16_]^3–^ All of the reactions have been carried out in CH_3_CN solution at room temperature. The reagent (Au(Et_2_S)Cl) has been slowly added to the starting cluster solutions and the reactions monitored through IR spectroscopy. The stoichiometric ratios employed are summarized in Table 3. Complete details are given in the Experimental Section. Carbonyl ligands are represented as lines.

Ternary [Au_*x*_Ag_5–*x*_Fe_4_(CO)_16_]^3–^ clusters (*x* = 0–5) were, then, obtained from the reaction of [Ag_4_Fe_4_(CO)_16_]^4–^ with Au(Et_2_S)Cl as a representative example (Scheme 5 and Table 3).

**Table 3:** Experimental Conditions for the Synthesis of [NEt_4_]_3_[Au_*x*_Ag_5–*x*_Fe_4_(CO)_16_] (*x* = 0–5)^a^

			composition of the reagents		composition of the products	
entry		crystallization solvent	Au	Ag	Au	Ag
[Ag_4_Fe_4_(CO)_16_]^4–^ + 0.8Au(Et_2_S)Cl						
21		CH_3_CN	0.83	4.17	0.64	4.36
22		acetone			0.81	4.19

a See Scheme 5.

The IR spectra of the ternary [M_*x*_M′_5–*x*_Fe_4_(CO)_16_]^3–^ (*x* = 0–5; M, M′ = Cu, Ag, Au; M ≠ M′) clusters show two almost equally intense ν_CO_ bands in the regions 1942–1950 and 1878–1894 cm^–1^ for [Ag_*x*_Cu_5–*x*_Fe_4_(CO)_16_]^3–^, 1945–1951 and 1870–1887 cm^–1^ for [Au_*x*_Cu_5–*x*_Fe_4_(CO)_16_]^3–^, and 1946–1950 and 1874–1880 cm^–1^ for [Au_*x*_Ag_5–*x*_Fe_4_(CO)_16_]^3–^ (see Figures S1–S9 in the Supporting Information). Similar spectral features have been reported for the related binary clusters, that is, 1940 and 1888 cm^–1^ for [Cu_5_Fe_4_(CO)_16_]^3–^, 1949 and 1878 cm^–1^ for [Ag_5_Fe_4_(CO)_16_]^3–^, and 1944 and 1861 cm^–1^ for [Au_5_Fe_4_(CO)_16_]^3–^. All of the CO ligands are terminally bonded within Fe(CO)_4_ groups. The presence of only two intense ν_CO_ stretching bands in these 2-D (planar) clusters containing an Fe(CO)_4_ group has been explained by application of a spherical harmonic model (SHM) to their ν_CO_ vibrational spectra.^46^ The reader may find in the cited literature full theoretical details on why SHM rather classical symmetry rules (group theory) is needed in order to interpret the IR spectra in the ν_CO_ region of these 2-D clusters. Overall, SHM correctly predicts the presence of two strong ν_CO_ bands in the IR spectra of such clusters. The same applies to the parent 2-D clusters [Cu_3_Fe_3_(CO)_12_]^3–^ (1921 and 1843 cm^–1^), [Ag_4_Fe_4_(CO)_16_]^4–^ (1928 and 1852 cm^–1^), and [Au_4_Fe_4_(CO)_16_]^4–^ (1929 and 1864 cm^–1^), as well as [Ag_6_Fe_4_(CO)_16_]^2–^ (1970 and 1890 cm^–1^) and [Au_6_Fe_4_(CO)_16_]^2–^ (1976 and 1906 cm^–1^). Thus, all of these 2-D clusters, regardless of the number of M atoms and Fe(CO)_4_ groups, display very similar IR spectra on the ν_CO_ region, showing two intense bands that differ only in their frequencies. In turn, the observed stretching frequencies mainly depend on the ratio between the overall charge of the cluster and the number of metal atoms, and only to a lesser extent on the nature of the coinage metals present.

### 2.2 Molecular Structures of [M_*x*_M′_5–*x*_Fe_4_(CO)_16_]^3–^ (M, M′ = Cu, Ag, Au; M ≠ M′; *x* = 0–5)

**Figure 2:**
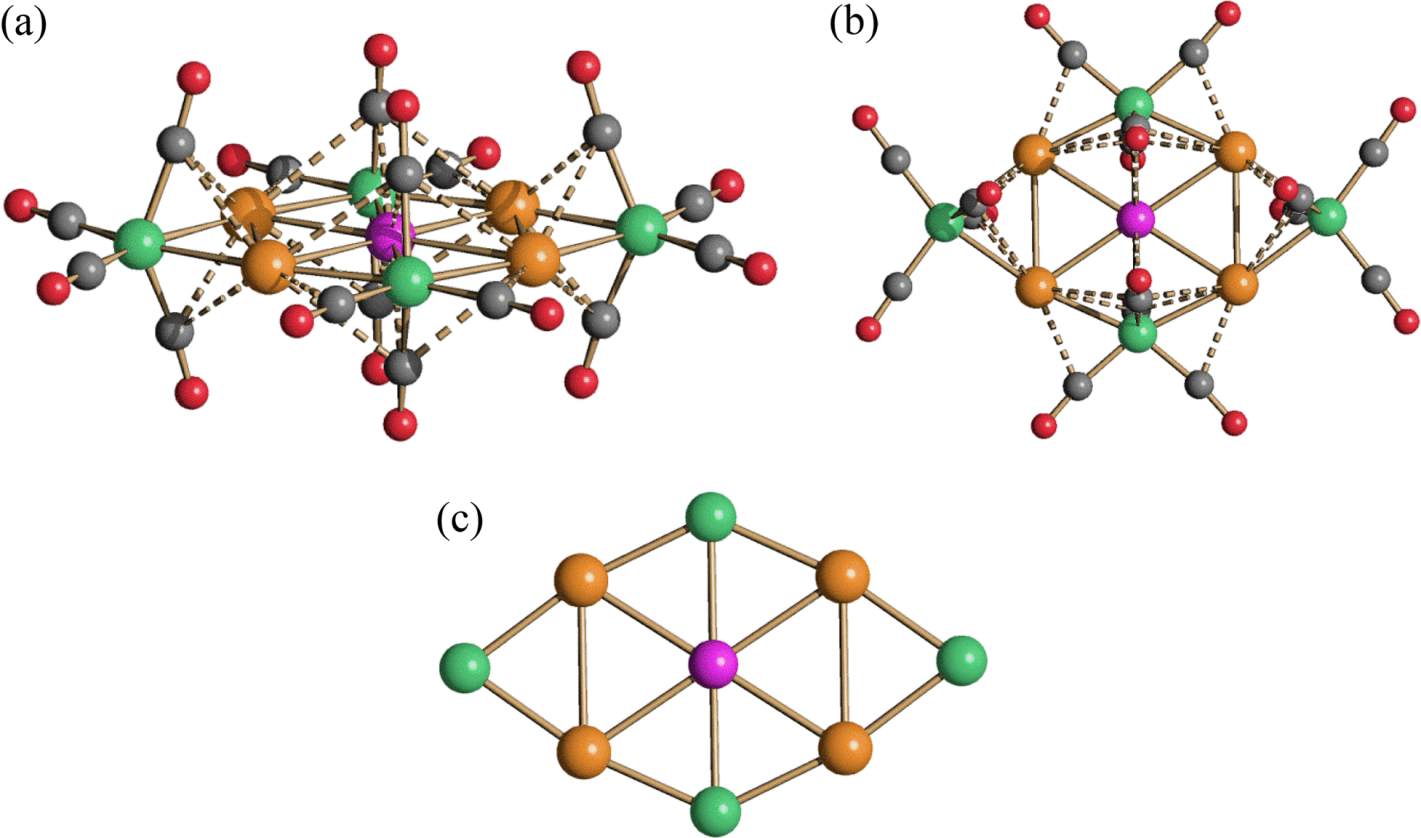
Molecular structure of the [M_*x*_M′_5–*x*_Fe_4_(CO)_16_]^3–^ (*x* = 0–5; M, M′ = Cu, Ag, Au; M ≠ M′) clusters (purple, M in the center; orange, M in the corner positions; green, Fe; gray, C; red, O). M–C(O) contacts are represented as fragmented lines. Two different views are reported (a, b), as well as the metal core (c).

The molecular structure common to all ternary [M_*x*_M′_5–*x*_Fe_4_(CO)_16_]^3–^ (*x* = 0–5; M, M′ = Cu, Ag, Au; M ≠ M′) clusters is based on a centered M_5_ rectangle (Figure 2), as previously found also in the binary [M_5_Fe_4_(CO)_16_]^3–^ (M = Cu, Ag, Au) clusters.^28,32,34,47^ The positions occupied by the five coinage metals can be grouped into two sites: (a) the unique central position; (b) the four equivalent corner positions. This M_5_ core is bonded to two μ-Fe(CO)_4_ and two μ_3_-Fe(CO)_4_ groups on the shorter and longer edges of the rectangle, respectively. Overall, the M atom at the center forms four M_center_–M_corner_ bonds and two M–Fe bonds, whereas M atoms at the corner sites form one M_center_–M_corner_ bond, one M_corner_–M_corner_ bond, and two M–Fe bonds. The Fe–M_corner_–Fe coordination is almost linear, as expected for a d^10^ M(I) ion. Some sub van der Waals M–C(O) contacts are also present, but their nature in these and related clusters is rather debated.^48,49^ Theoretical calculations reported in the literature mainly point out that the presence of such contacts in the solid state structures of Fe carbonyl clusters containing also coinage metals is essentially the consequence of steric requirements and not real bonds or even any (even weak) attraction. Thus, the preferential arrangement of the CO ligands about the Fe center brings the carbonyls in closer proximity to the M (Cu, Ag, Au) center, without any real interaction that could be spectroscopically evidenced.

The fractional indices present in the formulas of [M_*x*_M′_5–*x*_Fe_4_(CO)_16_]^3–^ (*x* = 0–5; M, M′ = Cu, Ag, Au; M ≠ M′) are indicative of compositional disorder; that is, the crystals contain mixtures of species. For instance, [Ag_4.25_Cu_0.75_Fe_4_(CO)_16_]^3–^ corresponds to a mixture of [Ag_4_CuFe_4_(CO)_16_]^3–^ (75%) and [Ag_5_Fe_4_(CO)_16_]^3–^ (25%), whereas [Au_2.48_Cu_2.52_Fe_4_(CO)_16_]^3–^ contains [Au_2_Cu_3_Fe_4_(CO)_16_]^3–^ (52%) and [Au_3_Cu_2_Fe_4_(CO)_16_]^3–^ (48%).

In addition, substitutional disorder is always observed, since M and M′ are (not equivalently) disordered over the central and corner positions (Tables 4–6). In the case of Au–Cu clusters, Au strongly prefers the corner sites and Cu the central position. This trend is observed, even if to a less extent, also in Ag–Cu clusters. In the case of Au–Ag clusters, even if the number of entries is very limited, it is possible to notice a Au preference for the corner sites.

**Table 4:** Composition of [NEt_4_]_3_[Ag_*x*_Cu_5–*x*_Fe_4_(CO)_16_] (*x* = 0–5)

	total		centre		corner	
entry^a^	Ag	Cu	Ag	Cu	Ag	Cu
1	1.02	3.98	0.02	0.98	1.00	3.00
10	3.30	1.70	0.60	0.40	2.70	1.30
11	3.45	1.55	0.59	0.41	2.86	1.14
3	4.25	0.75	0.83	0.17	3.42	0.58
8	4.37	0.63	0.85	0.15	3.52	0.48
7	4.81	0.19	0.97	0.03	3.84	0.16
4	4.88	0.12	0.97	0.03	3.92	0.08
9	4.90	0.10	0.98	0.02	3.92	0.08
5	4.92	0.08	1.00	0.00	3.92	0.08
2,6	5.00	0.00	1.00	0.00	4.00	0.00

a See Table 1. Entries are listed in order of increasing Ag content.

**Table 5:** Composition of [NEt_4_]_3_[Au_*x*_Cu_5–*x*_Fe_4_(CO)_16_] (*x* = 0–5)

	total		centre		corner	
entry^a^	Au	Cu	Au	Cu	Au	Cu
20	1.09	3.91	0.00	1.00	1.09	2.91
12	1.15	3.85	0.00	1.00	1.15	2.85
13	1.31	3.69	0.00	1.00	1.31	2.69
14	1.67	3.33	0.00	1.00	1.67	2.33
16	2.18	2.82	0.00	1.00	2.18	1.82
15	2.48	2.52	0.00	1.00	2.48	1.52
17	2.73	2.27	0.00	1.00	2.73	1.27
18	4.59	0.41	0.59	0.41	4.00	0.00
19	4.62	0.38	0.61	0.39	4.00	0.00

a See Table 2. Entries are listed in order of increasing Au content.

**Table 6:** Composition of [NEt_4_]_3_[Au_*x*_Ag_5–*x*_Fe_4_(CO)_16_] (*x* = 0–5)

	total		centre		corner	
entry^a^	Au	Ag	Au	Ag	Au	Ag
21	0.64	4.36	0.04	0.96	0.60	3.40
22	0.81	4.19	0.05	0.95	0.76	3.24

a See Table 3. Entries are listed in order of increasing Au content.

The preference of Cu for the central position may be, at least partially, explained on the basis of the different ionic radii of the M(I) cations: Cu(I) 77 pm, Ag(I) 115 pm, and Au(I) 137 pm. Thus, the smallest Cu(I) ion prefers the central site. Moreover, corner positions display two strong M–Fe bonds with an almost linear Fe–M–Fe arrangement. This is the typical coordination found in M(I) complexes. The high affinity of Au for these corner sites indicates a stronger stability of such interactions in the case of Au compared to Cu and Ag.

**Figure 3:**
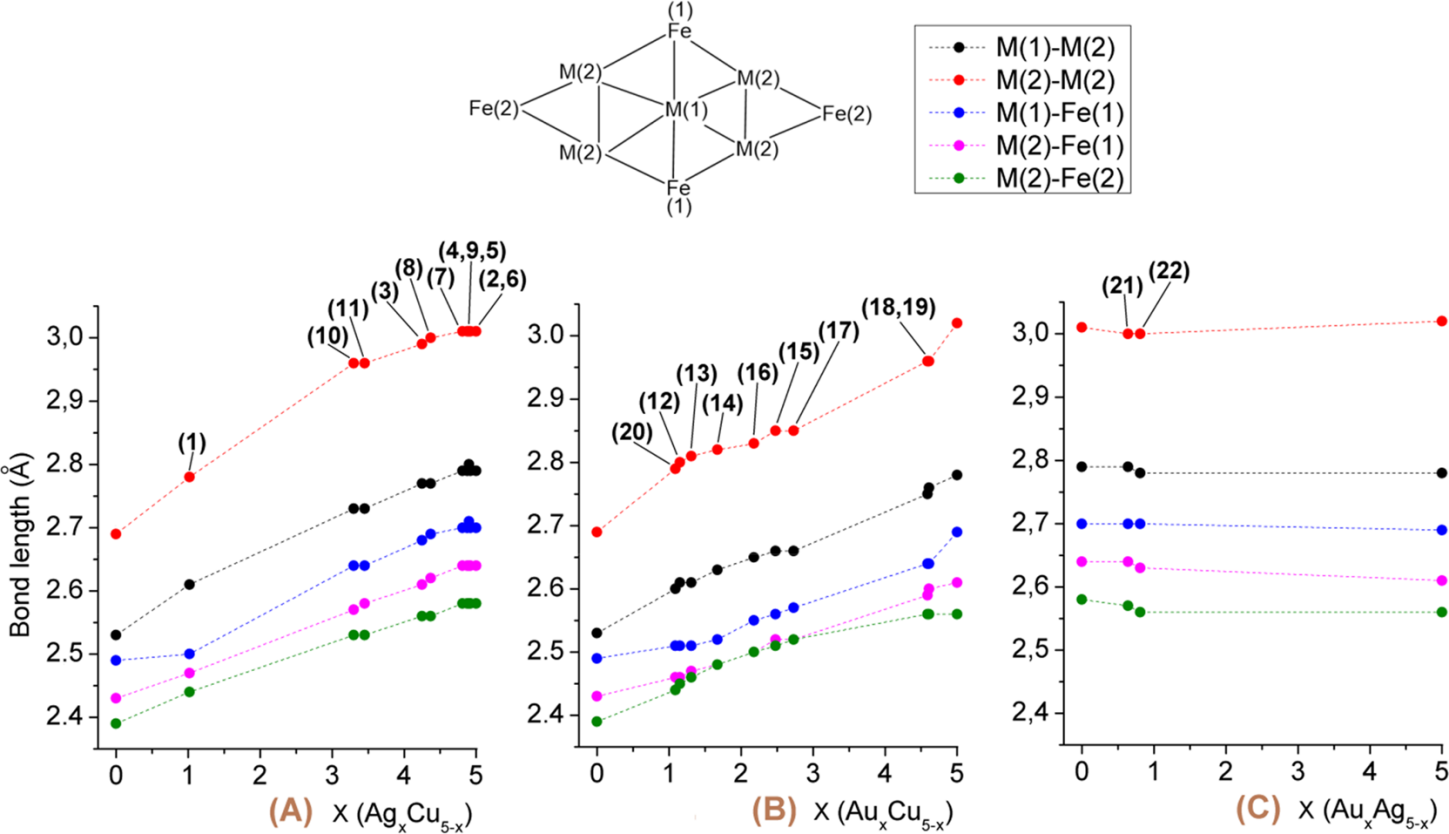
M–M and M–Fe distances (Å) of (A) [NEt_4_]_3_[Ag_*x*_Cu_5–*x*_Fe_4_(CO)_16_]; (B) [NEt_4_]_3_[Au_*x*_Cu_5–*x*_Fe_4_(CO)_16_]; (C) [NEt_4_]_3_[Au_*x*_Ag_5–*x*_Fe_4_(CO)_16_]. Entries are reported in parentheses. M(1)–M(2), black; M(2)–M(2), red; M(1)–Fe(1), blue; M(2)–Fe(1), magenta; M(2)–Fe(2), green. See the scheme in the inset for the numbering.

As expected, M_center_–M_corner_ [M(1)–M(2) in Figure 3], M_corner_–M_corner_ [M(2)–M(2) in Figure 3], and M–Fe distances [M(1)–Fe(1), M(2)–Fe(1), and M(2)–Fe(2) in Figure 3] steadily increase moving from Cu-rich to Ag- or Au-rich clusters (Figure 3; Tables S1–S3 and Scheme S1 in the Supporting Information). This is well exemplified by comparing the M–M and M–Fe distances of ternary [M_*x*_M′_5–*x*_Fe_4_(CO)_16_]^3–^ (*x* = 0–5; M, M′ = Cu, Ag, Au; M ≠ M′) and binary [M_5_Fe_4_(CO)_16_]^3–^ (M = Cu, Ag, Au) clusters. Indeed, [Cu_5_Fe_4_(CO)_16_]^3–^ displays considerably shorter distances, whereas these are almost identical in [Ag_5_Fe_4_(CO)_16_]^3–^ and [Au_5_Fe_4_(CO)_16_]^3–^.

### 2.3 ESI-MS Studies of [M_*x*_M′_5–*x*_Fe_4_(CO)_16_]^3–^ (M, M′ = Cu, Ag, Au; M ≠ M′; *x* = 0–5)

In order to further elucidate the composition of ternary [M_*x*_M′_5–*x*_Fe_4_(CO)_16_]^3–^ clusters, ESI-MS studies have been carried out on some of their [NEt_4_]_3_[M_*x*_M′_5–*x*_Fe_4_(CO)_16_] crystals. The ESI-MS spectra recorded on CH_3_CN solutions of [NEt_4_]_3_[Ag_1.02_Cu_3.98_Fe_4_(CO)_16_], [NEt_4_]_3_[Au_1.32_Cu_3.68_Fe_4_(CO)_16_], [NEt_4_]_3_[Au_2.48_Cu_2.52_Fe_4_(CO)_16_], [NEt_4_]_3_[Au_4.62_Cu_0.38_Fe_4_(CO)_16_], and [NEt_4_]_3_[Au_0.82_Ag_4.18_Fe_4_(CO)_16_] are reported in Figures S14–S37 in the Supporting Information (including calculated fits of the prominent peaks), and peak assignments are summarized in Tables S6–S10. For the sake of comparison, the ESI-MS spectra of binary [NEt_4_]_3_[Cu_5_Fe_4_(CO)_16_] and [NEt_4_]_3_[Ag_5_Fe_4_(CO)_16_] clusters are also reported (Figures S10–S13 and Tables S4 and S5 in the Supporting Information). It has not been possible to investigate [NEt_4_]_3_[Au_5_Fe_4_(CO)_16_], since it is irreversibly oxidized during the dilution required for ESI-MS analyses. The peak assignments have been corroborated by comparing their experimental isotopic patterns with the theoretical ones based on the formulas.

Under ESI-MS conditions, the [M_*x*_M′_5–*x*_Fe_4_(CO)_16_]^3–^ trianionic clusters were systematically detected as dianions. This might be explained assuming either oxidation of [M_*x*_M′_5–*x*_Fe_4_(CO)_16_]^3–^ to [M_*x*_M′_5–*x*_Fe_4_(CO)_16_]^2–^ or addition of H^+^ via electrostatic interaction resulting in [HM_*x*_M′_5–*x*_Fe_4_(CO)_16_]^2–^ ions. Comparison of the experimental and calculated isotopic patterns of the most prominent peaks (Figures S10–S37 in the Supporting Information) suggests that formation of [HM_*x*_M′_5–*x*_Fe_4_(CO)_16_]^2–^ adducts is more likely, even if the detection of a single hydrogen atom in such large clusters is at the limit of the precision of the instrument employed for these analyses. Formation of [HM_*x*_M′_5–*x*_Fe_4_(CO)_16_]^2–^ adducts via electrostatic interaction is further supported by the fact that, in some cases, also the {[M_*x*_M′_5–*x*_Fe_4_(CO)_16_][NEt_4_]}^2–^ adducts involving [NEt_4_]^+^ instead of H^+^ were present in the spectra.

The dianionic nature of all of these ions is confirmed by the systematic loss of *m*/*z* 14 units from the molecular ions, that corresponds to a CO ligand (28 amu) assuming *z* = 2. It must be remarked that CO loss is almost absent in the case of binary [Cu_5_Fe_4_(CO)_16_]^3–^ and [Ag_5_Fe_4_(CO)_16_]^3–^ clusters. Therefore, CO loss in the gas phase of ternary [M_*x*_M′_5–*x*_Fe_4_(CO)_16_]^3–^ clusters may be viewed as an alloy effect due to the contemporary presence of two different coinage metals within their (M, M′)_5_ core.

The ESI-MS spectrum (ES−) of [NEt_4_]_3_[Au_2.48_Cu_2.52_Fe_4_(CO)_16_] (Figures S24–S29 and Table S8 in the Supporting Information) displays two sets of peaks at *m*/*z* (relative intensities in parentheses): 759(30), 694(60), 680(10), 666(5), 652(10), 638(100), and 626(10) (**set-1-Au**_**3**_**Cu**_**2**_); 692(35), 628(60), 614(5), 600(30), 586(60), 572(80), and 558(15) (**set-2-Au**_**2**_**Cu**_**3**_). The peaks of **set-1-Au**_**3**_**Cu**_**2**_ originate from the [HAu_3_Cu_2_Fe_4_(CO)_16_]^2–^ ion (*m*/*z* = 694) by the stepwise loss of one to five CO ligands (*m*/*z* = 680, 666, 652, 638, 626), whereas the peak at *m*/*z* 759 corresponds to the {[Au_3_Cu_2_Fe_4_(CO)_16_][NEt_4_]}^2–^ adduct. Similarly, the peaks of **set-2-Au**_**2**_**Cu**_**3**_ originate from the [HAu_2_Cu_3_Fe_4_(CO)_16_]^2–^ ion (*m*/*z* = 628) by the stepwise loss of one to five CO ligands (*m*/*z* = 614, 600, 586, 572, 558), whereas the peak at *m*/*z* 692 corresponds to the {[Au_2_Cu_3_Fe_4_(CO)_16_][NEt_4_]}^2–^ adduct. Overall, the ESI-MS analysis indicates that two species of composition Au_3_Cu_2_ and Au_2_Cu_3_ are present in similar amounts, in agreement with the X-ray data which suggest that [NEt_4_]_3_[Au_2.48_Cu_2.52_Fe_4_(CO)_16_] contains 48% of [Au_3_Cu_2_Fe_4_(CO)_16_]^3–^ and 52% of [Au_2_Cu_3_Fe_4_(CO)_16_]^3–^.

Similar considerations apply to [NEt_4_]_3_[Au_4.62_Cu_0.38_Fe_4_(CO)_16_] (Figures S30–S32 and Table S9 in the Supporting Information), for which the X-ray data suggest a mixture of 62% of [Au_5_Fe_4_(CO)_16_]^3–^ and 38% of [Au_4_CuFe_4_(CO)_16_]^3–^. Indeed, the ESI-MS spectrum shows two sets of peaks attributable to a Au_5_ (**set-1-Au**_**5**_) and Au_4_Cu (**set-2-Au**_**4**_**Cu**) species. The first set (**set-1-Au**_**5**_) displays peaks at *m*/*z* 893(30), 828(30), 814(45), 800(2), and 772(80) attributable to the {[Au_5_Fe_4_(CO)_16_][NEt_4_]}^2–^ adduct, the [HAu_5_Fe_4_(CO)_16_]^2–^ parent ion, and the stepwise loss of one to four CO ligands. It must be remarked that the peak corresponding to the loss of three carbonyls is probably too weak to be detected. Conversely, the second set (**set-2-Au**_**4**_**Cu**) shows peaks at *m*/*z* 826(30), 761(40), 747(20), 730(70), 719(10), and 705(100) attributable to {[Au_5_Fe_4_(CO)_16_][NEt_4_]}^2–^, [HAu_5_Fe_4_(CO)_16_]^2–^, and the stepwise loss of one to four CO ligands.

The ESI-MS spectra of [NEt_4_]_3_[Ag_1.02_Cu_3.98_Fe_4_(CO)_16_], [NEt_4_]_3_[Au_1.32_Cu_3.68_Fe_4_(CO)_16_], and [NEt_4_]_3_[Au_0.82_Ag_4.18_Fe_4_(CO)_16_] (Figures S14–S23 and S33–S37 and Tables S6, S7, and S10 in the Supporting Information) contain three sets of peaks instead of two sets as above and suggest a slightly more complicated disorder (compositional) model. For instance, [NEt_4_]_3_[Ag_1.02_Cu_3.98_Fe_4_(CO)_16_] (Figures S14–S19 and Table S6 in the Supporting Information) could have been interpreted as a mixture of 2% [Ag_2_Cu_3_Fe_4_(CO)_16_]^3–^ and 98% [AgCu_4_Fe_4_(CO)_16_]^3–^ on the basis of SC-XRD data. Conversely, its ESI-MS spectrum displays three sets of peaks (**set-1-Ag**_**2**_**Cu**_**3**_, **set-2-AgCu**_**4**_, and **set-3-Cu**_**5**_) attributable to the ionization of these two species as well as an additional [Cu_5_Fe_4_(CO)_16_]^3–^ cluster. Indeed, **set-1-Ag**_**2**_**Cu**_**3**_ includes peaks at *m*/*z* 604(40), 539(60), 525(10), 510(50), and 497(30) attributable to {[Ag_2_Cu_3_Fe_4_(CO)_16_][NEt_4_]}^2–^, [HAg_2_Cu_3_Fe_4_(CO)_16_]^2–^, and the stepwise loss of one to three carbonyls. **Set-2-AgCu**_**4**_ shows peaks at *m*/*z* 582(60), 517(90), 503(10), 488(100), 474(65), 460(5), and 447(70) attributable to {[AgCu_4_Fe_4_(CO)_16_][NEt_4_]}^2–^, [HAgCu_4_Fe_4_(CO)_16_]^2–^, and the stepwise loss of one to five CO ligands. Then, **set-3-Cu**_**5**_ comprises peaks at *m*/*z* 560(35), 495(50), 482(15), 466(75), and 452(60) attributable to {[Cu_5_Fe_4_(CO)_16_][NEt_4_]}^2–^, [HCu_5_Fe_4_(CO)_16_]^2–^, and the stepwise loss of one to three carbonyls. The presence of [Cu_5_Fe_4_(CO)_16_]^3–^ in the mixture lends support to the occurrence of the initial oxidation reaction depicted in eq 1 (see section 2.1), whereas the presence of [AgCu_4_Fe_4_(CO)_16_]^3–^ is mainly due to the condensation reaction (eq 2). Then, [Ag_2_Cu_3_Fe_4_(CO)_16_]^3–^ is formed via subsequent substitution (eq 3).

Similarly, the ESI-MS spectrum of [NEt_4_]_3_[Au_1.32_Cu_3.68_Fe_4_(CO)_16_] (Figures S19–S23 and Table S7 in the Supporting Information) suggests the presence of the three species [Au_2_Cu_3_Fe_4_(CO)_16_]^3–^, [AuCu_4_Fe_4_(CO)_16_]^3–^, and [Cu_5_Fe_4_(CO)_16_]^3–^, as indicated by the presence of three sets of peaks. Three sets of peaks are present also in the case of [NEt_4_]_3_[Au_0.82_Ag_4.18_Fe_4_(CO)_16_] (Figures S33–S37 and Table S10 in the Supporting Information), suggesting a mixture of [Au_2_Ag_3_Fe_4_(CO)_16_]^3–^, [AuAg_4_Fe_4_(CO)_16_]^3–^, and [Ag_5_Fe_4_(CO)_16_]^3–^. Also, in these cases, the distribution of the products is in agreement with the occurrence of oxidation, condensation, and substitution reactions.

On the basis of both SC-XRD and ESI-MS data, it is possible to conclude that the solid state structures of [M_*x*_M′_5–*x*_Fe_4_(CO)_16_]^3–^ consist of mixtures of two or three species differing for one to two coinage metals in the metal core of the cluster.

It is also noteworthy that the [M_*x*_M′_5–*x*_Fe_4_(CO)_16_]^3–^ clusters under ESI-MS conditions show a high tendency to lose one to four CO ligands, sometimes even five carbonyls. This suggests that in the gas phase their M_*x*_M′_5–*x*_ cores could be stabilized by both Fe(CO)_4_ and Fe(CO)_3_ groups, whereas in the solid state only the former is observed. This is not the case for binary [M_5_Fe_4_(CO)_16_]^3–^ clusters, suggesting that the tendency to lose CO ligands is somehow an alloy effect.

### 2.4 UV–Visible Studies of [M_*x*_M′_5–*x*_Fe_4_(CO)_16_]^3–^ (M, M′ = Cu, Ag, Au; M ≠ M′; *x* = 0–5)

**Figure 4:**
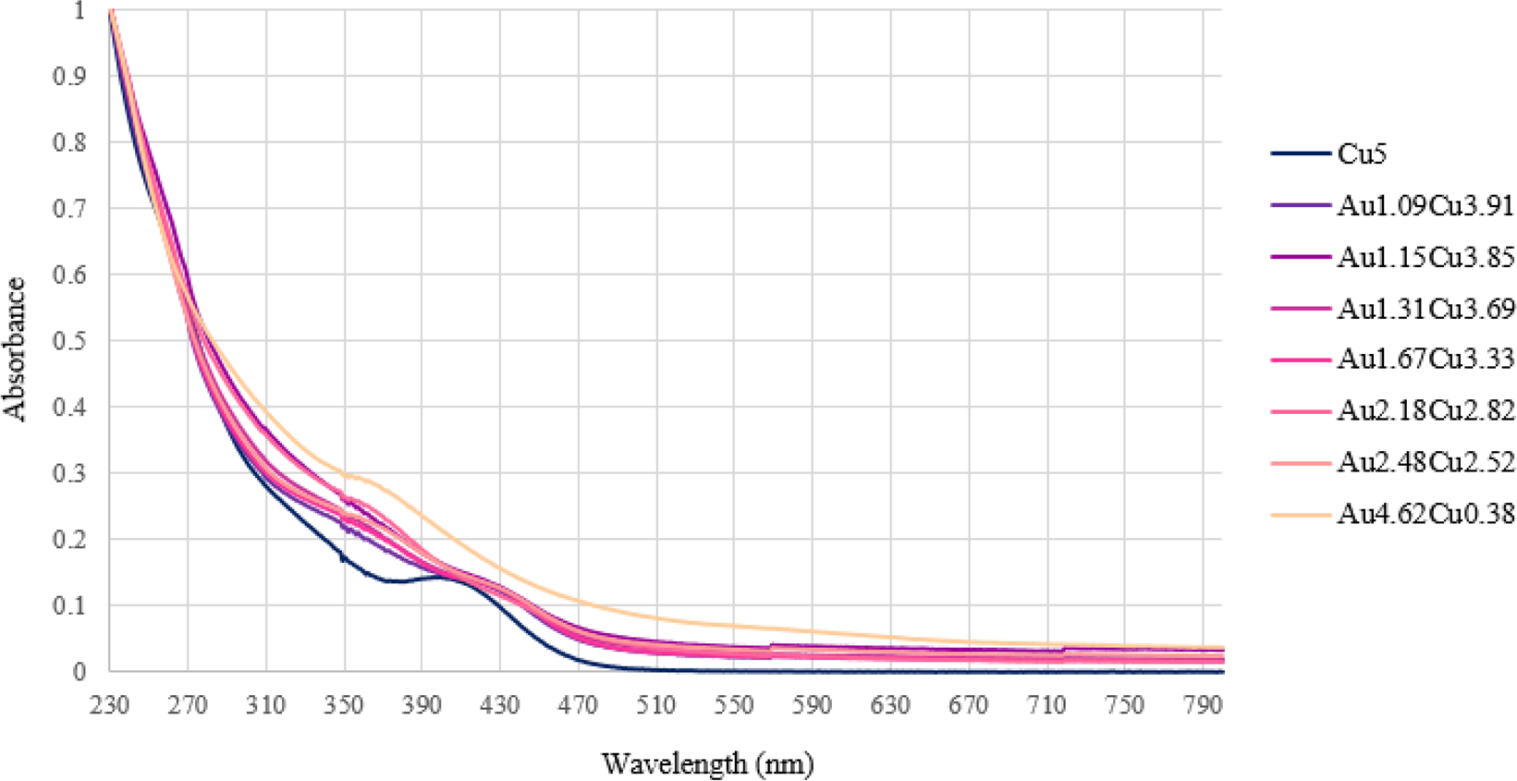
UV–visible absorption spectra of [NEt_4_]_3_[Au_*x*_Cu_5–*x*_Fe_4_(CO)_16_] in CH_3_CN at 298 K (concentration 1.25 × 10^–5^ M). Cu5 = [Cu_5_Fe_4_(CO)_16_]^3–^; Au1.09Cu3.91 = [Au_1.09_Cu_3.91_Fe_4_(CO)_16_]^3–^; Au1.15Cu3.85 = [Au_1.15_Cu_3.85_Fe_4_(CO)_16_]^3–^; Au1.31Cu3.69 = [Au_1.31_Cu_3.69_Fe_4_(CO)_16_]^3–^; Au1.67Cu3.33 = [Au_1.67_Cu_3.33_Fe_4_(CO)_16_]^3–^; Au2.18Cu2.82 = [Au_2.18_Cu_2.82_Fe_4_(CO)_16_]^3–^; Au2.48Cu2.52 = [Au_2.48_Cu_2.52_Fe_4_(CO)_16_]^3–^; Au4.62Cu0.38 = [Au_4.62_Cu_0.38_Fe_4_(CO)_16_]^3–^.

**Figure 5:**
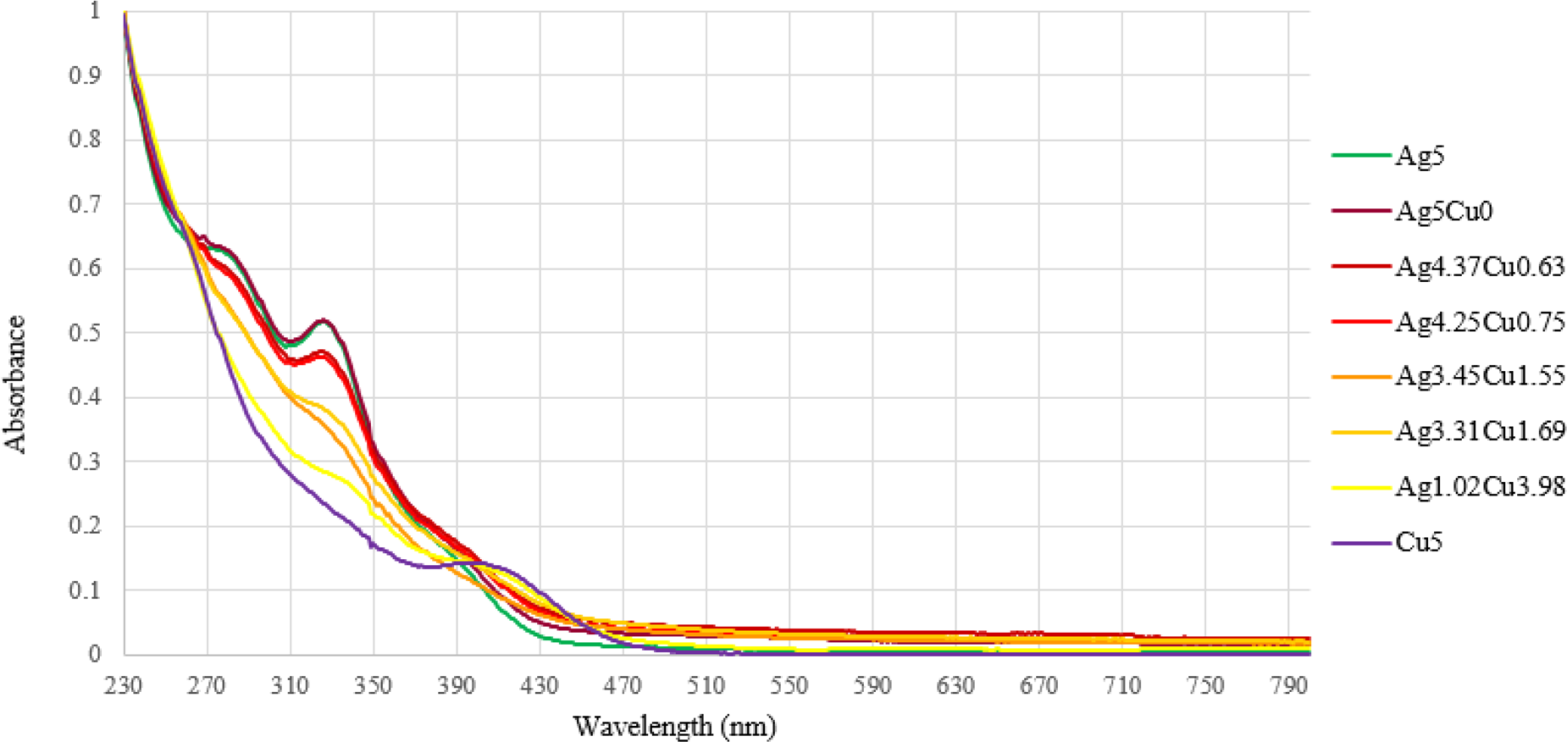
UV–visible absorption spectra of [NEt_4_]_3_[Ag_*x*_Cu_5–*x*_Fe_4_(CO)_16_] in CH_3_CN at 298 K (concentration 1.25 × 10^–5^ M). Ag5 = [Ag_5_Fe_4_(CO)_16_]^3–^; Ag5Cu0 = [Ag_5_Cu_0_Fe_4_(CO)_16_]^3–^ (see entry 2 in Table 1); Ag4.37Cu0.63 = [Ag_4.37_Cu_0.63_Fe_4_(CO)_16_]^3–^; Ag4.25Cu0.75 = [Ag_4.25_Cu_0.753_Fe_4_(CO)_16_]^3–^; Ag3.45Cu1.55 = [Ag_3.45_Cu_1.55_Fe_4_(CO)_16_]^3–^; Ag3.31Cu1.69 = [Ag_3.31_Cu_1.69_Fe_4_(CO)_16_]^3–^; Ag1.02Cu3.98 = [Ag_1.02_Cu_3.98_Fe_4_(CO)_16_]^3–^; Cu5 = [Cu_5_Fe_4_(CO)_16_]^3–^.

**Figure 6:**
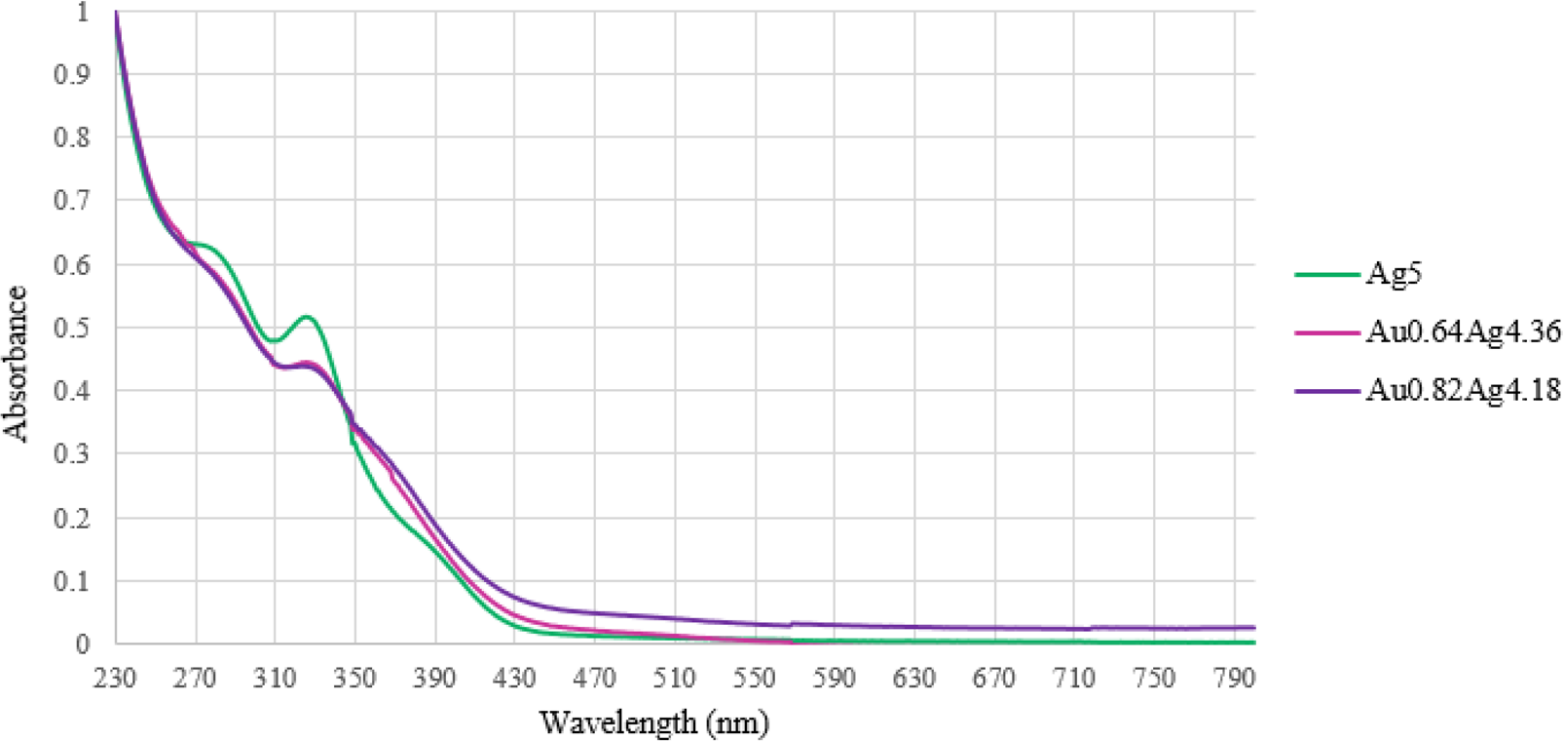
UV–visible absorption spectra of [NEt_4_]_3_[Au_*x*_Ag_5–*x*_Fe_4_(CO)_16_] in CH_3_CN at 298 K (concentration 1.25 × 10^–5^ M). Ag5 = [Ag_5_Fe_4_(CO)_16_]^3–^; Au0.64Ag4.36 = [Au_0.64_Ag_4.36_Fe_4_(CO)_16_]^3–^; Au0.82Ag4.18 = [Au_0.82_Ag_4.18_Fe_4_(CO)_16_]^3–^.

[M_*x*_M′_5–*x*_Fe_4_(CO)_16_]^3–^ clusters have been studied by means of UV–visible spectroscopy in CH_3_CN solution. The complete spectra can be found in Figures S38–S54 in the Supporting Information, whereas cumulative spectra are reported in Figures 4–6. The spectra of the binary [M_5_Fe_4_(CO)_16_]^3–^ clusters have been recorded as references. A list of the main absorption bands of all of the [M_*x*_M′_5–*x*_Fe_4_(CO)_16_]^3–^ clusters with extinction coefficients are reported in Tables S11–S13.

[Cu_5_Fe_4_(CO)_16_]^3–^ displays three weak features at 266, 331, and 408 nm; that at 331 nm is very weak. [Ag_5_Fe_4_(CO)_16_]^3–^ displays a strong absorption at 326 nm accompanied by two weak shoulders at 283 and 399 nm. The strong feature at 326 nm seems to be predictive of the presence of Ag in the cluster (see below). [Au_5_Fe_4_(CO)_16_]^3–^ is readily oxidized after the dilution necessary for UV–vis spectroscopy, and therefore, its spectrum has not been recorded.

In the case of ternary [Au_*x*_Cu_5–*x*_Fe_4_(CO)_16_]^3–^ clusters (Table S11, Figure 4), the UV–visible spectra show only weak features. As a general trend, the weak absorption at ca. 269 nm increases by increasing the Cu content, whereas that at 347–367 nm increases by increasing the Au content. A weak absorption at 428–435 nm appears when significant amounts of Cu are present (compositions from Cu_3.91_Au_1.09_ to Cu_2.52_Au_2.48_), whereas it is absent in the case of Cu_0.38_Au_4.62_. It must be remarked that all of these features are very weak and, therefore, sometimes it is not easy to clearly detect them.

In the case of [Ag_*x*_Cu’_5–*x*_Fe_4_(CO)_16_]^3–^ clusters (Table S12, Figure 5), there is a strong absorption at 326–337 nm whose intensity decreases by decreasing the Ag content, accompanied by two weaker features at lower and higher wavelengths. The strong feature at 326–337 nm is indicative of the presence of Ag in the clusters, as also indicated by the UV–visible spectra of [Au_*x*_Ag_5–*x*_Fe_4_(CO)_16_]^3–^ clusters (Table S13, Figure 6), whereas it is completely absent in those of [Au_*x*_Cu_5–*x*_Fe_4_(CO)_16_]^3–^.

### 2.5 DFT Investigation on [M_*x*_M′_5–*x*_Fe_4_(CO)_16_]^3–^ Clusters (M, M′ = Cu, Ag, Au; M ≠ M′; *x* = 0–5)

**Scheme 6:**
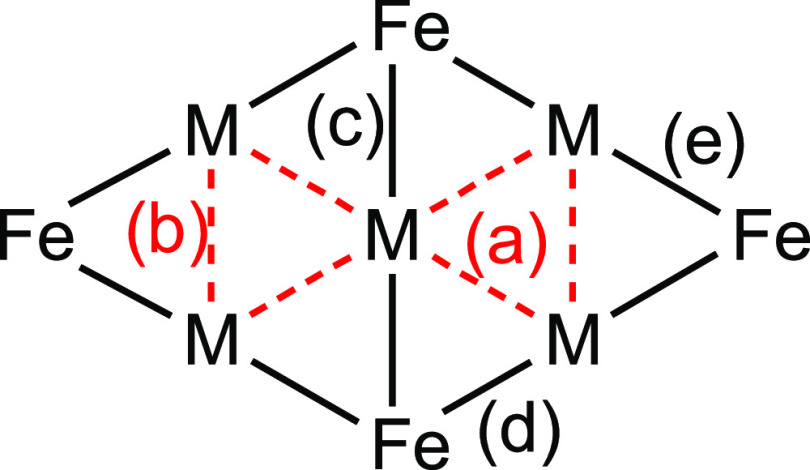
General Representation of the M–M and M–Fe Bonds Obtained from the Analysis of the (3, –1) Bond Critical Points Types of interactions: (a) M_centre_–M_corner_; (b) M_corner_–M_corner_; (c) M_centre_–Fe; (d and e) M_corner_–Fe.

**Scheme 7:**
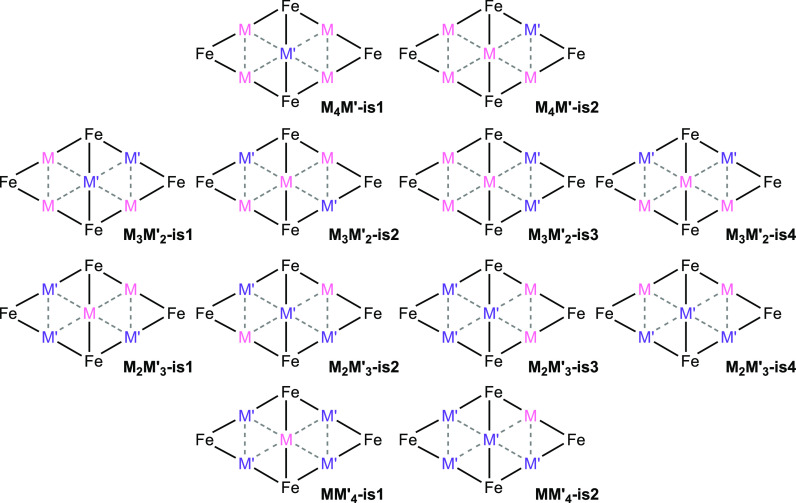
Isomers of the [M_*x*_M′_5–*x*_Fe_4_(CO)_16_]^3–^ Clusters with Acronyms Only the metal centers are sketched for clarity.

The general representation of the M–M and M–Fe bonds obtained from the analysis of the (3, –1) bond critical points (b.c.p.) is depicted in Scheme 6. Selected computed data at (3, –1) b.c.p. (electron density, ρ; potential energy density, *V*; energy density, *E*; Laplacian of electron density, ∇^2^ρ) for all of the possible isomers of [M_*x*_M′_5–*x*_Fe_4_(CO)_16_]^3–^ clusters are collected in the Supporting Information (Tables S14–S25). The isomers are sketched with their acronyms in Scheme 7 for clarity. The small negative values of *E* and the positive values of ∇^2^ρ at b.c.p. agree in all of the cases with Bianchi’s definition of metal–metal bonds.^50^

For what concerns the M–M interactions, the following trends are observed:

(1) On considering type (a) interactions (M_center_–M_corner_), (3, –1) b.c.p. for Cu–Cu bonds are observed only without a Cu–Ag or Cu–Au bond at the same side of the molecule. (3, –1) b.c.p. for bonds involving other couples of attractors are always detected. On considering different Cu–Ag, Cu–Au, or Ag–Ag (3, –1) b.c.p. in the same molecule, the electron density (ρ) is lower (and the potential energy density *V* is less negative) when the other M–M bond at the same side of the molecule involves heavier atoms. Ag–Au and Au–Au b.c.p. properties are less affected by the adjacent bonds.
(2) For what concerns type (b) interactions (M_corner_–M_corner_), (3, –1) b.c.p. for Cu–Cu and Cu–Ag bonds are never observed. Cu–Au, Ag–Ag, or Au–Au (3, –1) b.c.p. are detected only when the central metal is Cu. In all of the other cases, no (3, –1) b.c.p. is localized.

These data suggest a competition in the localization of electron density among type (a) and (b) bonds. The Cu–Cu bonds appear the least favorable, and type (a) (3, –1) b.c.p. were detected only in the absence of Cu–Ag or Cu–Au bonds close to Cu–Cu. Moreover, Cu as a central metal favors the localization of type (b) (3, –1) b.c.p., that are absent in the other cases. This last condition is however nonsufficient to localize (3, –1) Cu–Ag b.c.p.

The M–Fe bonds show lower variability, since 10 (3, –1) Fe–M b.c.p. are localized for all of the considered isomers. The general trends observed are the following:

(1) M–Fe interactions with the same M and with Fe atoms bonded to the same coinage metals follow the strength order (e) > (d) > (c), as indicated by ρ and *V* values at (3, –1) b.c.p. This is in agreement with the fact that in (e) a Fe(CO)_4_ group is bonded to two M sites, whereas in (c) and (d) the Fe(CO)_4_ group is bonded to three M sites.
(2) On comparing bonds of the same type, the M–Fe density values at (3, –1) b.c.p. are slightly lowered if Fe is involved in another interaction with a heavier atom, with this suggesting the preferential localization of electron density on Fe–Ag and Fe–Au bonds.

**Figure 7:**
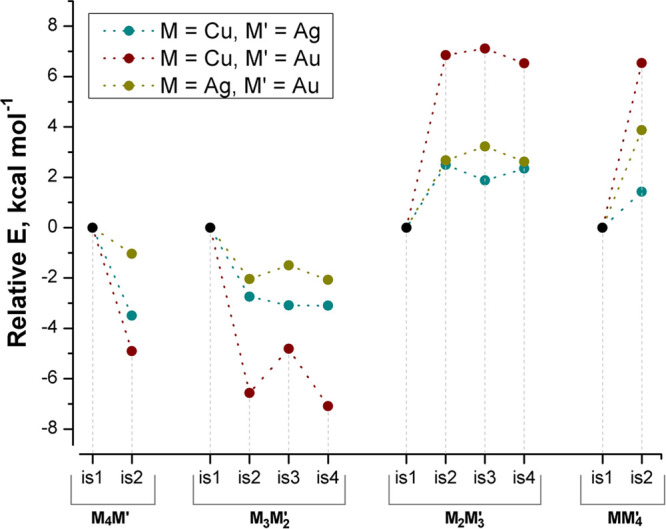
Relative energy values for the isomers of [M_*x*_M′_5–*x*_Fe_4_(CO)_16_]^3–^ clusters. Dotted lines are drawn for clarity purposes.

The comparison of the relative energy values among the isomers, reported in Figure 7, allows one to qualitatively recognize the dominant parameters determining the thermodynamic stability. In the case of [Cu_*x*_Ag_5–*x*_Fe_4_(CO)_16_]^3–^ and [Cu_*x*_Au_5–*x*_Fe_4_(CO)_16_]^3–^ clusters, the most stable species are those where the number of type (e) and (d) Fe–Ag or Fe–Au bonds is maximized. The energy separations are more pronounced among the Au-containing species, probably because the energy difference between Fe–Cu and Fe–Au bonds is greater than that between Fe–Cu and Fe–Ag. The same behavior is observed for the isomers of [Ag_*x*_Au_5–*x*_Fe_4_(CO)_16_]^3–^, where the clusters containing the highest numbers of Fe–Au bonds have the lowest relative energy. In agreement with the previous observation, the energy separations are lower than those computed for the [Cu_*x*_Au_5–*x*_Fe_4_(CO)_16_]^3–^ clusters.

The determination of the most stable species is doubtful only for [M_3_M′_2_Fe_4_(CO)_16_]^3–^ clusters, where three isomers (**M**_**3**_**M′**_**2**_**-is2**, **M**_**3**_**M′**_**2**_**-is3**, and **M**_**3**_**M′**_**2**_**-is4**) have roughly comparable energies, as expected because of the comparable Fe–M bond structure. Only in the case of [Cu_3_Au_2_Fe_4_(CO)_16_]^3–^, the **Cu**_**3**_**Au**_**2**_**-is3** isomer is appreciably less stable than **Cu**_**3**_**Au**_**2**_**-is2** and **Cu**_**3**_**Au**_**2**_**-is4**.

To summarize, it appears that M–M and Fe–M_center_ interactions (**a**–**c**) play a role of secondary importance in determining the stability of the compounds with respect to the Fe–M_corner_ bonds (**d** and **e**). The nature of M in these last interactions is a key parameter, because Fe–M bonds with heavier group 11 atoms, gold in particular, are preferred.

## 3 Conclusions

The synthesis of several 2-D molecular alloy clusters of general formula [M_*x*_M′_5–*x*_Fe_4_(CO)_16_]^3–^ (M, M′ = Cu, Ag, Au; M ≠ M′; *x* = 0–5) has been reported. These have been fully characterized by means of ESI-MS, IR, and UV–visible spectroscopies and their total structures determined through SC-XRD. Substitutional and compositional disorder are present in the solid state structures of these alloy molecular clusters. The latter refers to the fact that [M_*x*_M′_5–*x*_Fe_4_(CO)_16_]^3–^ are actually mixtures of clusters with slightly different M/M′ composition. Conversely, substitutional disorder indicates that also mixtures of clusters with the same composition but different distributions of M and M′ (isomers) are present. ESI-MS analyses point out that also in solution mixtures of isostructural clusters differing by a few M/M′ coinage metals are present. SC-XRD studies indicate a preferential distribution of Cu, Ag, and Au on the central and corner sites of the clusters. Thus, Au prefers to occupy the corner sites, whereas Cu displays a pronounced preference for the central site. Ag is somehow in the middle, and it is found preferentially in the central site in combination with Au and the corner sites in Ag–Cu clusters. This SC-XRD experimental evidence has been further corroborated by means of DFT studies. The site preference of the different coinage metals is mainly dictated by the strength of the Fe–M_corner_ bonds that decreases in the order Fe–Au > Fe–Ag > Fe–Cu. The preferred structure is the one that maximizes the number of stronger Fe–M interactions. Thus, Au always prefers corner sites, Cu the central site, whereas Ag prefers corner sites in combination with Cu and the central site in combination with Au. Shared-shell and metallophilic M–M interactions have lower weight in the determination of the most stable structures.

Overall, organometallic fragments such as Fe(CO)_4_ can be used in order to stabilize 2-D molecular alloy clusters. In turn, their molecular nature allows their structures to be fully revealed with atomic detail, resulting in the elucidation of the bonding parameters that determine the distribution of the different metals within their metal cores. This corroborates the idea that molecular clusters and nanoclusters may help our understanding of metal nanoparticles, metal aggregates, and surfaces.^1−6,14−27,51^

## 4 Experimental Section

### 4.1 General Experimental Procedures

All reactions and sample manipulations were carried out using standard Schlenk techniques under nitrogen and in dried solvents. All of the reagents were commercial products (Aldrich) of the highest purity available and used as received, except for [NEt_4_]_3_[Cu_3_Fe_3_(CO)_12_],^32^ [NEt_4_]_3_[Cu_5_Fe_4_(CO)_16_],^32^ [NEt_4_]_4_[Ag_4_Fe_4_(CO)_16_],^34^ and [NEt_4_]_3_[Ag_5_Fe_4_(CO)_16_],^34^ which were prepared according to the literature. Analyses of C, H, and N were obtained with a Thermo Quest Flash EA 1112NC instrument. Analyses of Fe, Cu, Ag, and Au were performed by microwave plasma-atomic emission spectrometry on an Agilent 4210 MP-AES instrument. IR spectra were recorded on a PerkinElmer Spectrum One interferometer in CaF_2_ cells. ESI mass spectra were recorded on a Waters Micromass ZQ4000 instrument using CH_3_CN as solvent (source temperature = 150 °C; capillary voltage = 2.54 kV; infusion flow = 20 μL/min; cone voltage = 10 V). Absorption spectra were recorded at 298 K using an Agilent Cary 100 UV–vis spectrometer. Structure drawings have been performed with SCHAKAL99.^52^

### 4.2 Synthesis of [Et_4_N]_3_[Ag_*x*_Cu_5–*x*_Fe_4_(CO)_16_] from [NEt_4_]_3_[Cu_3_Fe_3_(CO)_12_] and AgNO_3_

A variable volume (*V*_AgNO_3__, see list below) of a solution of AgNO_3_ (0.230 g, 1.36 mmol) in CH_3_CN (10 mL) was added dropwise to a solution of [NEt_4_]_3_[Cu_3_Fe_3_(CO)_12_] (0.468 g, 0.432 mmol) in CH_3_CN (15 mL). The mixture was stirred for 1 h at room temperature, and then, the solvent was removed under reduced pressure. The residue was washed with H_2_O (2 × 15 mL) and toluene (2 × 15 mL) and extracted with solvents of increasing polarity (acetone, CH_3_CN, dmf). The resulting solutions were analyzed by IR spectroscopy and, eventually, layered with an appropriate solvent (acetone/*n*-hexane; CH_3_CN/*n*-hexane/di-iso-propyl-ether; dmf/isopropanol) in the attempt to obtain crystals suitable for X-ray diffraction. The results were the following:

#### *V*_AgNO_3__ = 2.55 mL (0.344 mmol)

Crystals of [Et_4_N]_3_[Ag_1.02_Cu_3.98_Fe_4_(CO)_16_] were obtained by slow diffusion of isopropanol on the dmf fraction (yield 0.150 g, 31% based on Ag, 32% based on Cu).

C_40_H_60_Ag_1.02_Cu_3.98_Fe_4_N_3_O_16_ (1425.44): calcd (%): C 33.71, H 4.24, N 2.95, Fe 15.67, Cu 17.75, Ag 7.72; found: C 33.92, H 4.38, N 2.76, Fe 15.86, Cu 17.59, Ag 7.47. IR (nujol, 293 K) ν_CO_: 1943(s), 1905(m), 1854(s), 1829(sh) cm^–1^. IR (acetone, 293 K) ν_CO_: 1939(s), 1919(sh), 1897(m), 1845(s) cm^–1^. IR (CH_3_CN, 293 K) ν_CO_: 1943(s), 1922(sh), 1878(m), 1824(sh) cm^–1^.

#### *V*_AgNO_3__ = 4.13 mL (0.562 mmol)

Crystals of [Et_4_N]_3_[Ag_4.25_Cu_0.75_Fe_4_(CO)_16_] were obtained by slow diffusion of isopropanol on the dmf fraction (yield 0.074 g, 36% based on Ag, 3% based on Cu).

C_40_H_60_Ag_4.25_Cu_0.75_Fe_4_N_3_O_16_ (1568.63): calcd (%): C 30.63, H 3.86, N 2.68, Fe 14.24, Cu 3.04, Ag 29.23; found: C 30.41, H 4.04, N 2.39, Fe 13.07, Cu 2.91, Ag 29.44. IR (nujol, 293 K) ν_CO_: 1943(s), 1897(m), 1867(sh), 1851(s) cm^–1^. IR (CH_3_CN, 293 K) ν_CO_: 1980(sh), 1965(ms), 1950(s), 1895(sh), 1879(vs), 1835(w) cm^–1^.

#### *V*_AgNO_3__ = 6.67 mL (0.907 mmol)

Crystals of [Et_4_N]_3_[Ag_4.88_Cu_0.12_Fe_4_(CO)_16_] were obtained by slow diffusion of *n*-hexane and di-iso-propyl-ether on the CH_3_CN fraction (yield 0.081 g, 27% based on Ag, 0.5% based on Cu).

C_40_H_60_Ag_4.88_Cu_0.12_Fe_4_N_3_O_16_ (1596.34): calcd (%): C 30.10, H 3.79, N 2.63, Fe 13.99, Cu 0.48, Ag 32.98; found: C 30.38, H 3.59, N 2.97, Fe 14.12, Cu 0.69, Ag 32.75. IR (nujol, 293 K) ν_CO_: 1944(s), 1892(m), 1869(sh), 1853(s) cm^–1^. IR (CH_3_CN, 293 K) ν_CO_: 1947(s), 1882(m) cm^–1^.

#### *V*_AgNO_3__ = 7.31 mL (0.994 mmol)

Crystals of [Et_4_N]_3_[Ag_4.92_Cu_0.08_Fe_4_(CO)_16_] were obtained by slow diffusion of isopropanol on the dmf fraction (yield 0.095 g, 29% based on Ag, 0.4% based on Cu).

C_40_H_60_Ag_4.92_Cu_0.08_Fe_4_N_3_O_16_ (1598.11): calcd (%): C 30.06, H 3.78, N 2.63, Fe 13.98, Cu 0.32, Ag 33.21; found: C 29.86, H 4.05, N 2.32, Fe 13.78, Cu 0.21, Ag 33.02. IR (nujol, 293 K) ν_CO_: 1947(s), 1891(s) cm^–1^. IR (dmf, 293 K) ν_CO_: 1942(s), 1878(s) cm^–1^.

### 4.3 Synthesis of [Et_4_N]_3_[Ag_*x*_Cu_5–*x*_Fe_4_(CO)_16_] from [NEt_4_]_3_[Cu_5_Fe_4_(CO)_16_] and AgNO_3_

A solution of AgNO_3_ (0.144 g, 0.850 mmol) in CH_3_CN (5 mL) was added dropwise to a solution of [NEt_4_]_3_[Cu_5_Fe_4_(CO)_16_] (0.468 g, 0.340 mmol) in acetone (15 mL). The mixture was stirred for 1 h at room temperature, and then, the solvent was removed under reduced pressure. The residue was washed with H_2_O (2 × 15 mL) and toluene (2 × 15 mL) and extracted with solvents of increasing polarity (acetone, CH_3_CN, dmf). The resulting solutions were analyzed by IR spectroscopy and, eventually, layered with an appropriate solvent (acetone/*n*-hexane; CH_3_CN/*n*-hexane/di-iso-propyl-ether; dmf/isopropanol) in the attempt to obtain crystals suitable for X-ray diffraction.

Crystals of [Et_4_N]_3_[Ag_5_Fe_4_(CO)_16_] were obtained by slow diffusion of *n*-hexane and di-iso-propyl-ether on the CH_3_CN fraction (yield 0.038 g, 14% based on Ag).

C_40_H_60_Ag_5_Cu_0_Fe_4_N_3_O_16_ (1601.66): calcd (%): C 30.00, H 3.78, N 2.62, Fe 13.95, Ag 33.67; found: C 30.31, H 3.51, N 2.99, C 30.00, H 3.78, N 2.62, Fe 14.12, Ag 33.49. IR (nujol, 293 K) ν_CO_: 1944(m), 1890(sh), 1867(sh), 1853(s) cm^–1^. IR (CH_3_CN, 293 K) ν_CO_: 1949(s), 1894(sh), 1878(s), 1833(w) cm^–1^. IR (dmso, 293 K) ν_CO_: 1944(s), 1895(sh), 1872(vs), 1833(m) cm^–1^.

Crystals of [Et_4_N]_3_[Ag_4.81_Cu_0.20_Fe_4_(CO)_16_] were obtained by slow diffusion of isopropanol on the dmf fraction (yield 0.44 g, 16% based on Ag, 0.3% based on Cu).

C_40_H_60_Ag_4.81_Cu_0.20_Fe_4_N_3_O_163_ (1593.01): calcd (%): C 30.14, H 3.79, N 2.63, Fe 14.02, Cu 0.80, Ag 32.55; found: C 29.95, H 3.61, N 2.82, Fe 14.25, Cu 0.67, Ag 32.74. IR (dmf, 293 K) ν_CO_: 1945(vs), 1844(s) cm^–1^.

### 4.4 Synthesis of [Et_4_N]_3_[Ag_4.37_Cu_0.63_Fe_4_(CO)_16_] from [NEt_4_]_4_[Ag_4_Fe_4_(CO)_16_] and [Cu(CH_3_CN)_4_][BF_4_]

[Cu(CH_3_CN)_4_][BF_4_] (0.095 g, 0.306 mmol) was added as a solid in small portions to a solution of [NEt_4_]_4_[Ag_4_Fe_4_(CO)_16_] (0.468 g, 0.289 mmol) in CH_3_CN (15 mL). The mixture was stirred for 1 h at room temperature, and then, the solvent was removed under reduced pressure. The residue was washed with H_2_O (2 × 15 mL) and toluene (2 × 15 mL) and extracted with CH_3_CN (15 mL). Crystals of [Et_4_N]_3_[Ag_4.37_Cu_0.63_Fe_4_(CO)_16_] were obtained by slow diffusion of *n*-hexane and di-iso-propyl-ether on the CH_3_CN fraction (yield 0.121 g, 29% based on Ag, 16% based on Cu).

C_40_H_60_Ag_4.37_Cu_0.63_Fe_4_N_3_O_16_ (1573.73): calcd (%): C 30.53, H 3.84, N 2.67, Fe 14.19, Cu 2.54, Ag 29.95; found: C 30.37, H 4.02, N 2.83, Fe 14.02, Cu 2.68, Ag 30.14. IR (CH_3_CN, 293 K) ν_CO_: 1948(s), 1894(sh), 1878(s), 1833(w) cm^–1^.

### 4.5 Synthesis of [Et_4_N]_3_[Ag_4.90_Cu_0.10_Fe_4_(CO)_16_] from [NEt_4_]_3_[Ag_5_Fe_4_(CO)_16_] and Cu(IMes)Cl

Cu(IMes)Cl (0.354 g, 0.880 mmol) was added as a solid in small portions to a solution of [NEt_4_]_3_[Ag_5_Fe_4_(CO)_16_] (0.468 g, 0.293 mmol) in CH_3_CN (15 mL). The mixture was stirred for 1 h at room temperature, and then, the solvent was removed under reduced pressure. The residue was washed with H_2_O (2 × 15 mL) and toluene (2 × 15 mL) and extracted with CH_3_CN (15 mL). Crystals of [Et_4_N]_3_[Ag_4.90_Cu_0.10_Fe_4_(CO)_16_] were obtained by slow diffusion of *n*-hexane and di-iso-propyl-ether on the CH_3_CN fraction (yield 0.160 g, 34% based on Ag, 1.1% based on Cu).

C_40_H_60_Ag_4.90_Cu_0.10_Fe_4_N_3_O_16_ (1597.22): calcd (%): C 30.08, H 3.79, N 2.63, Fe 13.99, Cu 0.40, Ag 33.09; found: C 29.88, H 4.03, N 2.85, Fe 14.15, Cu 0.54, Ag 32.89. IR (CH_3_CN, 293 K) ν_CO_: 1950(s), 1895(sh), 1879(s), 1834(m) cm^–1^.

### 4.6 Synthesis of [Et_4_N]_3_[Ag_*x*_Cu_5–*x*_Fe_4_(CO)_16_] from [NEt_4_]_3_[Ag_5_Fe_4_(CO)_16_] and [NEt_4_]_3_[Cu_3_Fe_3_(CO)_12_]

A solution of [NEt_4_]_3_[Cu_3_Fe_3_(CO)_12_] (0.317 g, 0.293 mmol) in CH_3_CN (15 mL) was added dropwise to a solution of [NEt_4_]_3_[Ag_5_Fe_4_(CO)_16_] (0.468 g, 0.293 mmol) in CH_3_CN (15 mL). The mixture was stirred for 1 h at room temperature, and then, the solvent was removed under reduced pressure. The residue was washed with H_2_O (2 × 15 mL) and toluene (2 × 15 mL) and extracted with solvents of increasing polarity (acetone, CH_3_CN, dmf). The resulting solutions were analyzed by IR spectroscopy and, eventually, layered with an appropriate solvent (acetone/*n*-hexane; CH_3_CN/*n*-hexane/di-iso-propyl-ether; dmf/isopropanol) in the attempt to obtain crystals suitable for X-ray diffraction.

Crystals of [Et_4_N]_3_[Ag_3.30_Cu_1.70_Fe_4_(CO)_16_] were obtained by slow diffusion of *n*-hexane on the acetone fraction (yield 0.320 g, 47% based on Ag, 41% based on Cu).

C_40_H_60_Ag_3.30_Cu_1.70_Fe_4_N_3_O_16_ (1526.30): calcd (%): C 31.48, H 3.96, N 2.75, Fe 14.64, Cu 7.08, Ag 23.32; found: C 31.32, H 4.12, N 2.96, Fe 14.48, Cu 6.87, Ag 23.57. IR (acetone, 293 K) ν_CO_: 1942(s), 1898(m) cm^–1^.

Crystals of [Et_4_N]_3_[Ag_3.45_Cu_1.55_Fe_4_(CO)_16_] were obtained by slow diffusion of *n*-hexane and di-iso-propyl-ether on the CH_3_CN fraction (yield 0.100 g, 15% based on Ag, 12% based on Cu).

C_40_H_60_Ag_3.45_Cu_1.55_Fe_4_N_3_O_16_ (1532.72): calcd (%): C 31.34, H 3.95, N 2.74, Fe 14.57, Cu 6.43, Ag 24.28; found: C 31.62, H 3.79, N 2.95, Fe 14.38, Cu 6.29, Ag 24.57. IR (CH_3_CN, 293 K) ν_CO_: 1946(m), 1894(sh), 1847(s) cm^–1^.

### 4.7 Synthesis of [Cu(dppe)_2_]_3_[Ag_13_Fe_8_(CO)_32_] from [NEt_4_]_3_[Cu_5_Fe_4_(CO)_16_] and Ag(dppe)(NO_3_)

Ag(dppe)(NO_3_) (0.482 g, 0.850 mmol) was added as a solid in small portions to a solution of [NEt_4_]_3_[Cu_5_Fe_4_(CO)_16_] (0.468 g, 0.340 mmol) in acetone (15 mL). The mixture was stirred for 1 h at room temperature, and then, the solvent was removed under reduced pressure. The residue was dissolved in dmf (10 mL), and further, Ag(dppe)(NO_3_) (0.482 g, 0.850 mmol) was added as a solid. The mixture was stirred for 1 h at room temperature, and then, a saturated solution of [NEt_4_]Br in H_2_O (40 mL) was added up to complete precipitation of the products. The solid was recovered by filtration, washed with H_2_O (2 × 15 mL), toluene (2 × 15 mL), thf (15 mL), acetone (15 mL), and CH_3_CN (15 mL) and eventually extracted with dmf (10 mL). Crystals of [Cu(dppe)_2_]_3_[Ag_13_Fe_8_(CO)_32_] were obtained by slow diffusion of isopropanol on the dmf solution (yield 0.104 g, 15% based on Ag, 3.4% based on Cu).

While repeating this reaction, a few crystals of Cu_3_Br_3_(dppe)_3_ were also obtained as a side product and mechanically separated from [Cu(dppe)_2_]_3_[Ag_13_Fe_8_(CO)_32_].

C_188_H_144_Ag_13_Cu_3_Fe_8_O_32_P_12_ (5323.39): calcd (%): C 42.29, H 2.72, Fe 8.39, Cu 3.58, Ag 26.33; found: C 42.67, H 2.35, Fe 8.53, Cu 3.29, Ag 26.47. IR (nujol, 293 K) ν_CO_: 1994(s), 1912(m) cm^–1^. IR (dmf, 293 K) ν_CO_: 1999(vs), 1916(ms) cm^–1^.

### 4.8 Synthesis of [Et_4_N]_3_[Au_*x*_Cu_5–*x*_Fe_4_(CO)_16_] from [NEt_4_]_3_[Cu_3_Fe_3_(CO)_12_] and Au(PPh_3_)Cl

A variable amount (*m*_Au(PPh3)Cl_, see list below) of Au(PPh_3_)Cl was added as a solid to a solution of [NEt_4_]_3_[Cu_3_Fe_3_(CO)_12_] (0.468 g, 0.432 mmol) in CH_3_CN (15 mL). The mixture was stirred for 1 h at room temperature, and then, the solvent was removed under reduced pressure. The residue was washed with H_2_O (2 × 15 mL) and toluene (2 × 15 mL) and extracted with solvents of increasing polarity (acetone, CH_3_CN, dmf). The resulting solutions were analyzed by IR spectroscopy and, eventually, layered with an appropriate solvent (acetone/*n*-hexane; CH_3_CN/*n*-hexane/di-iso-propyl-ether; dmf/isopropanol) in the attempt to obtain crystals suitable for X-ray diffraction. The results were the following:

#### *m*_Au(PPh_3_)Cl_ = 0.284 g (0.605 mmol)

Crystals of [Et_4_N]_3_[Au_1.15_Cu_3.85_Fe_4_(CO)_16_] were obtained by slow diffusion of *n*-hexane on the acetone fraction (yield 0.160 g, 20% based on Ag, 31% based on Cu).

C_40_H_60_Au_1.15_Cu_3.85_Fe_4_N_3_O_16_ (1534.11): calcd (%): C 31.33, H 3.94, N 2.74, Fe 14.57, Cu 15.95, Au 14.77; found: C 31.08, H 4.13, N 2.53, Fe 14.29, Cu 16.11, Au 14.89. IR (acetone, 293 K) ν_CO_: 1946(s), 1884(s), 1831(w) cm^–1^. IR (dmf, 293 K) ν_CO_: 1946(s), 1881(s).

#### *m*_Au(PPh_3_)Cl_ = 0.305 g (0.648 mmol)

Crystals of [Et_4_N]_3_[Au_1.31_Cu_3.69_Fe_4_(CO)_16_] were obtained by slow diffusion of *n*-hexane on the acetone fraction (yield 0.168 g, 22% based on Au, 31% based on Cu).

C_40_H_60_Au_1.31_Cu_3.69_Fe_4_N_3_O_16_ (1555.46): calcd (%): C 30.90, H 3.89, N 2.70, Fe 14.37, Cu 15.08, Au 16.60; found: C 30.77, H 4.04, N 2.56, Fe 14.19, Cu 14.87, Au 16.91. IR (acetone, 293 K) ν_CO_: 1947(s), 1882(m) cm^–1^.

Crystals of [Et_4_N]_3_[Au_1.67_Cu_3.33_Fe_4_(CO)_16_] were obtained by slow diffusion of isopropanol on the dmf fraction (yield 0.112 g, 18% based on Au, 18% based on Cu).

C_40_H_60_Au_1.67_Cu_3.33_Fe_4_N_3_O_16_ (1602.83): calcd (%): C 29.97, H 3.77, N 2.62, Fe 13.94, Cu 13.20, Au 20.52; found: C 30.14, H 4.02, N 2.36, Fe 13.81, Cu 13.49, Au 20.21. IR (dmf, 293 K) ν_CO_: 1945(s), 1878(m) cm^–1^.

#### *m*_Au(PPh_3_)Cl_ = 0.610 g (1.30 mmol)

Crystals of [Et_4_N]_3_[Au_2.48_Cu_2.52_Fe_4_(CO)_16_] were obtained by slow diffusion of isopropanol on the dmf fraction (yield 0.130 g, 14% based on Au, 15% based on Cu).

C_40_H_60_Au_2.48_Cu_2.52_Fe_4_N_3_O_16_ (1710.90): calcd (%): C 28.08, H 3.53, N 2.46, Fe 13.06, Cu 9.36, Au 28.55; found: C 28.27, H 3.31, N 2.62, Fe 13.21, Cu 9.08, Au 28.27. IR (dmf, 293 K) ν_CO_: 1951(2), 1887(2) cm^–1^.

### 4.9 Synthesis of [Et_4_N]_3_[Au_*x*_Cu_5–*x*_Fe_4_(CO)_16_] from [NEt_4_]_3_[Cu_3_Fe_3_(CO)_12_] and Au(Et_2_S)Cl

A variable volume (*V*_Au(Et_2_S)Cl_, see list below) of a solution of Au(Et_2_S)Cl (0.348 g, 1.08 mmol) in CH_3_CN (10 mL) was added dropwise to a solution of [NEt_4_]_3_[Cu_3_Fe_3_(CO)_12_] (0.468 g, 0.432 mmol) in CH_3_CN (15 mL). The mixture was stirred for 1 h at room temperature, and then, the solvent was removed under reduced pressure. The residue was washed with H_2_O (2 × 15 mL) and toluene (2 × 15 mL) and extracted with solvents of increasing polarity (acetone, CH_3_CN, dmf). The resulting solutions were analyzed by IR spectroscopy and, eventually, layered with an appropriate solvent (acetone/*n*-hexane; CH_3_CN/*n*-hexane/di-iso-propyl-ether; dmf/isopropanol) in the attempt to obtain crystals suitable for X-ray diffraction. The results were the following:

#### *V*_Au(Et_2_S)Cl_ = 2.80 mL (0.302 mmol)

Crystals of [Et_4_N]_3_[Au_2.18_Cu_2.83_Fe_4_(CO)_16_] and [Et_4_N]_3_[Au_2.73_Cu_2.28_Fe_4_(CO)_16_] were obtained by slow diffusion of *n*-hexane and di-iso-propyl-ether on the CH_3_CN fraction (yield 0.098 g, 47% based on Au, 11% based on Cu).

C_40_H_60_Au_2.73_Cu_2.28_Fe_4_N_3_O_16_ (1743.59): calcd (%): C 27.53, H 3.47, N 2.41, Fe 12.80, Cu 8.30, Au 30.82; C_40_H_60_Au_2.18_Cu_2.83_Fe_4_N_3_O_16_ (1670.21): calcd (%): C 28.74, H 3.62, N 2.52, Fe 13.36, Cu 10.76, Au 25.69; found (mixture): C 28.25, H 3.34, N 2.63, Fe 13.05, Cu 9.12, Au 28.55. IR (nujol, 293 K) ν_CO_: 1948(s), 1911(m), 1864(s) cm^–1^. IR (acetone, 293 K) ν_CO_: 1999(w), 1974(m), 1951(vs), 1922(s), 1864(s) cm^–1^. IR (CH_3_CN, 293 K) ν_CO_: 1950(s), 1915(s), 1864(s) cm^–1^.

#### *V*_Au(Et_2_S)Cl_ = 7.60 mL (0.821 mmol)

Crystals of [Et_4_N]_3_[Au_4.59_Cu_0.42_Fe_4_(CO)_16_] and [Et_4_N]_3_[Au_4.62_Cu_0.38_Fe_4_(CO)_16_] were obtained by slow diffusion of *n*-hexane and di-iso-propyl-ether on the CH_3_CN fraction (yield 0.104 g, 29% based on Au, 1.6% based on Cu).

C_40_H_60_Au_4.62_Cu_0.38_Fe_4_N_3_O_163_ (1995.77): calcd (%): C 24.09, H 3.03, N 2.11, Fe 11.20, Cu 1.21, Au 45.53; C_40_H_60_Au_4.59_Cu_0.42_Fe_4_N_3_O_163_ (1991.77): calcd (%): C 24.11, H 3.03, N 2.11, Fe 11.21, Cu 1.34, Au 45.36; found (mixture): C 24.21, H 2.86, N 2.29, Fe 11.34, Cu 1.16, Au 45.61. IR (nujol, 293 K) ν_CO_: 1954(s), 1869(s) cm^–1^. IR (acetone, 293 K) ν_CO_: 1956(s), 1930(ms), 1902(m) cm^–1^.

### 4.10 Synthesis of [Et_4_N]_3_[Au_1.09_Cu_3.91_Fe_4_(CO)_16_] from [NEt_4_]_3_[Cu_5_Fe_4_(CO)_16_] and Au(Et_2_S)Cl

A solution of Au(Et_2_S)Cl (0.131 g, 0.408 mmol) in acetone (5 mL) was added dropwise to a solution of [NEt_4_]_3_[Cu_5_Fe_4_(CO)_16_] (0.468 g, 0.340 mmol) in acetone (15 mL). The mixture was stirred for 1 h at room temperature, and then, the solvent was removed under reduced pressure. The residue was washed with H_2_O (2 × 15 mL) and toluene (2 × 15 mL) and extracted with acetone. Crystals of [Et_4_N]_3_[Au_1.09_Cu_3.91_Fe_4_(CO)_16_] were obtained by slow diffusion of *n*-hexane on the acetone fraction (yield 0.32 g, 56% based on Ag, 48% based on Cu).

C_40_H_60_Au_1.09_Cu_3.91_Fe_4_N_3_O_16_ (1525.44): calcd (%): C 31.49, H 3.96, N 2.75, Fe 14.64, Cu 16.29, Au 14.07; found: C 31.79, H 4.12, N 2.54, Fe 14.39, Cu 16.47, Au 13.87. IR (acetone, 293 K) ν_CO_: 1945(s), 1880(m) cm^–1^.

### 4.11 Synthesis of [Et_4_N]_3_[Au_*x*_Ag_5–*x*_Fe_4_(CO)_16_] from [NEt_4_]_4_[Ag_4_Fe_4_(CO)_16_] and Au(Et_2_S)Cl

A solution of Au(Et_2_S)Cl (0.075 g, 0.231 mmol) in CH_3_CN (4 mL) was added dropwise to a solution of [NEt_4_]_4_[Ag_4_Fe_4_(CO)_16_] (0.468 g, 0.289 mmol) in CH_3_CN (15 mL). The mixture was stirred for 1 h at room temperature, and then, the solvent was removed under reduced pressure. The residue was washed with H_2_O (2 × 15 mL) and toluene (2 × 15 mL) and extracted with solvents of increasing polarity (acetone, CH_3_CN, dmf). The resulting solutions were analyzed by IR spectroscopy and, eventually, layered with an appropriate solvent (acetone/*n*-hexane; CH_3_CN/*n*-hexane/di-iso-propyl-ether; dmf/isopropanol) in the attempt to obtain crystals suitable for X-ray diffraction.

Crystals of [Et_4_N]_3_[Au_0.81_Ag_4.20_Fe_4_(CO)_16_] were obtained by slow diffusion of *n*-hexane on the acetone fraction (yield 0.099 g, 21% based on Au, 21% based on Ag).

C_40_H_60_Au_0.81_Ag_4.20_Fe_4_N_3_O_16_ (1673.38): calcd (%): C 28.68, H 3.61, N 2.51, Fe 13.34, Ag 27.05, Au 9.53; found: C 29.03, H 3.39, N 2.24, Fe 13.49, Ag 26.85, Au 9.78. IR (acetone, 293 K) ν_CO_: 1946(vs), 1927(m), 1896(ms), 1874(s), 1835(sh) cm^–1^.

Crystals of [Et_4_N]_3_[Au_0.64_Ag_4.36_Fe_4_(CO)_16_] were obtained by slow diffusion of *n*-hexane and di-iso-propyl-ether on the CH_3_CN fraction (yield 0.180 g, 30% based on Au, 41% based on Ag).

C_40_H_60_Au_0.64_Ag_4.36_Fe_4_N_3_O_16_ (1658.68): calcd (%): C 28.96, H 3.65, N 2.53, Fe 13.47, Ag 28.35, Au 7.60; found: C 29.31, H 3.39, N 2.22, Fe 13.22, Ag 28.04, Au 7.85. IR (CH_3_CN, 293 K) ν_CO_: 1967(sh), 1950(s), 1930(sh), 1880(s), 1835(sh) cm^–1^.

### 4.12 X-ray Crystallographic Study

Crystal data and collection details for [NEt_4_]_3_[M_*x*_M′_5–*x*_Fe_4_(CO)_16_] (*x* = 0–5; M, M′ = Cu, Ag, Au; M ≠ M′) are reported in Tables S26–S29. The diffraction experiments were carried out on a Bruker APEX II diffractometer equipped with a PHOTON100 detector using Mo Kα radiation. Data were corrected for Lorentz polarization and absorption effects (empirical absorption correction SADABS).^53^ Structures were solved by direct methods and refined by full-matrix least-squares based on all data using *F*^2^.^54^ Hydrogen atoms were fixed at calculated positions and refined by a riding model. All non-hydrogen atoms were refined with anisotropic displacement parameters, unless otherwise stated. [NEt_4_]_3_[M_*x*_M′_5–*x*_Fe_4_(CO)_16_] (*x* = 0–5; M, M′ = Cu, Ag, Au; M ≠ M′) are isomorphous with [NEt_4_]_3_[M_5_Fe_4_(CO)_16_] (M = Cu, Ag, Au).^28,32,34^ The salts analyzed displayed some disorder of the CO ligands and/or [NEt_4_]^+^ cations. This, in some cases, lowered the symmetry of the space group from *P*4_2_/*mnm* to *P*4̅2_1_*m*. In the case of the lower symmetry *P*4̅2_1_*m* space group, the crystals were refined as racemic twins. The positions occupied by M(1) and M(2) are disordered Cu/Ag, Cu/Au, or Ag/Cu.

### 4.13 Computational Details

Geometry optimizations of the clusters were performed in the gas phase without symmetry constraints using the PBEh-3c method, a modified version of PBE0 (with 42% HF exchange) that uses a split-valence double-ζ basis set (def2-mSVP) and adds three corrections that consider dispersion, basis set superposition, and other basis set incompleteness effects.^55^ The “restricted” DFT approach was always applied. Calculations were performed with ORCA 4.2.0.^56^ The output, converted in .molden format, was elaborated with the software Multiwfn, version 3.5.^57^ Cartesian coordinates of all of the DFT-optimized structures have been included in the Supporting Information as an .xyz file.

## Supporting Information Available

Crystallographic data in CIF format, IR spectra, ESI-MS spectra, UV–visible absorption spectra, selected computed data for all of the isomers, and crystals and experimental details (PDF)
Optimized coordinates in XYZ format (XYZ)

## Supplementary Material

ic0c02443_si_001.pdf

ic0c02443_si_002.xyz

## Accession Codes

CCDC 2016042–2016064 contain the supplementary crystallographic data for this paper. These data can be obtained free of charge via www.ccdc.cam.ac.uk/data_request/cif, or by emailing data_request@ccdc.cam.ac.uk, or by contacting The Cambridge Crystallographic Data Centre, 12 Union Road, Cambridge CB2 1EZ, UK; fax: +44 1223 336033.
