# Supplementary material for: Synthesis, Structural Characterization, and DFT Investigations of [MxM′5–xFe4(CO)16]3– (M, M′ = Cu, Ag, Au; M ≠ M′) 2-D Molecular Alloy Clusters
Source: Inorg Chem. 2020 Oct 20;59(21):15936–52. doi: 10.1021/acs.inorgchem.0c02443 (PMC8015236; doi:10.1021/acs.inorgchem.0c02443)
Supplement: Supplementary file 1 — ic0c02443_si_001.pdf [file ic0c02443_si_001.pdf]

Supporting Information for

Synthesis, structural characterization and DFT investigations of $[M_xM'_{5-x}Fe_4(CO)_{16}]^{3-}$ (M, M' = Cu, Ag, Au; M ≠ M') 2-D molecular alloy clusters

Beatrice Berti,[a] Marco Bortoluzzi,[b] Cristiana Cesari,[a] Cristina Femoni,[a] Maria Carmela Iapalucci,[a] Leonardo Soleri,[a] and Stefano Zacchini*,[a]

[a] Dipartimento di Chimica Industriale "Toso Montanari", University of Bologna, Viale Risorgimento 4, I-40136 Bologna Italy. E-mail: stefano.zacchini@unibo.it; Web: https://www.unibo.it/sitoweb/stefano.zacchini/en; Tel: +39 051 2093711.

[b] Dipartimento di Scienze Molecolari e Nanosistemi, Ca' Foscari University of Venice, Via Torino 155 – 30175 Mestre (Ve), Italy.

	Page/s
IR spectra	S2-S10
M-M and M-Fe distances	S11-S12
ESI-MS spectra with peaks assignment	S13-S40
UV-visible absorption spectra	S41-S51
Selected computed data for all the isomers	S52-S63
X-Ray crystallographic data	S64-S69

Figure S1

IR spectra (ν_{CO} region) recorded in CH_3CN of (a) $[Cu_3Fe_3(CO)_{12}]^{3-}$, (b) $[Cu_5Fe_4(CO)_{16}]^{3-}$ and (c) $[Ag_4Fe_4(CO)_{16}]^{4-}$. Spectral resolution 2 cm^{-1}.

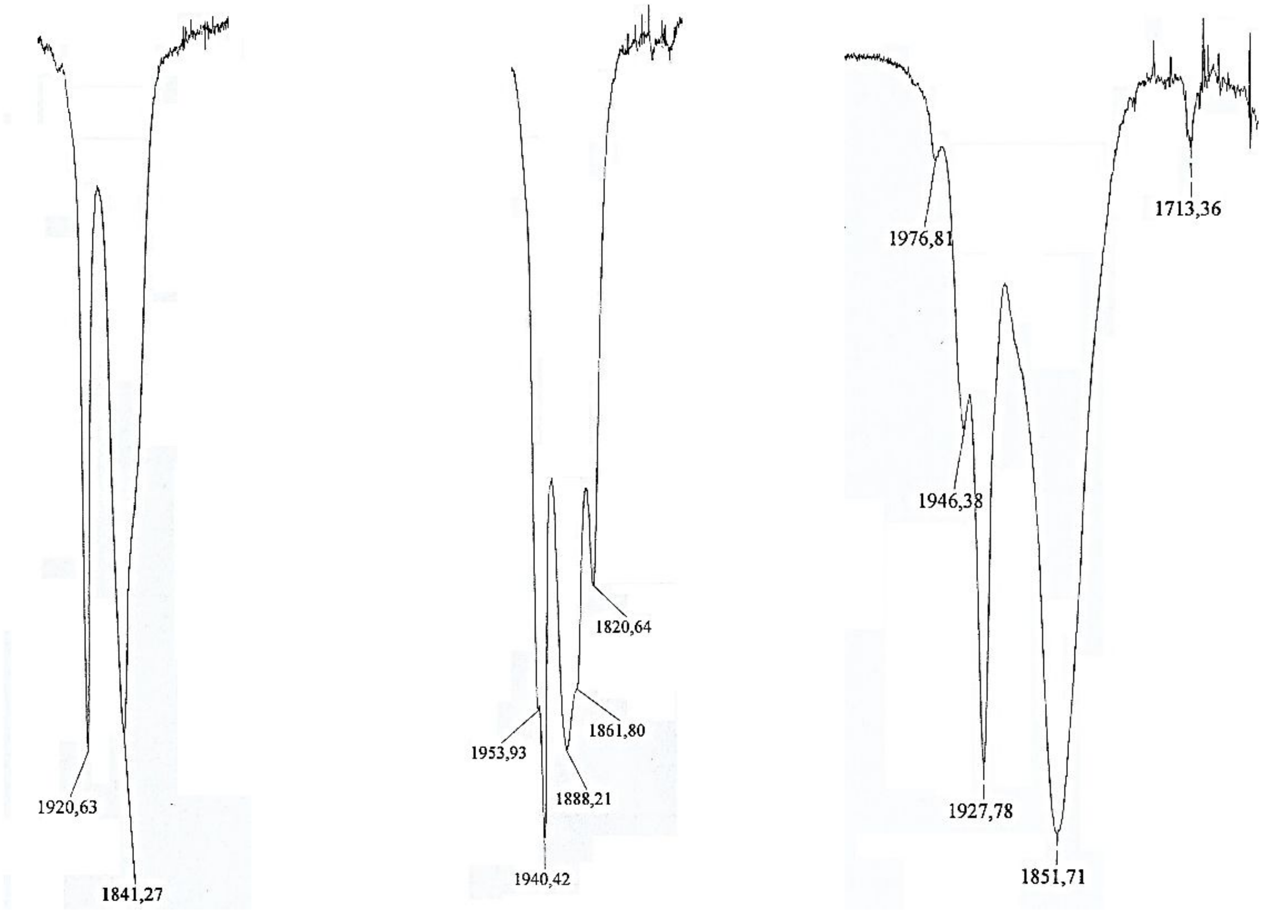

(a) (b) (c)

Figure S2

IR spectra (ν_{CO} region) recorded in CH_3CN of (a) $[Ag_5Fe_4(CO)_{16}]^{3-}$, (b) $[Ag_6Fe_4(CO)_{16}]^{2-}$, and (c) $[Ag_{13}Fe_8(CO)_{32}]^{3-}$. Spectral resolution 2 cm^{-1}.

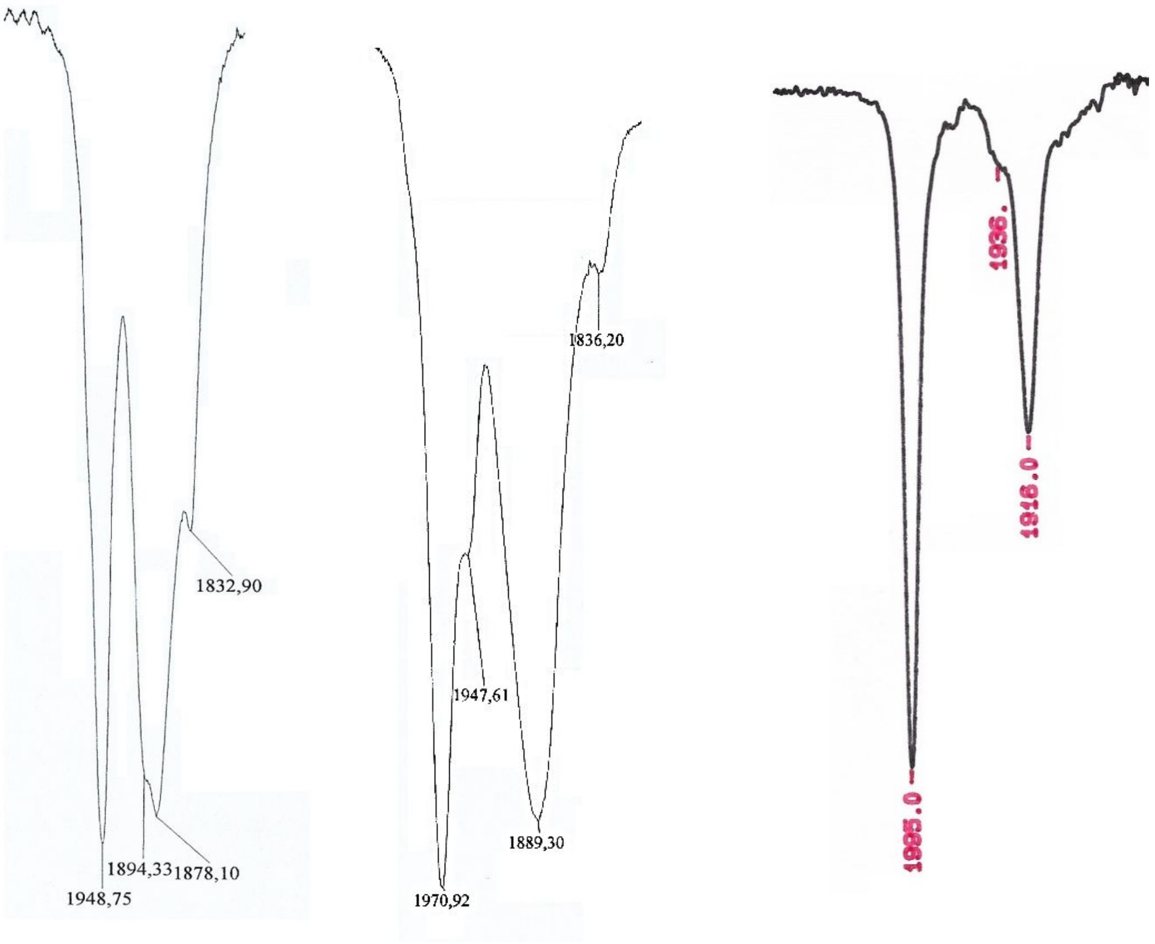

(a) (b) (c)

Figure S3

IR spectra (ν_{CO} region) recorded in CH_3CN of (a) $[AuFe_4(CO)_{16}]^-$, (b) $[Au_6Fe_4(CO)_{16}]^{2-}$ and (c) $[Au_5Fe_4(CO)_{16}]^{2-}$. Spectral resolution 2 cm^{-1}.

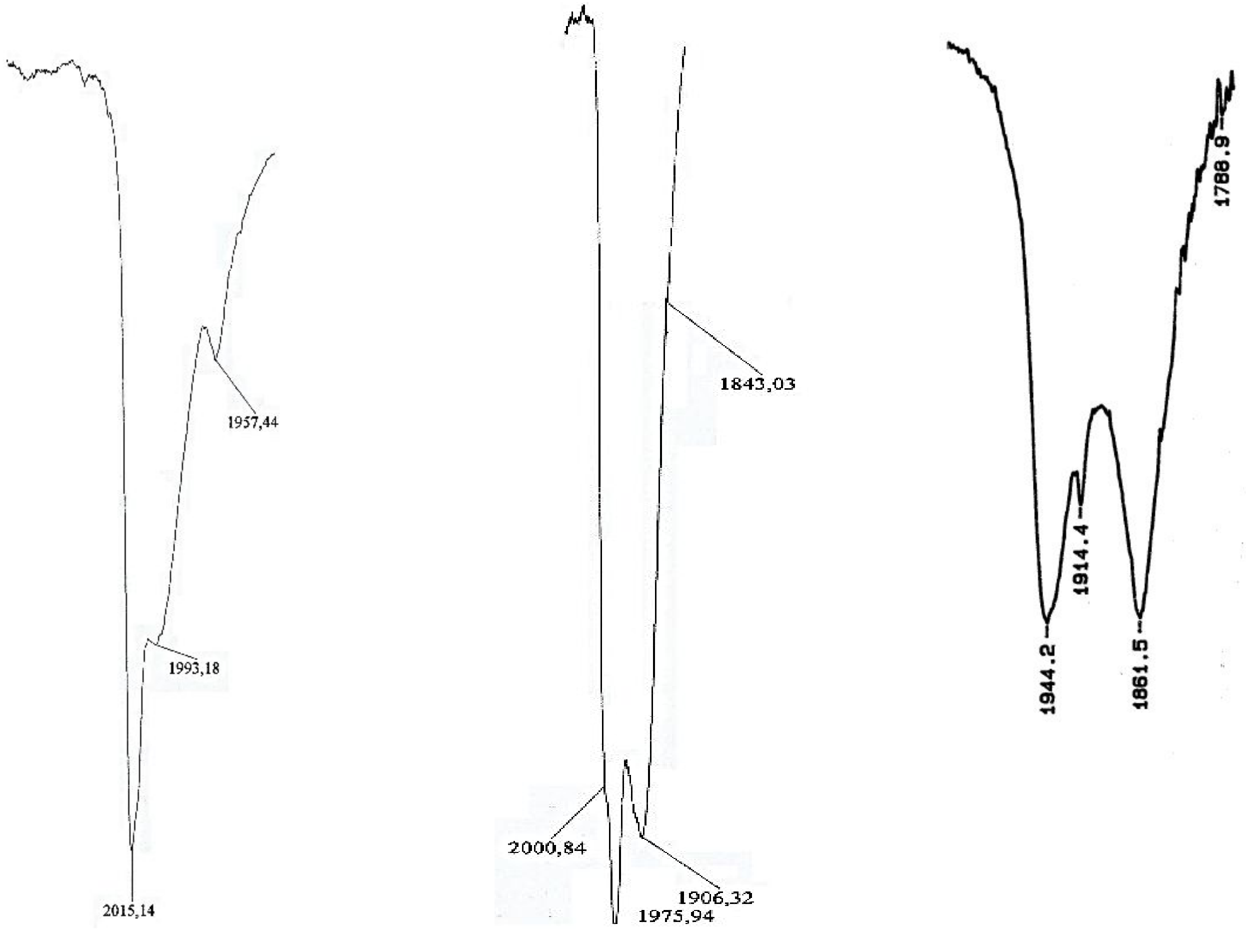

(a) (b) (c)

Figure S4

IR spectra (ν_{CO} region) recorded in CH_3CN of (a) $[NEt_4]_3[Ag_{1.02}Cu_{3.98}Fe_4(CO)_{16}]$ and (b) $[NEt_4]_3[Ag_{4.25}Cu_{0.75}Fe_4(CO)_{16}]$ (the band at 1964.98 cm^{-1} is due to impurities). Spectral resolution 2 cm^{-1}.

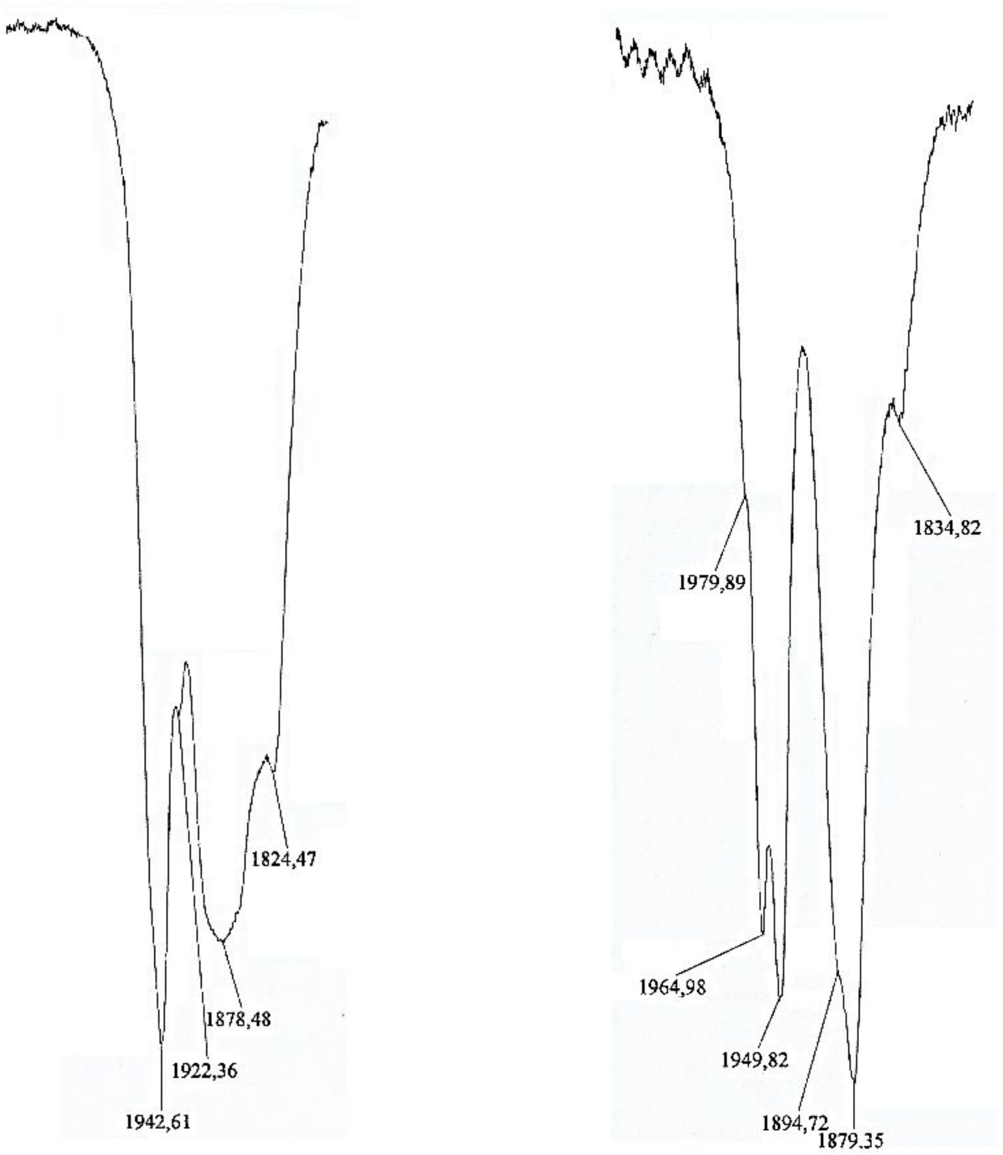

(a) (b)

Figure S5

IR spectra (ν_{CO} region) recorded in CH_3CN of (a) $[NEt_4]_3[Ag_{4.37}Cu_{0.63}Fe_4(CO)_{16}]$ and (b) $[NEt_4]_3[Ag_5Cu_0Fe_4(CO)_{16}]$. Spectral resolution 2 cm^{-1}.

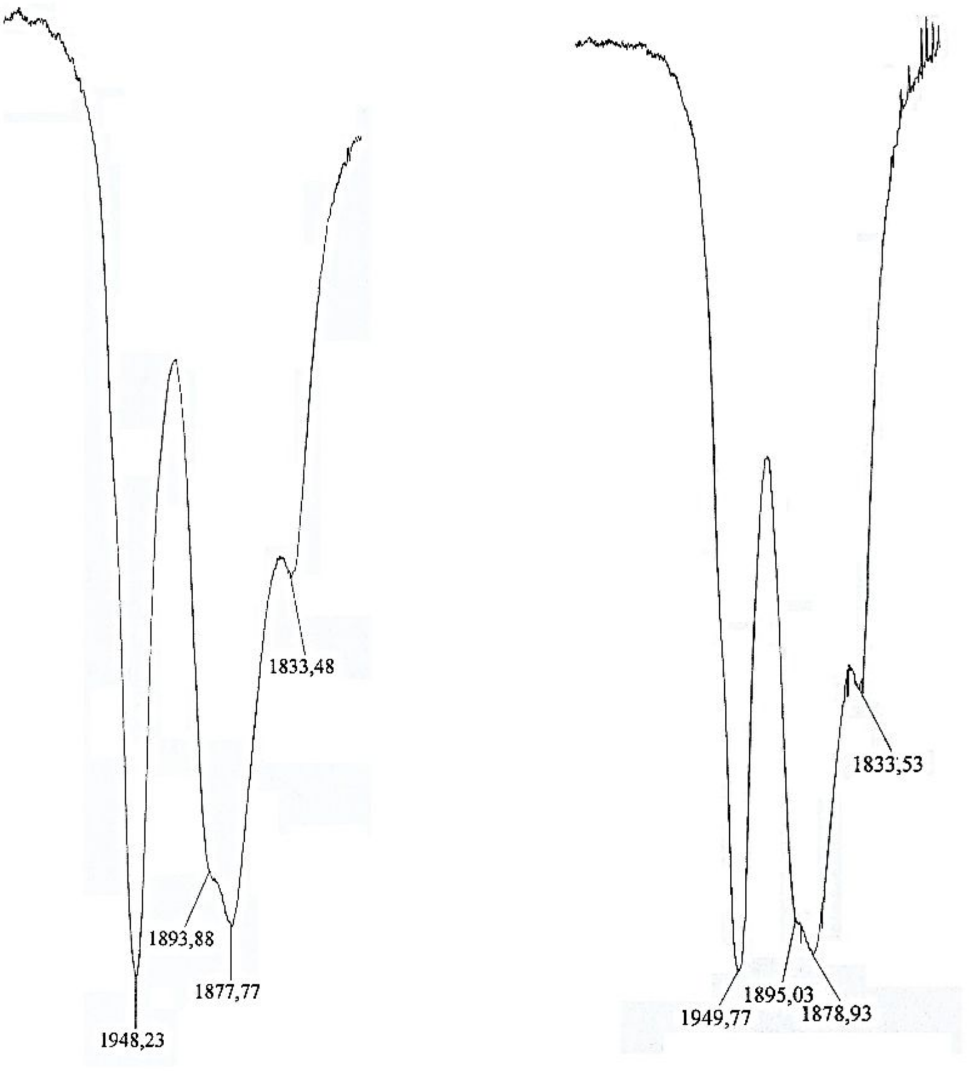

(a) (b)

Figure S6

IR spectra (ν_{CO} region) recorded in CH_3CN of (a) $[NEt_4]_3[Au_{1.09}Cu_{3.91}Fe_4(CO)_{16}]$ and (b) $[NEt_4]_3[Au_{1.15}Cu_{3.85}Fe_4(CO)_{16}]$. Spectral resolution 2 cm^{-1}.

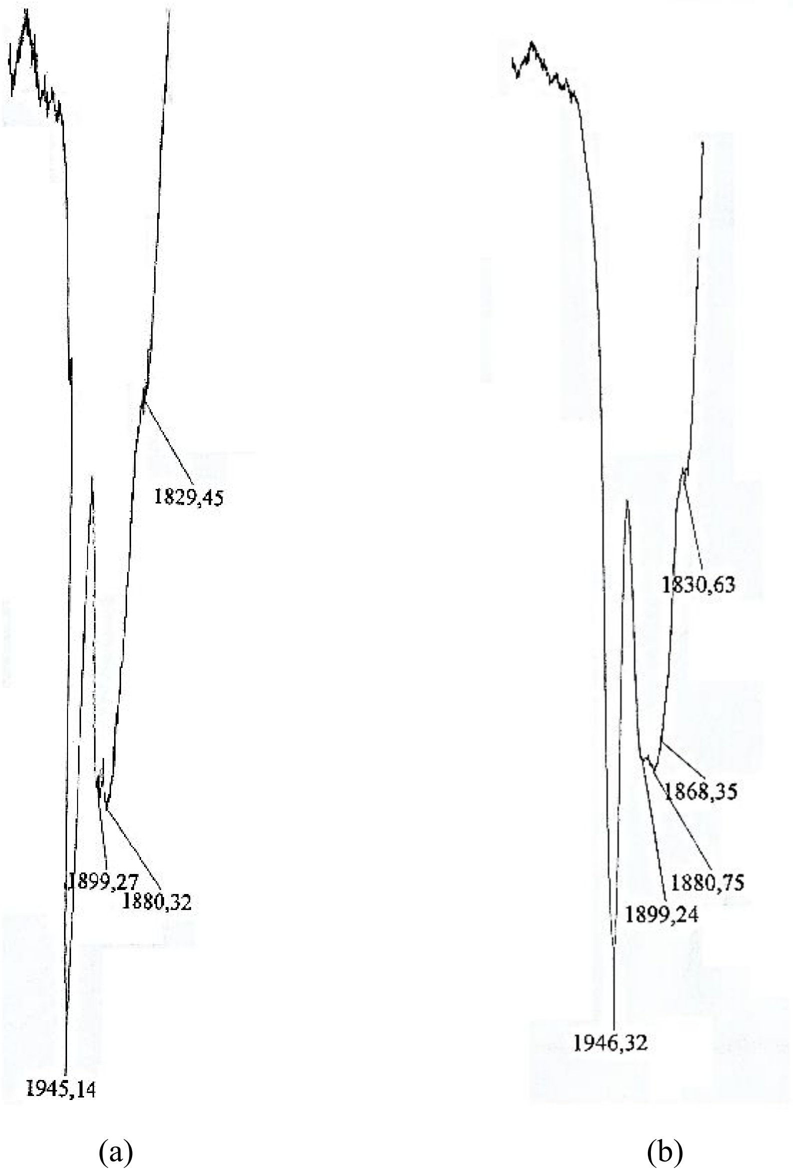

Figure S7

IR spectra (ν_{CO} region) recorded in CH_3CN of (a) $[NEt_4]_3[Au_{1.31}Cu_{3.69}Fe_4(CO)_{16}]$ and (b) $[NEt_4]_3[Au_{1.67}Cu_{3.33}Fe_4(CO)_{16}]$ (the bands at 1967.51 and 1904.64 cm^{-1} are due to impurities). Spectral resolution 2 cm^{-1}.

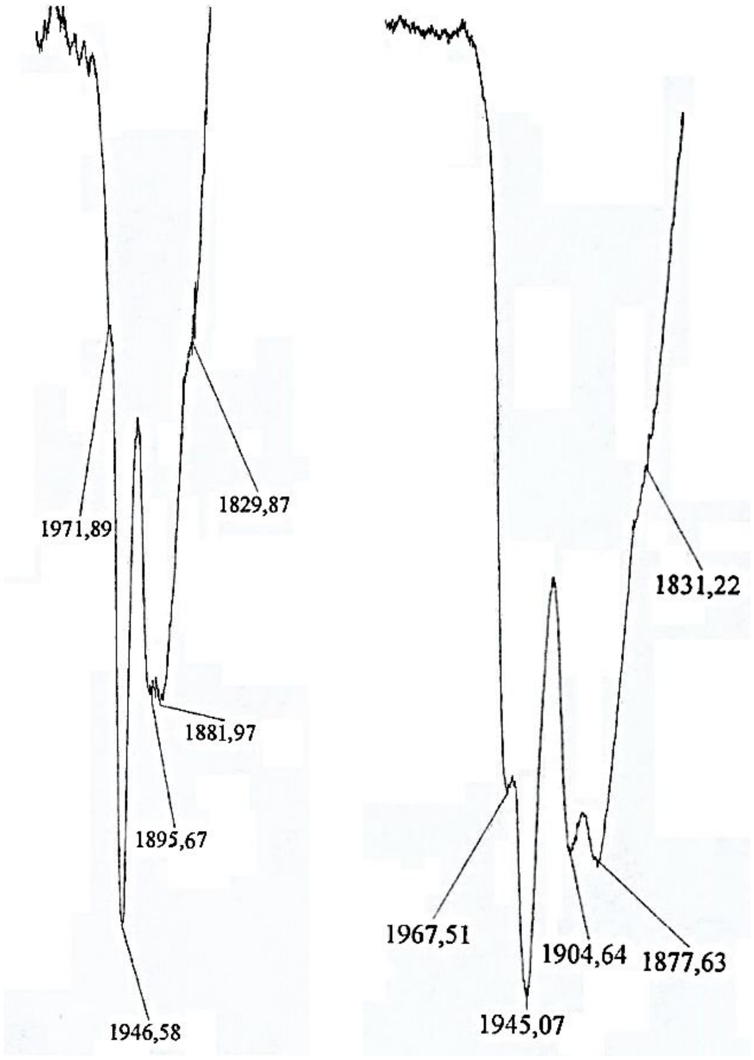

(a) (b)

Figure S8

IR spectra (ν_{CO} region) recorded in CH_3CN of (a) $[NEt_4]_3[Au_{2.48}Cu_{2.52}Fe_4(CO)_{16}]$ (the band at 1911.31 cm^{-1} is due to impurities) and (b) $[NEt_4]_3[Au_{2.18}Cu_{2.82}Fe_4(CO)_{16}]$ (the band at 1908.01 cm^{-1} is due to impurities). Spectral resolution 2 cm^{-1}.

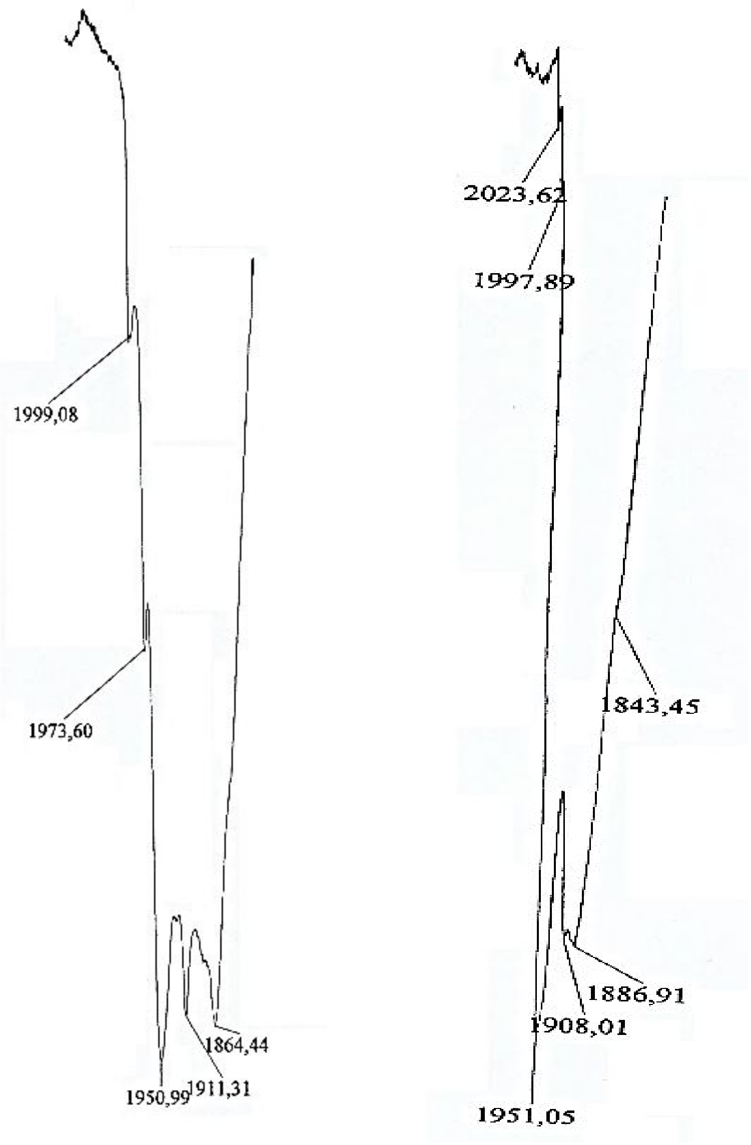

(a) (b)

Figure S9

IR spectra (ν_{CO} region) recorded in CH_3CN of (a) $[NEt_4]_3[Au_{0.64}Ag_{4.36}Fe_4(CO)_{16}]$ (the bands at 1926.98 and 1895.89 cm^{-1} are due to impurities) and (b) $[NEt_4]_3[Au_{0.82}Ag_{4.18}Fe_4(CO)_{16}]$. Spectral resolution 2 cm^{-1}.

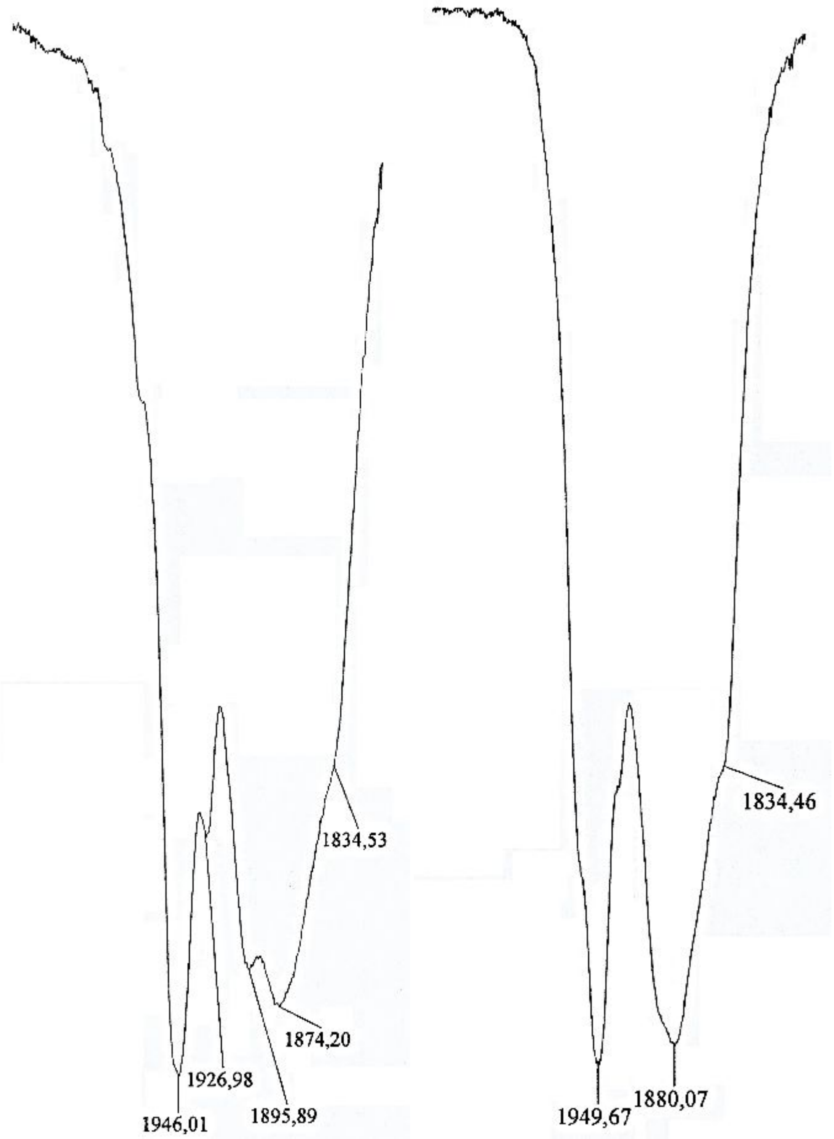

(a) (b)

Table S1

M-M and M-Fe distances of $[NEt_4]_3[Ag_xCu_{5-x}Fe_4(CO)_{16}]$ (x = 0-5).

**Entry***	**Composition**		**Distances**				
	**Ag**	**Cu**	**M(1)-M(2)**	**M(2)-M(2)**	**M(1)-Fe(1)**	**M(2)-Fe(1)**	**M(2)-Fe(2)**
**	0.00	5.00	2.53	2.69	2.49	2.43	2.39
1	1.02	3.98	2.61	2.78	2.50	2.47	2.44
10	3.30	1.70	2.73	2.96	2.64	2.57	2.53
11	3.45	1.55	2.73	2.96	2.64	2.58	2.53
3	4.25	0.75	2.77	2.99	2.68	2.61	2.56
8	4.37	0.63	2.77	3.00	2.69	2.62	2.56
7	4.81	0.19	2.79	3.01	2.70	2.64	2.58
4	4.88	0.12	2.79	3.01	2.70	2.64	2.58
9	4.90	0.10	2.80	3.01	2.71	2.64	2.58
5	4.92	0.08	2.79	3.01	2.70	2.64	2.58
2,6	5.00	0.00	2.79	3.01	2.70	2.64	2.58

* See Table 1 in the main text. Entries are listed in order of increasing Ag content.
** See Ref. 32 in the main text.

Scheme S1

Labelling of the atoms used in Tables S1-S3

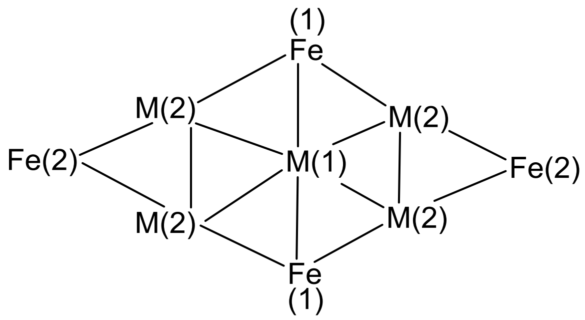

Table S2

M-M and M-Fe distances of $[NEt_4]_3[Au_xCu_{5-x}Fe_4(CO)_{16}]$ (x = 0-5).

Entry*	Composition		Distances				
	Au	Cu	M(1)-M(2)	M(2)-M(2)	M(1)-Fe(1)	M(2)-Fe(1)	M(2)-Fe(2)
**	0.00	5.00	2.53	2.69	2.49	2.43	2.39
20	1.09	3.91	2.60	2.79	2.51	2.46	2.44
12	1.15	3.85	2.61	2.80	2.51	2.46	2.45
13	1.31	3.69	2.61	2.81	2.51	2.47	2.46
14	1.67	3.33	2.63	2.82	2.52	2.48	2.48
16	2.18	2.82	2.65	2.83	2.55	2.50	2.50
15	2.48	2.52	2.66	2.85	2.56	2.52	2.51
17	2.73	2.27	2.66	2.85	2.57	2.52	2.52
18	4.59	0.41	2.75	2.96	2.64	2.59	2.56
19	4.62	0.38	2.76	2.96	2.64	2.60	2.56
***	5.00	0.00	2.78	3.02	2.69	2.61	2.56

* See Table 2 in the main text. Entries are listed in order of increasing Au content.
** See Ref. 32 in the main text.
*** See Ref. 28 in the main text.

Table S3

M-M and M-Fe distances of $[NEt_4]_3[Au_xAg_{5-x}Fe_4(CO)_{16}]$ (x = 0-5).

Entry*	Composition		Distances				
	Au	Ag	M(1)-M(2)	M(2)-M(2)	M(1)-Fe(1)	M(2)-Fe(1)	M(2)-Fe(2)
**	0.00	5.00	2.79	3.01	2.70	2.64	2.58
21	0.64	4.36	2.79	3.00	2.70	2.64	2.57
22	0.81	4.19	2.78	3.00	2.70	2.63	2.56
***	5.00	0.00	2.78	3.02	2.69	2.61	2.56

* See Table 3 in the main text. Entries are listed in order of increasing Au content.
** See Ref. 32 in the main text.
*** See Ref. 28 in the main text.

Figure S10

ESI-MS spectrum in CH_3CN (ES–) of $[NEt_4]_3[Cu_5Fe_4(CO)_{16}]$.

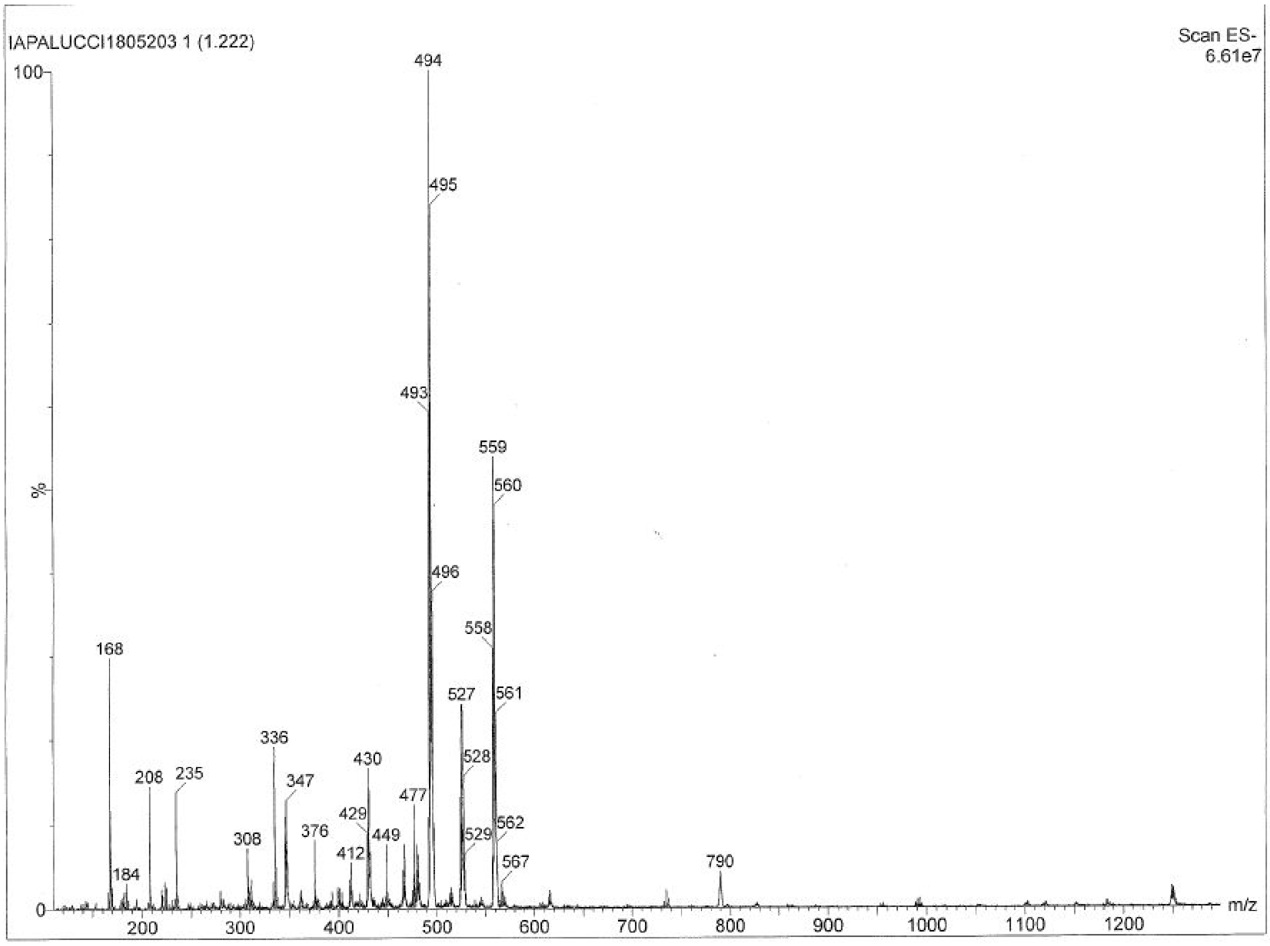

Table S4

Peak assignment of the ESI-MS spectrum (ES–) of $[NEt_4]_3[Cu_5Fe_4(CO)_{16}]$.

m/z	Relative intensity	Ion	Code
559	60	$\{[Cu_5Fe_4(CO)_{16}][NEt_4]\}^{2-}$	Cu_5+NEt_4
494	100	$[HCu_5Fe_4(CO)_{16}]^{2-}$	Cu_5
481	8	$[HCu_5Fe_4(CO)_{15}]^{2-}$	Cu_5(-1CO)
467	10	$[HCu_5Fe_4(CO)_{14}]^{2-}$	Cu_5(-2CO)

Figure S11

Isotopic pattern of the peak at m/z 494 of the ESI-MS spectrum in CH_3CN (ES–) of $[NEt_4]_3[Cu_5Fe_4(CO)_{16}]$. Upper trace: calculated isotopic patter for $[HCu_5Fe_4(CO)_{16}]^{2-}$. Middle trace: calculated isotopic patter for $[Cu_5Fe_4(CO)_{16}]^{2-}$. Lower trace: experimental isotopic pattern.

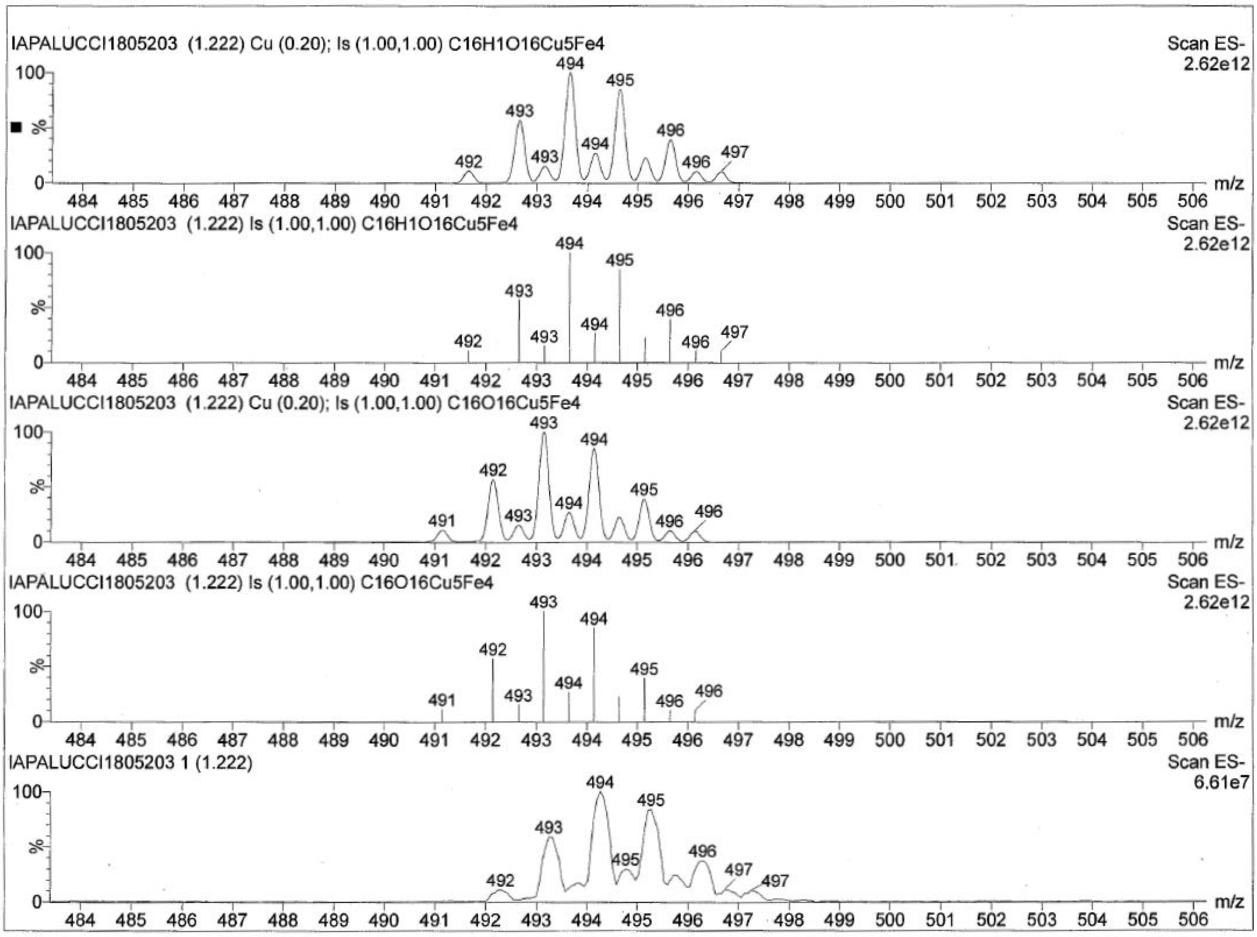

Figure S12

ESI-MS spectrum in CH_3CN (ES–) of $[NEt_4]_3[Ag_5Fe_4(CO)_{16}]$.

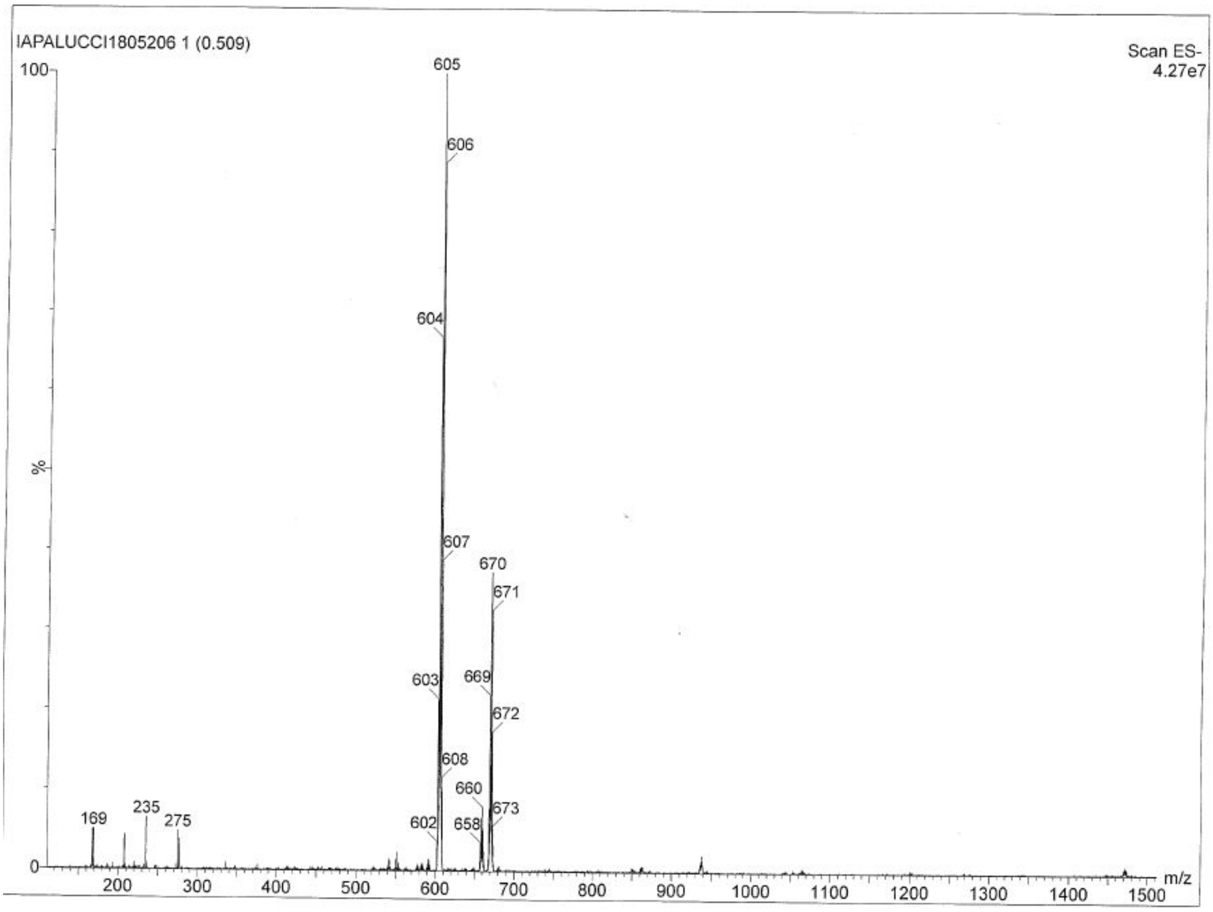

Table S5

Peak assignment of the ESI-MS spectrum (ES–) of $[NEt_4]_3[Cu_5Fe_4(CO)_{16}]$.

m/z	Relative intensity	Ion	Code
670	40	$\{[Ag_5Fe_4(CO)_{16}][NEt_4]\}^{2-}$	Ag_5+NEt_4
605	100	$[HAg_5Fe_4(CO)_{16}]^{2-}$	Ag_5

Figure S13

Isotopic pattern of the peak at m/z 605 of the ESI-MS spectrum in CH_3CN (ES–) of $[NEt_4]_3[Ag_5Fe_4(CO)_{16}]$. Upper trace: calculated isotopic patter for $[HAg_5Fe_4(CO)_{16}]^{2-}$. Middle trace: calculated isotopic patter for $[Ag_5Fe_4(CO)_{16}]^{2-}$. Lower trace: experimental isotopic pattern.

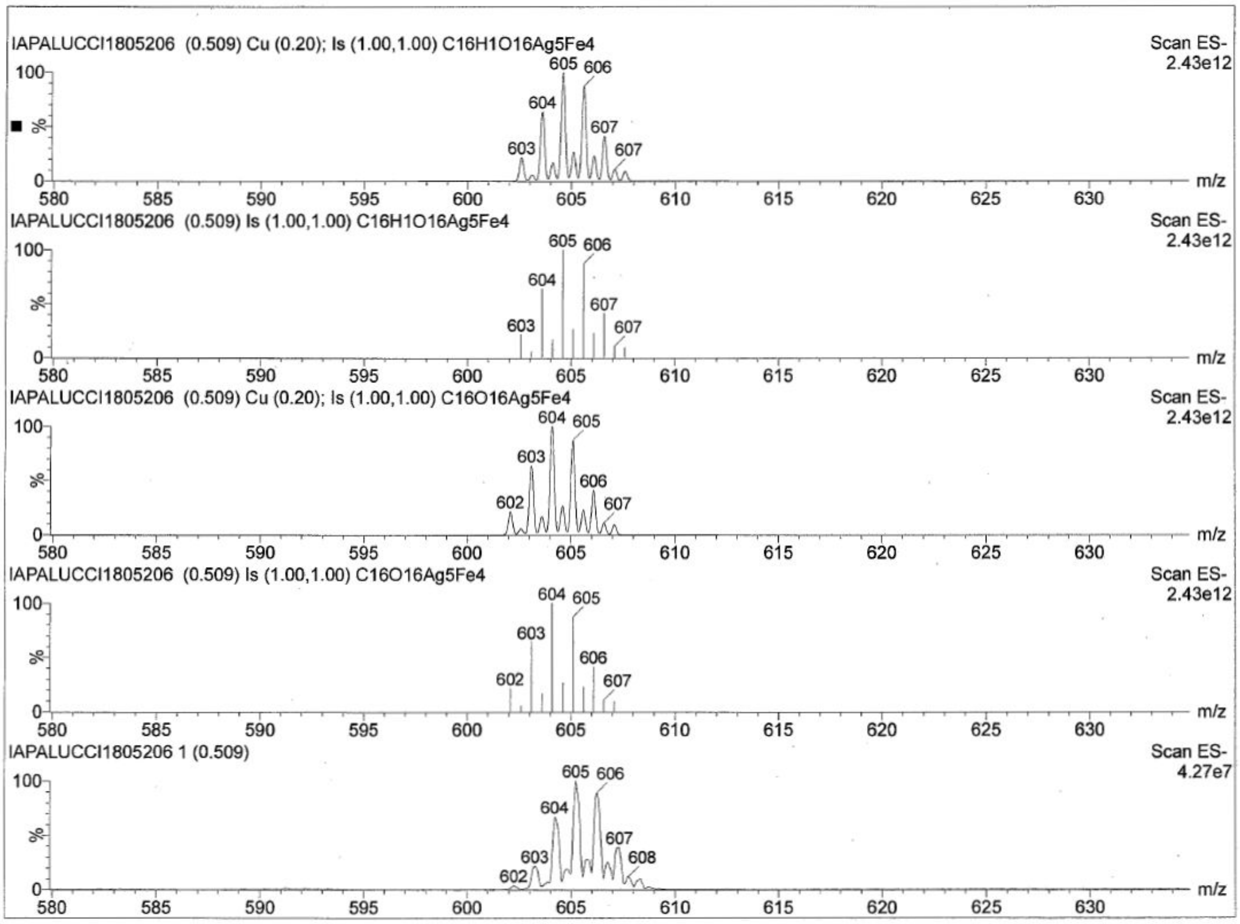

Figure S14

ESI-MS spectrum in CH_3CN (ES–) of $[NEt_4]_3[Ag_{1.02}Cu_{3.98}Fe_4(CO)_{16}]$.

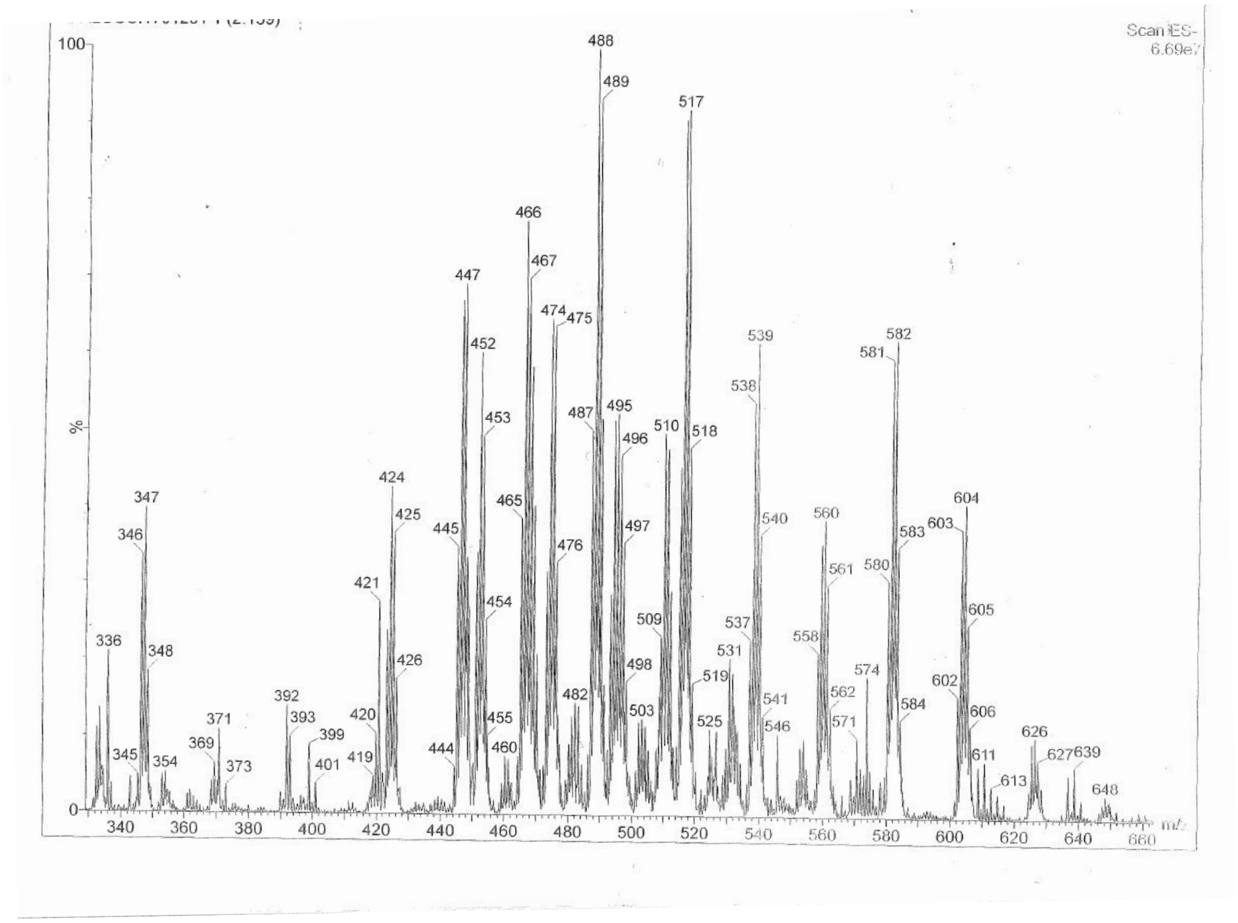

Table S6

Peak assignment of the ESI-MS spectrum (ES–) of $[NEt_4]_3[Ag_{1.02}Cu_{3.98}Fe_4(CO)_{16}]$.

m/z	Relative intensity	Ion	Code
604	40	$\{[Ag_2Cu_3Fe_4(CO)_{16}][NEt_4]\}^{2-}$	Ag_2Cu_3+NEt_4
582	60	$\{[AgCu_4Fe_4(CO)_{16}][NEt_4]\}^{2-}$	$AgCu_4$+NEt_4
560	35	$\{[Cu_5Fe_4(CO)_{16}][NEt_4]\}^{2-}$	Cu_5+NEt_4
539	60	$[HAg_2Cu_3Fe_4(CO)_{16}]^{2-}$	Ag_2Cu_3
525	10	$[HAg_2Cu_3Fe_4(CO)_{15}]^{2-}$	Ag_2Cu_3(-1CO)
517	90	$[HAgCu_4Fe_4(CO)_{16}]^{2-}$	$AgCu_4$
510	50	$[HAg_2Cu_3Fe_4(CO)_{14}]^{2-}$	Ag_2Cu_3(-2CO)
503	10	$[HAgCu_4Fe_4(CO)_{15}]^{2-}$	$AgCu_4$(-1CO)
497	30	$[HAg_2Cu_3Fe_4(CO)_{13}]^{2-}$	Ag_2Cu_3(-3CO)
495	50	$[HCu_5Fe_4(CO)_{16}]^{2-}$	Cu_5
488	100	$[HAgCu_4Fe_4(CO)_{14}]^{2-}$	$AgCu_4$(-2CO)
482	15	$[HCu_5Fe_4(CO)_{15}]^{2-}$	Cu_5(-1CO)
474	65	$[HAgCu_4Fe_4(CO)_{13}]^{2-}$	$AgCu_4$(-3CO)
466	75	$[HCu_5Fe_4(CO)_{14}]^{2-}$	Cu_5(-2CO)
460	5	$[HAgCu_4Fe_4(CO)_{12}]^{2-}$	$AgCu_4$(-4CO)
452	60	$[HCu_5Fe_4(CO)_{13}]^{2-}$	Cu_5(-3CO)
447	70	$[HAgCu_4Fe_4(CO)_{11}]^{2-}$	$AgCu_4$(-5CO)

Figure S15

Isotopic pattern of the peak at m/z 466 of the ESI-MS spectrum in CH_3CN (ES–) of $[NEt_4]_3[Ag_{1.02}Cu_{3.98}Fe_4(CO)_{16}]$. Upper trace: calculated isotopic patter for $[HCu_5Fe_4(CO)_{14}]^{2-}$. Middle trace: calculated isotopic patter for $[Cu_5Fe_4(CO)_{14}]^{2-}$. Lower trace: experimental isotopic pattern.

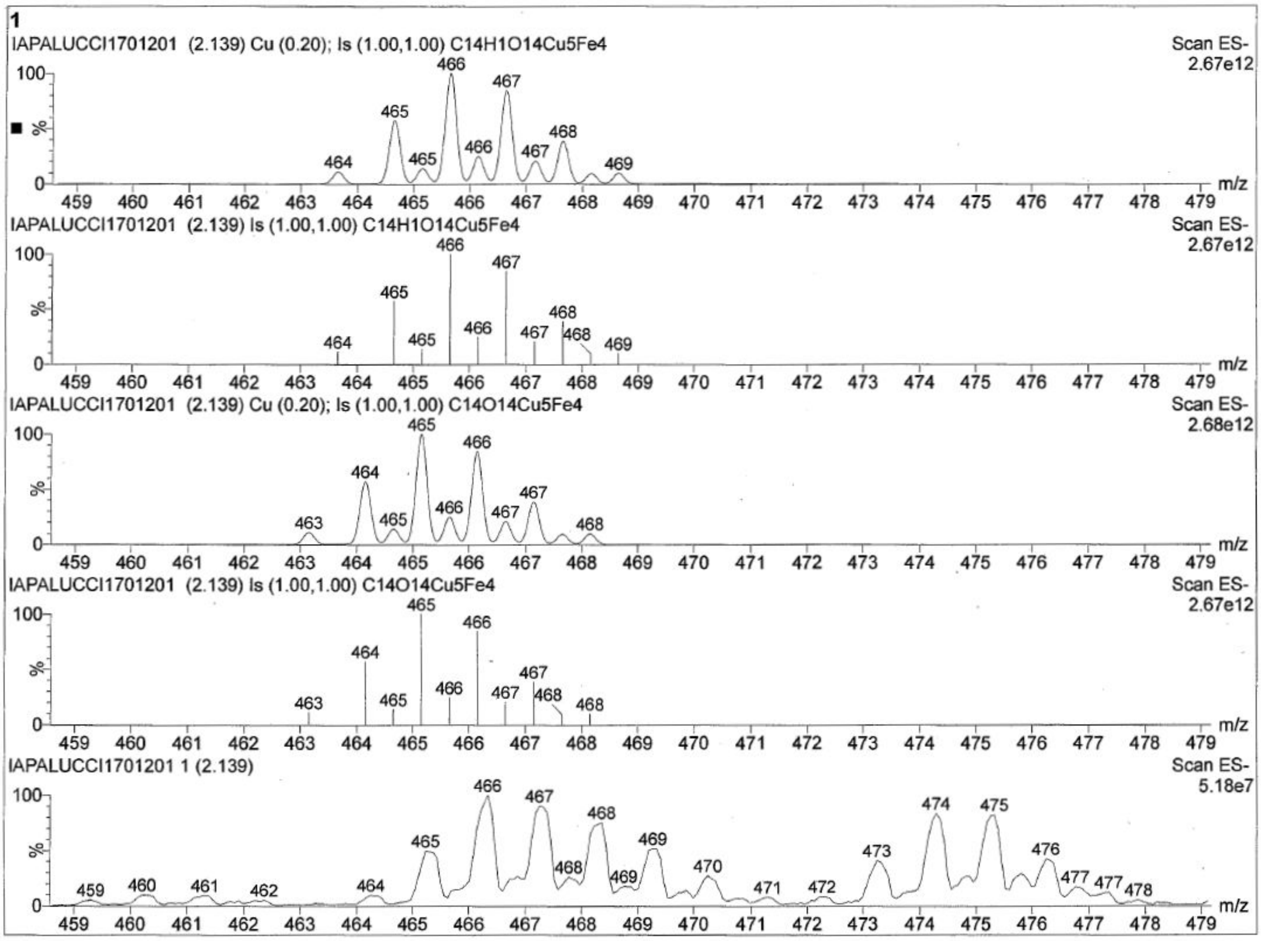

Figure S16

Isotopic pattern of the peak at m/z 488 of the ESI-MS spectrum in CH_3CN (ES–) of $[NEt_4]_3[Ag_{1.02}Cu_{3.98}Fe_4(CO)_{16}]$. Upper trace: calculated isotopic patter for $[HAgCu_4Fe_4(CO)_{14}]^{2-}$. Middle trace: calculated isotopic patter for $[AgCu_4Fe_4(CO)_{14}]^{2-}$. Lower trace: experimental isotopic pattern.

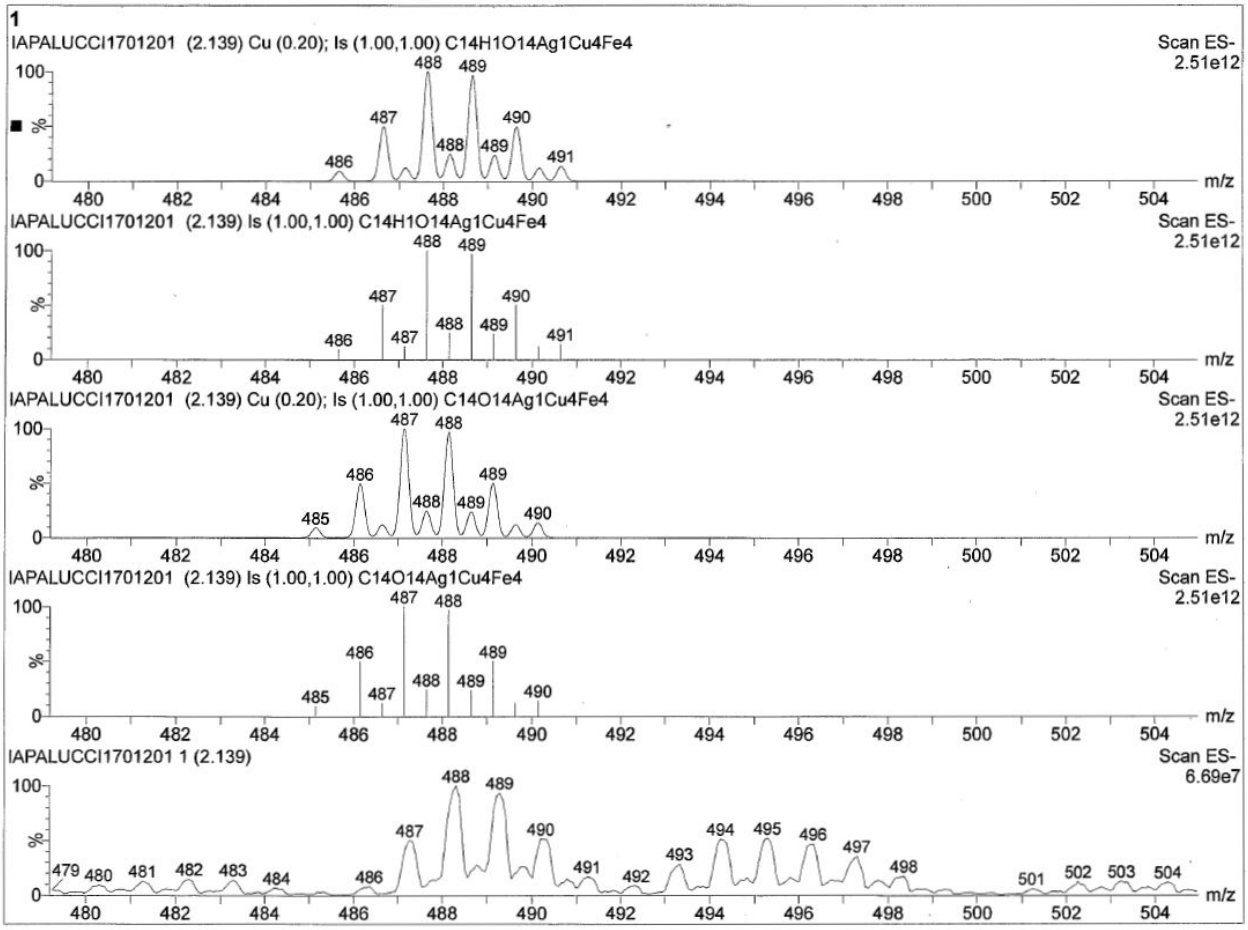

Figure S17

Isotopic pattern of the peak at m/z 517 of the ESI-MS spectrum in CH_3CN (ES–) of $[NEt_4]_3[Ag_{1.02}Cu_{3.98}Fe_4(CO)_{16}]$. Upper trace: calculated isotopic patter for $[HAgCu_4Fe_4(CO)_{16}]^{2-}$. Middle trace: calculated isotopic patter for $[AgCu_4Fe_4(CO)_{16}]^{2-}$. Lower trace: experimental isotopic pattern.

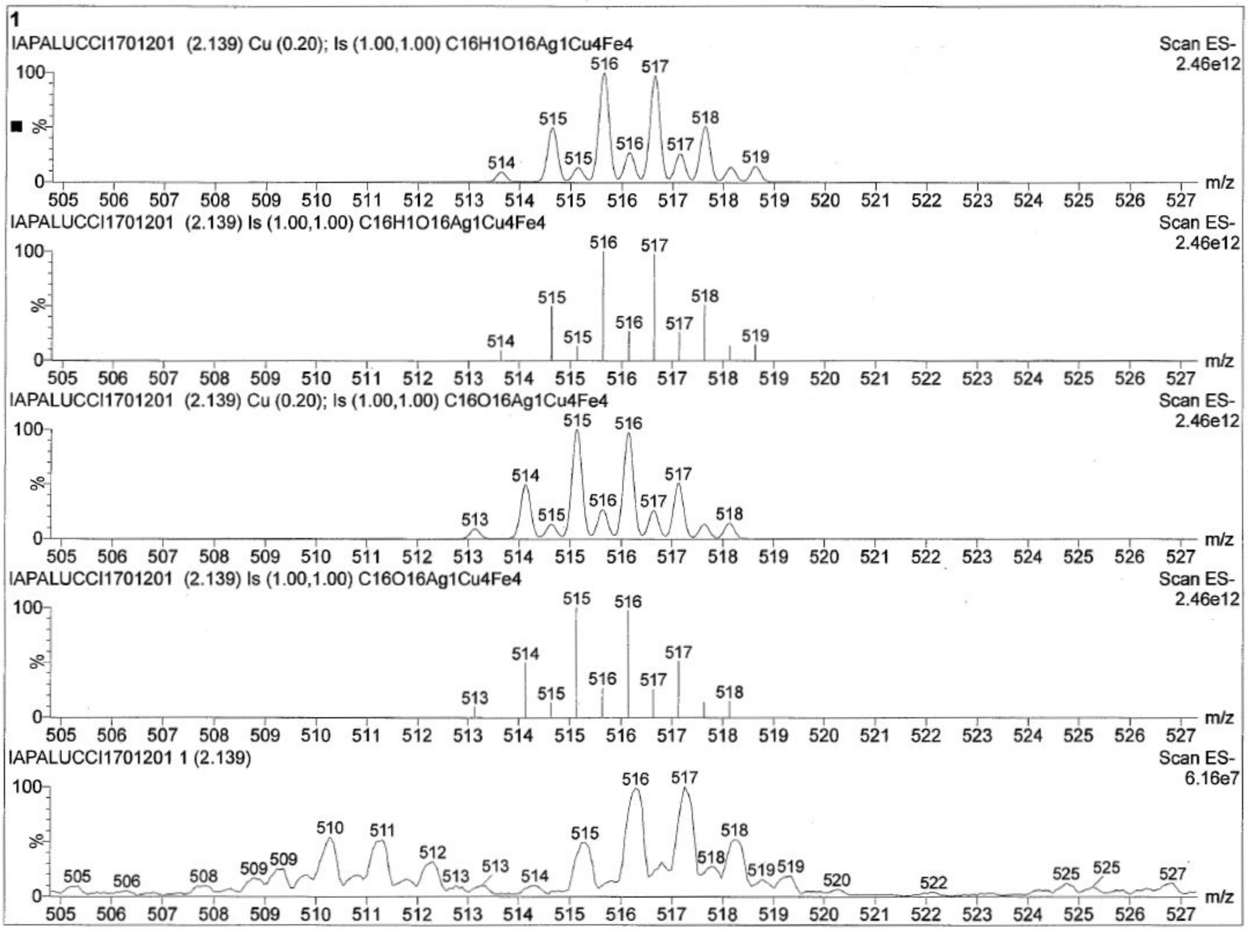

Figure S18

Isotopic pattern of the peak at m/z 539 of the ESI-MS spectrum in CH_3CN (ES–) of $[NEt_4]_3[Ag_{1.02}Cu_{3.98}Fe_4(CO)_{16}]$. Upper trace: calculated isotopic patter for $[HAg_2Cu_3Fe_4(CO)_{16}]^{2-}$. Middle trace: calculated isotopic patter for $[Ag_2Cu_3Fe_4(CO)_{16}]^{2-}$. Lower trace: experimental isotopic pattern.

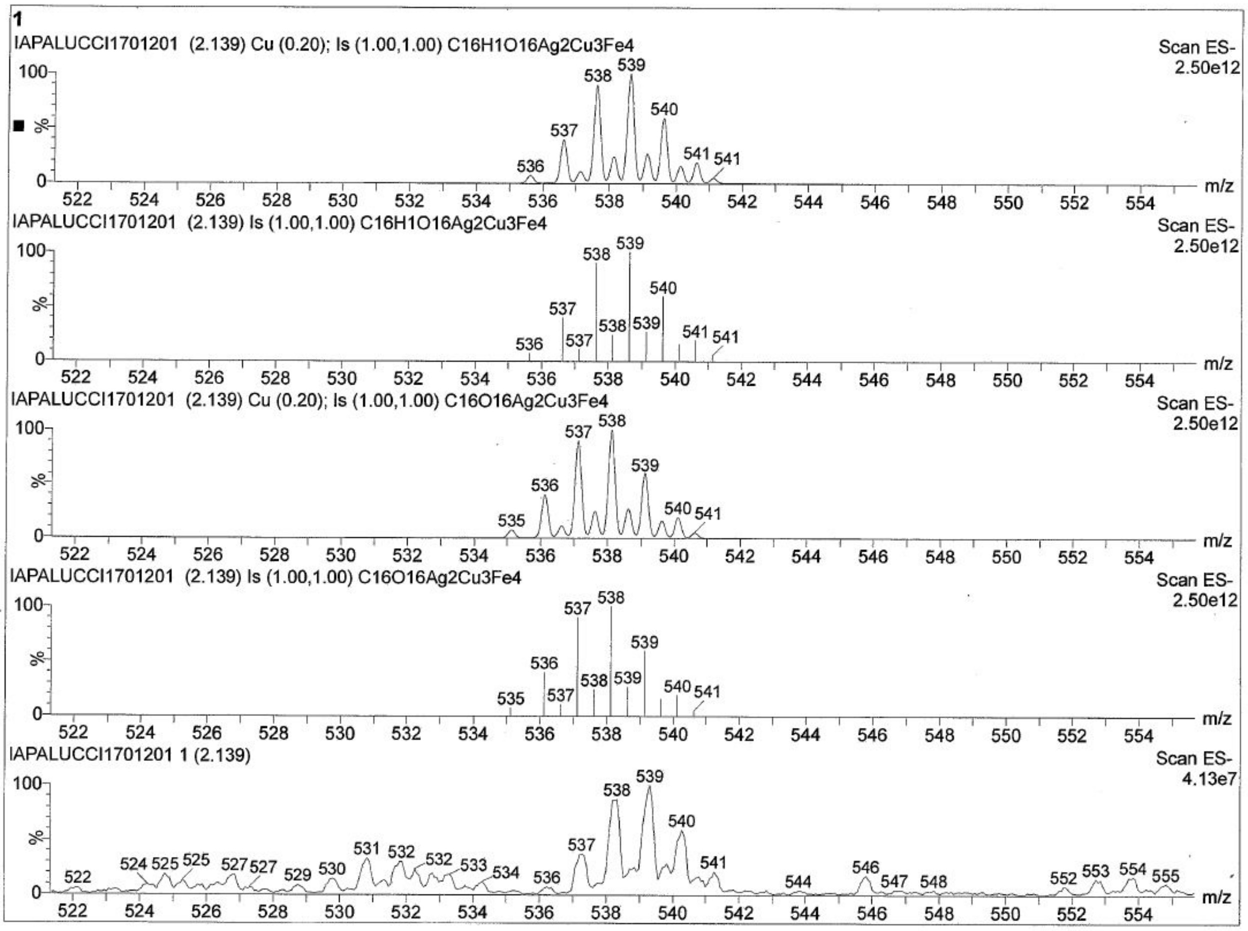

Figure S19

ESI-MS spectrum in CH_3CN (ES–) of $[NEt_4]_3[Au_{1.32}Cu_{3.68}Fe_4(CO)_{16}]$.

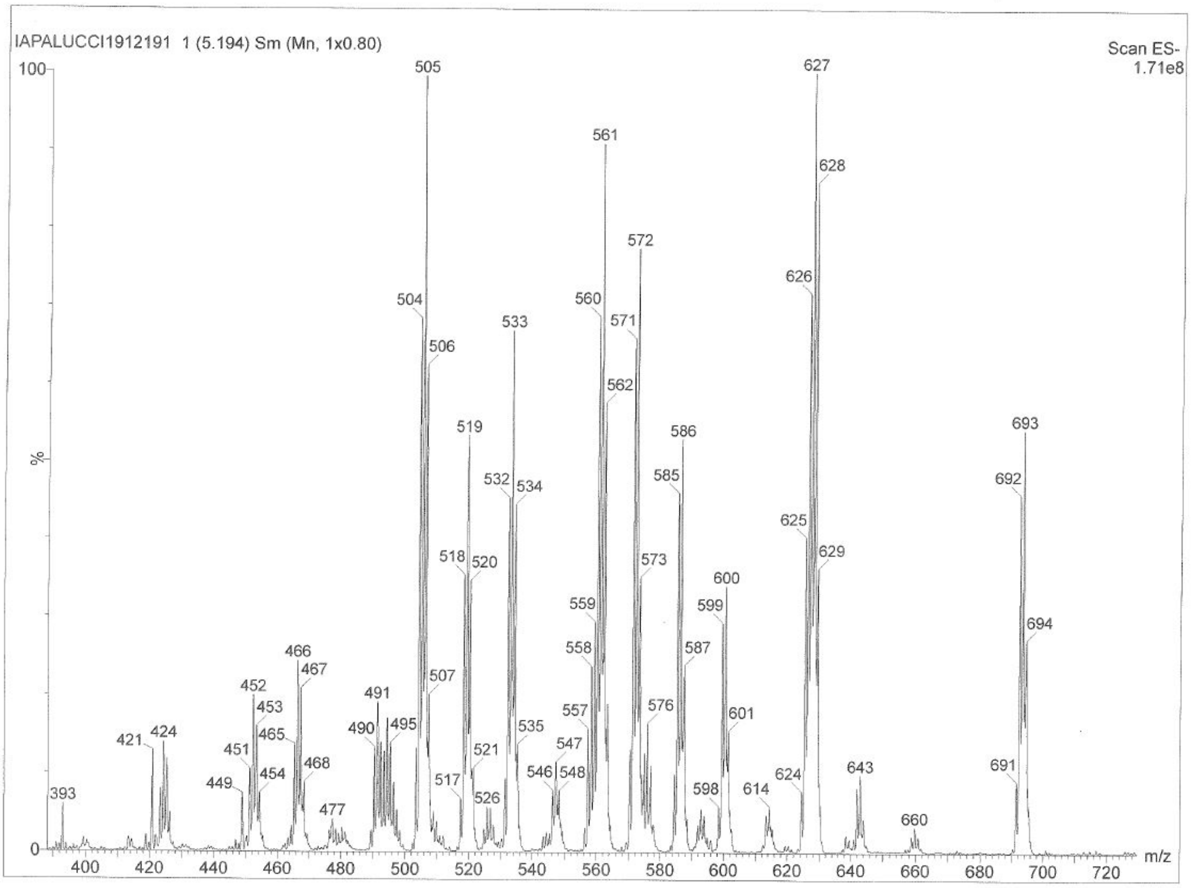

Table S7

Peak assignment of the ESI-MS spectrum (ES–) of $[NEt_4]_3[Au_{1.32}Cu_{3.68}Fe_4(CO)_{16}]$.

m/z	Relative intensity	Ion	Code
693	55	$\{[Au_2Cu_3Fe_4(CO)_{16}][NEt_4]\}^{2-}$	Au_2Cu_3+NEt_4
629	40	$[HAu_2Cu_3Fe_4(CO)_{16}]^{2-}$	Au_2Cu_3
627	100	$\{[AuCu_4Fe_4(CO)_{16}][NEt_4]\}^{2-}$	$AuCu_4$+NEt_4
614	5	$[HAu_2Cu_3Fe_4(CO)_{15}]^{2-}$	Au_2Cu_3(-1CO)
600	30	$[HAu_2Cu_3Fe_4(CO)_{14}]^{2-}$	Au_2Cu_3(-2CO)
586	50	$[HAu_2Cu_3Fe_4(CO)_{13}]^{2-}$	Au_2Cu_3(-3CO)
572	80	$[HAu_2Cu_3Fe_4(CO)_{12}]^{2-}$	Au_2Cu_3(-4CO)
561	90	$[HAuCu_4Fe_4(CO)_{16}]^{2-}$	$AuCu_4$
558	20	$[HAu_2Cu_3Fe_4(CO)_{11}]^{2-}$	Au_2Cu_3(-5CO)
547	10	$[HAuCu_4Fe_4(CO)_{15}]^{2-}$	$AuCu_4$(-1CO)
533	65	$[HAuCu_4Fe_4(CO)_{14}]^{2-}$	$AuCu_4$(-2CO)
519	50	$[HAuCu_4Fe_4(CO)_{13}]^{2-}$	$AuCu_4$(-3CO)
505	100	$[HAuCu_4Fe_4(CO)_{12}]^{2-}$	$AuCu_4$(-4CO)
495	15	$[HCu_5Fe_4(CO)_{16}]^{2-}$	Cu_5
491	20	$[HAuCu_4Fe_4(CO)_{11}]^{2-}$	$AuCu_4$(-5CO)
466	75	$[HCu_5Fe_4(CO)_{14}]^{2-}$	Cu_5(-2CO)
452	60	$[HCu_5Fe_4(CO)_{13}]^{2-}$	Cu_5(-3CO)

Figure S20

Isotopic pattern of the peak at m/z 466 of the ESI-MS spectrum in CH_3CN (ES–) of $[NEt_4]_3[Au_{1.32}Cu_{3.68}Fe_4(CO)_{16}]$. Upper trace: calculated isotopic patter for $[HCu_5Fe_4(CO)_{14}]^{2-}$. Middle trace: calculated isotopic patter for $[Cu_5Fe_4(CO)_{14}]^{2-}$. Lower trace: experimental isotopic pattern.

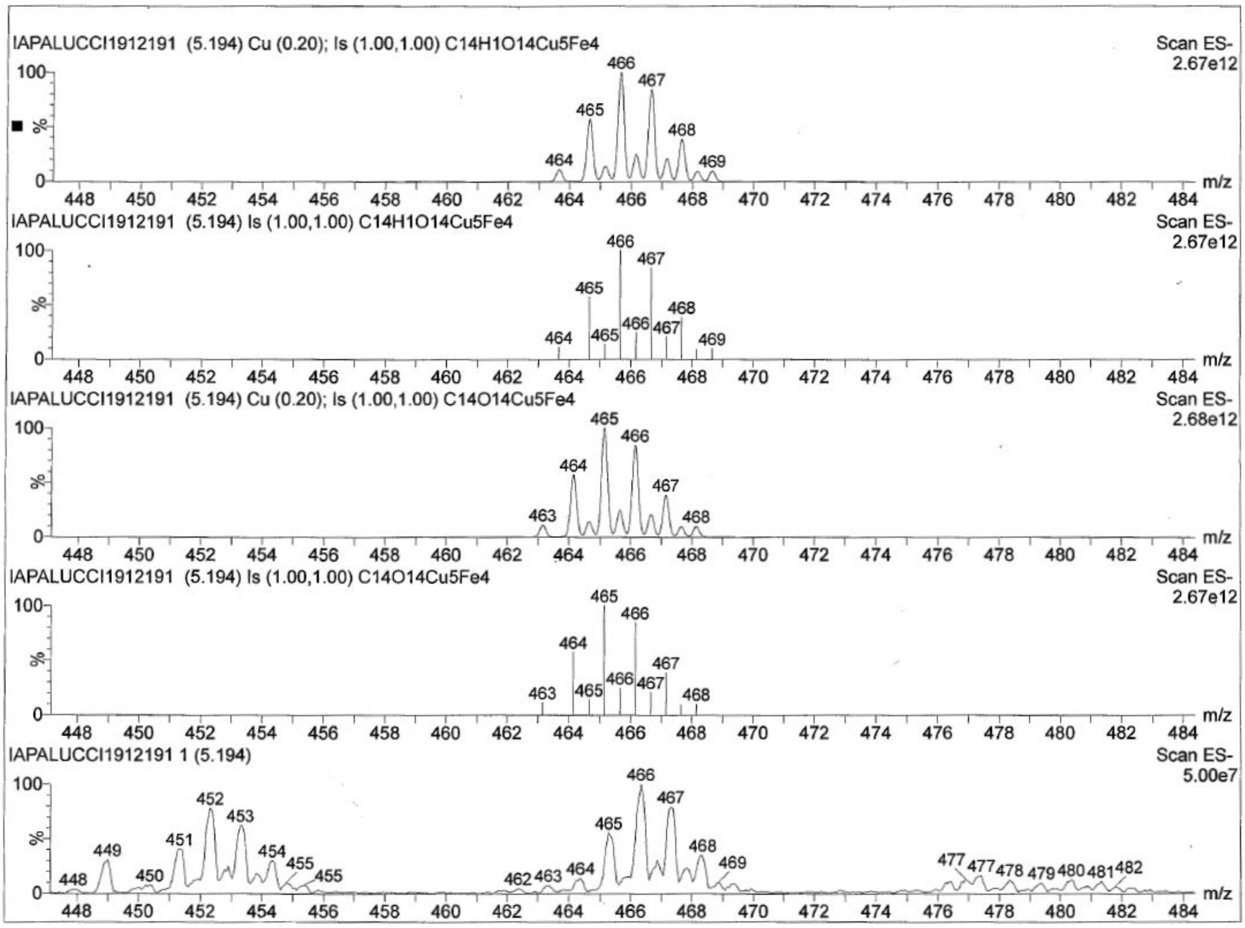

Figure S21

Isotopic pattern of the peak at m/z 505 of the ESI-MS spectrum in CH_3CN (ES–) of $[NEt_4]_3[Au_{1.32}Cu_{3.68}Fe_4(CO)_{16}]$. Upper trace: calculated isotopic patter for $[HAuCu_4Fe_4(CO)_{12}]^{2-}$. Middle trace: calculated isotopic patter for $[AuCu_4Fe_4(CO)_{12}]^{2-}$. Lower trace: experimental isotopic pattern.

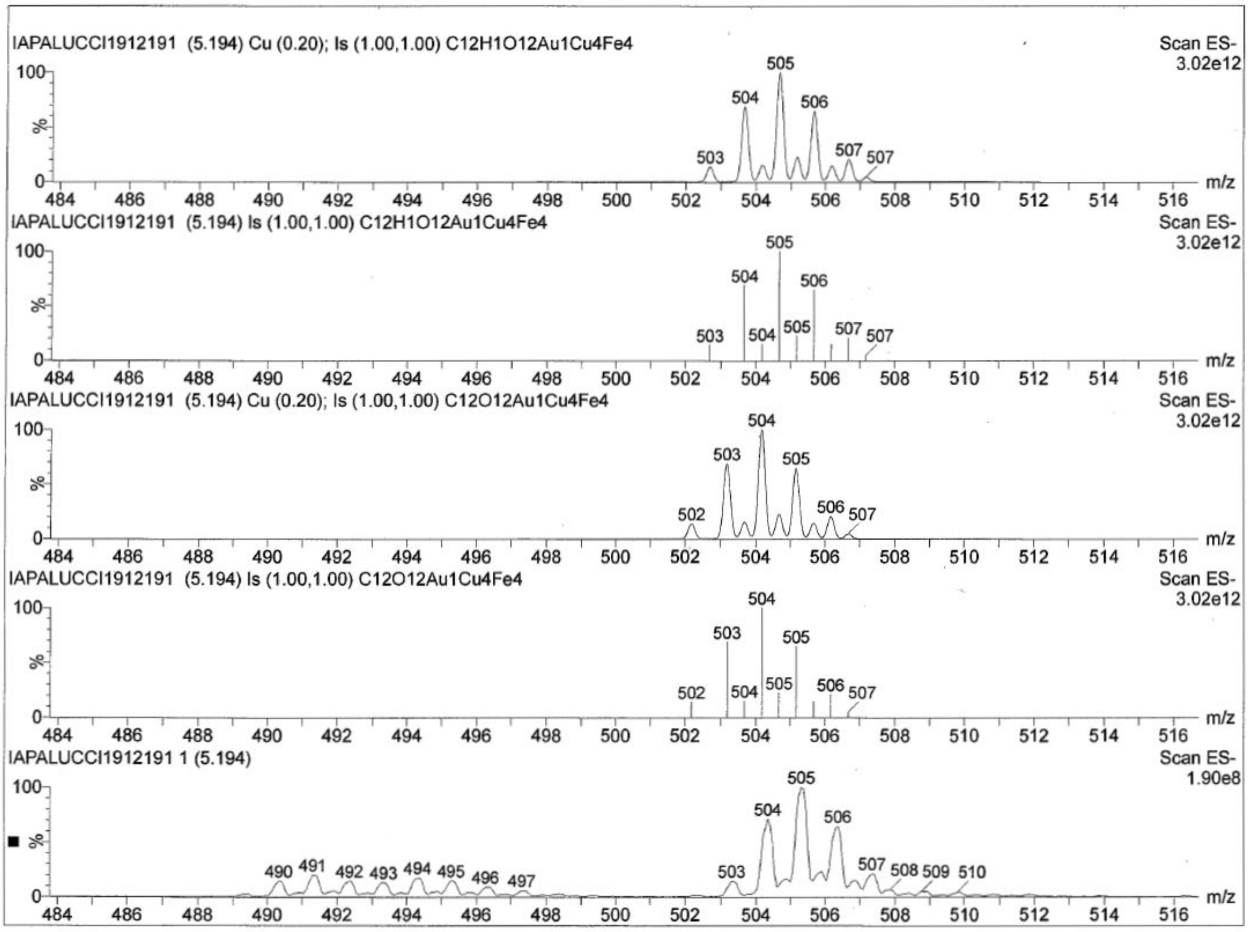

Figure S22

Isotopic pattern of the peak at m/z 561 of the ESI-MS spectrum in CH_3CN (ES–) of $[NEt_4]_3[Au_{1.32}Cu_{3.68}Fe_4(CO)_{16}]$. Upper trace: calculated isotopic patter for $[HAuCu_4Fe_4(CO)_{16}]^{2-}$. Middle trace: calculated isotopic patter for $[AuCu_4Fe_4(CO)_{16}]^{2-}$. Lower trace: experimental isotopic pattern.

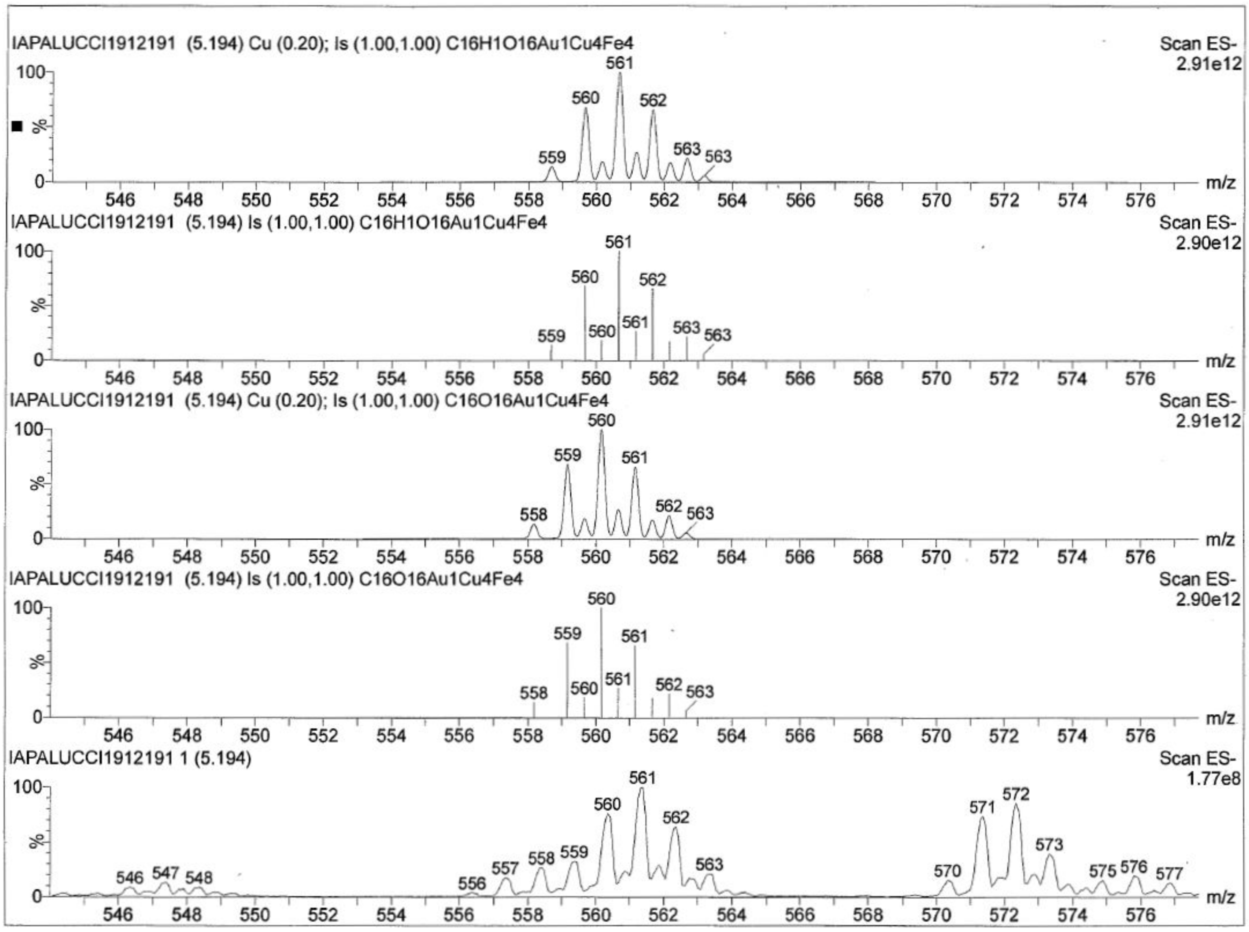

Figure S23

Isotopic pattern of the peak at m/z 572 of the ESI-MS spectrum in CH_3CN (ES–) of $[NEt_4]_3[Au_{1.32}Cu_{3.68}Fe_4(CO)_{16}]$. Upper trace: calculated isotopic patter for $[HAu_2Cu_3Fe_4(CO)_{12}]^{2-}$. Middle trace: calculated isotopic patter for $[Au_2Cu_3Fe_4(CO)_{12}]^{2-}$. Lower trace: experimental isotopic pattern.

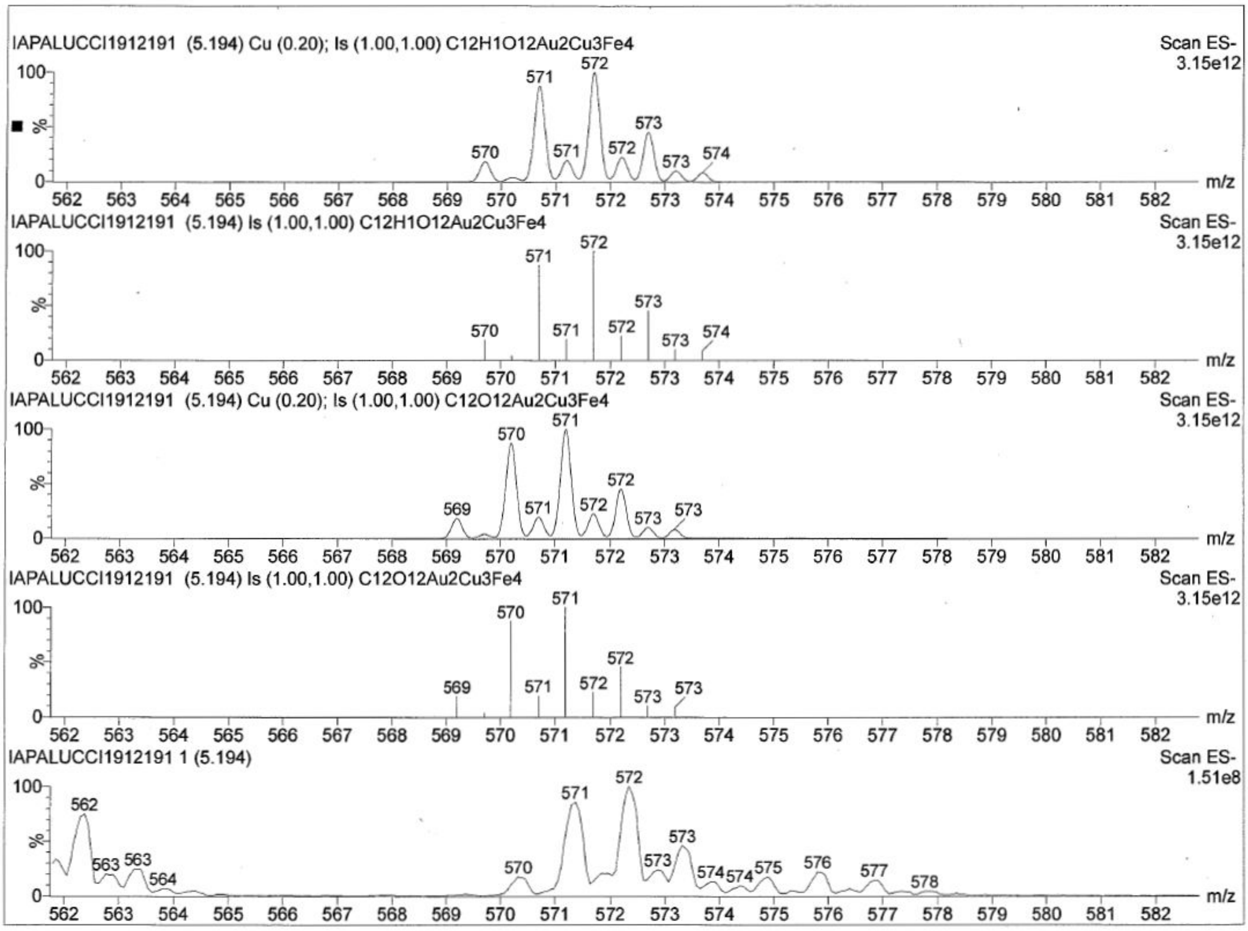

Figure S24

ESI-MS spectrum in CH_3CN (ES–) of $[NEt_4]_3[Au_{2.48}Cu_{2.52}Fe_4(CO)_{16}]$.

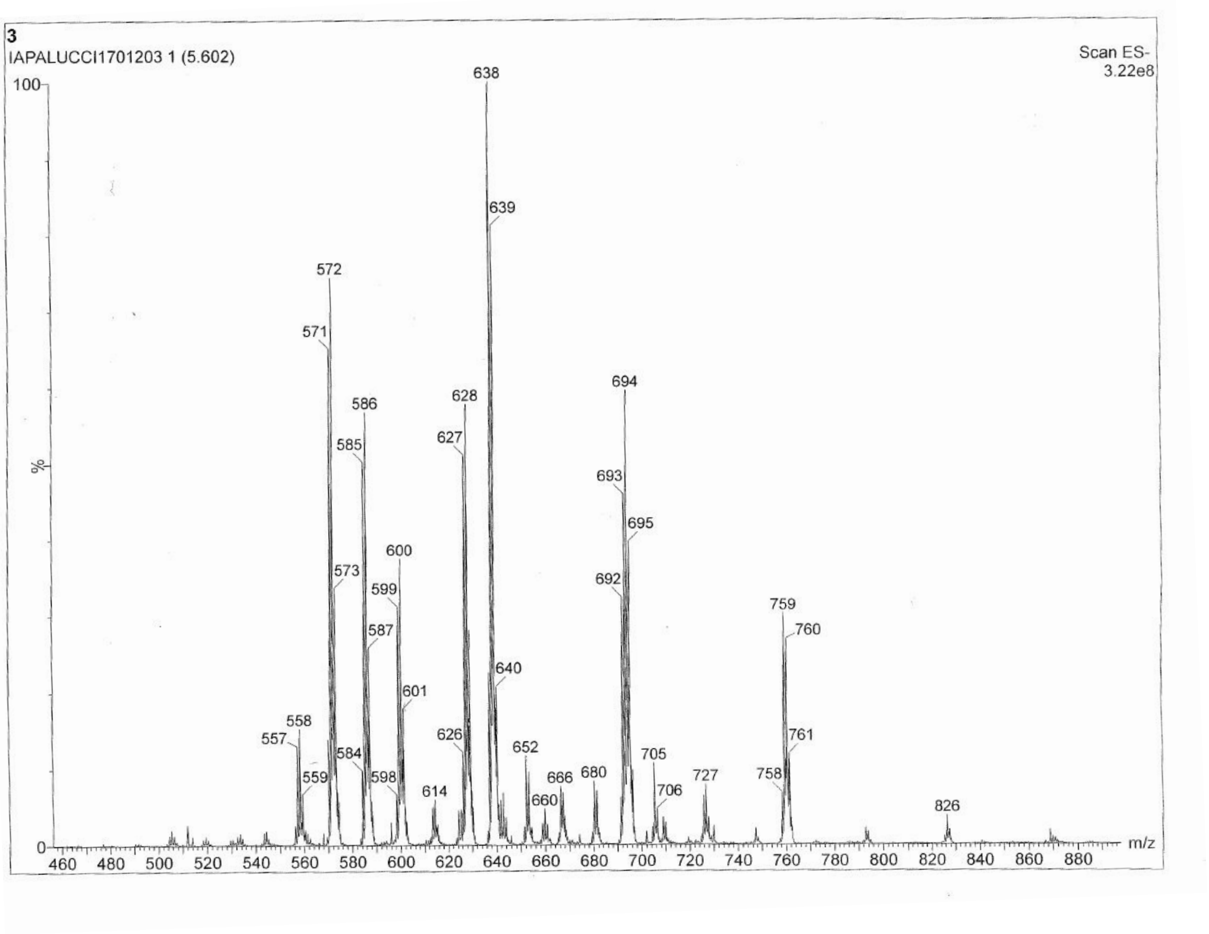

Table S8

Peak assignment of the ESI-MS spectrum (ES–) of $[NEt_4]_3[Au_{2.48}Cu_{2.52}Fe_4(CO)_{16}]$.

m/z	Relative intensity	Ion	Code
759	30	$\{[Au_3Cu_2Fe_4(CO)_{16}][NEt_4]\}^{2-}$	Au_3Cu_2+NEt_4
694	60	$[HAu_3Cu_2Fe_4(CO)_{16}]^{2-}$	Au_3Cu_2
692	35	$\{[Au_2Cu_3Fe_4(CO)_{16}][NEt_4]\}^{2-}$	Au_2Cu_3+NEt_4
680	10	$[HAu_3Cu_2Fe_4(CO)_{15}]^{2-}$	Au_3Cu_2(-1CO)
666	5	$[HAu_3Cu_2Fe_4(CO)_{14}]^{2-}$	Au_3Cu_2(-2CO)
652	10	$[HAu_3Cu_2Fe_4(CO)_{13}]^{2-}$	Au_3Cu_2(-3CO)
638	100	$[HAu_3Cu_2Fe_4(CO)_{12}]^{2-}$	Au_3Cu_2(-4CO)
628	60	$[HAu_2Cu_3Fe_4(CO)_{16}]^{2-}$	Au_2Cu_3
626	10	$[HAu_3Cu_2Fe_4(CO)_{11}]^{2-}$	Au_3Cu_2(-5CO)
614	5	$[HAu_2Cu_3Fe_4(CO)_{15}]^{2-}$	Au_2Cu_3(-1CO)
600	30	$[HAu_2Cu_3Fe_4(CO)_{14}]^{2-}$	Au_2Cu_3(-2CO)
586	60	$[HAu_2Cu_3Fe_4(CO)_{13}]^{2-}$	Au_2Cu_3(-3CO)
572	80	$[HAu_2Cu_3Fe_4(CO)_{12}]^{2-}$	Au_2Cu_3(-4CO)
558	15	$[HAu_2Cu_3Fe_4(CO)_{11}]^{2-}$	Au_2Cu_3(-5CO)

Figure S25

Isotopic pattern of the peak at m/z 572 of the ESI-MS spectrum in CH_3CN (ES–) of $[NEt_4]_3[Au_{2.48}Cu_{2.52}Fe_4(CO)_{16}]$. Upper trace: calculated isotopic patter for $[HAu_2Cu_3Fe_4(CO)_{12}]^{2-}$. Middle trace: calculated isotopic patter for $[Au_2Cu_3Fe_4(CO)_{12}]^{2-}$. Lower trace: experimental isotopic pattern.

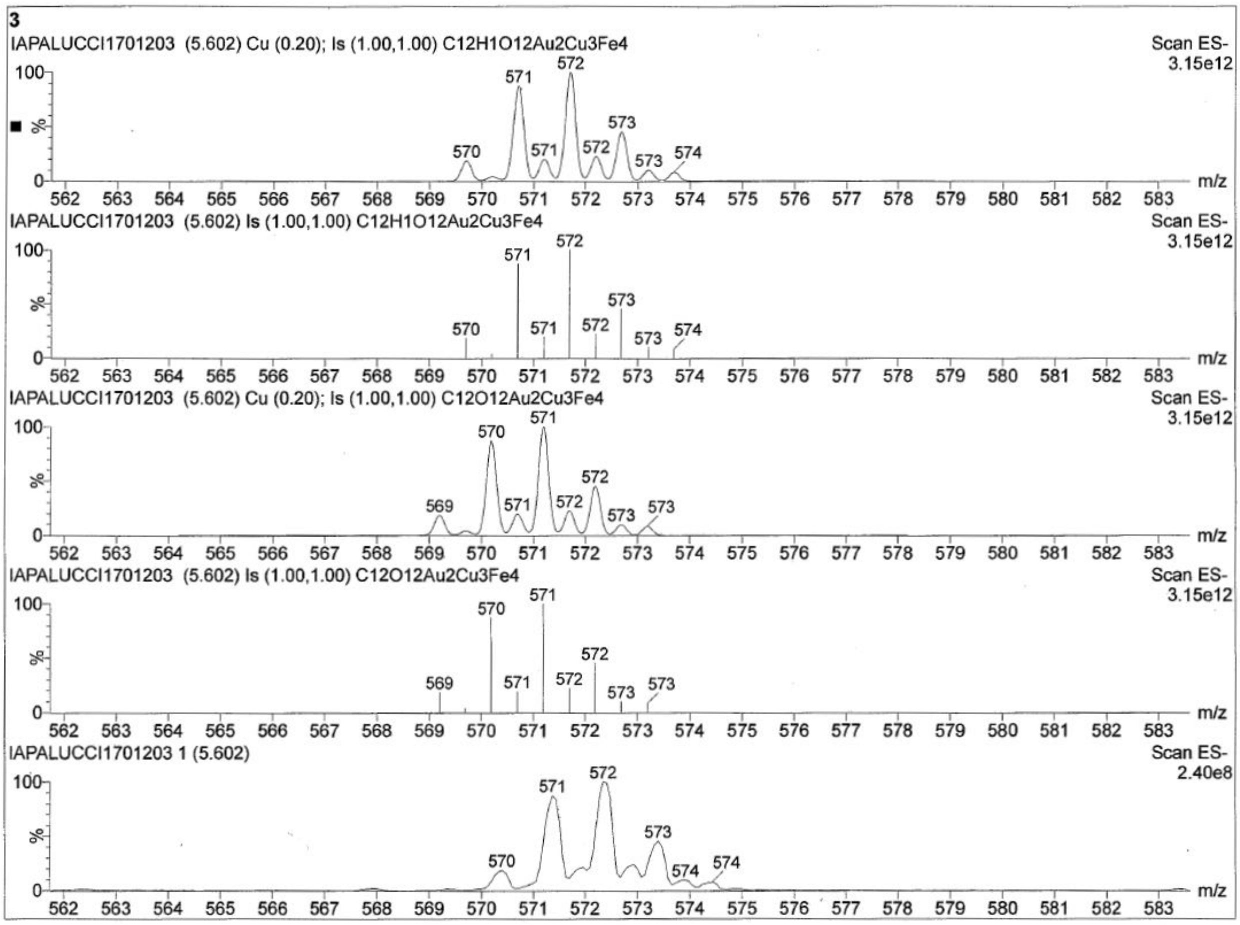

Figure S26

Isotopic pattern of the peak at m/z 586 of the ESI-MS spectrum in CH_3CN (ES–) of $[NEt_4]_3[Au_{2.48}Cu_{2.52}Fe_4(CO)_{16}]$. Upper trace: calculated isotopic patter for $[HAu_2Cu_3Fe_4(CO)_{13}]^{2-}$. Middle trace: calculated isotopic patter for $[Au_2Cu_3Fe_4(CO)_{13}]^{2-}$. Lower trace: experimental isotopic pattern.

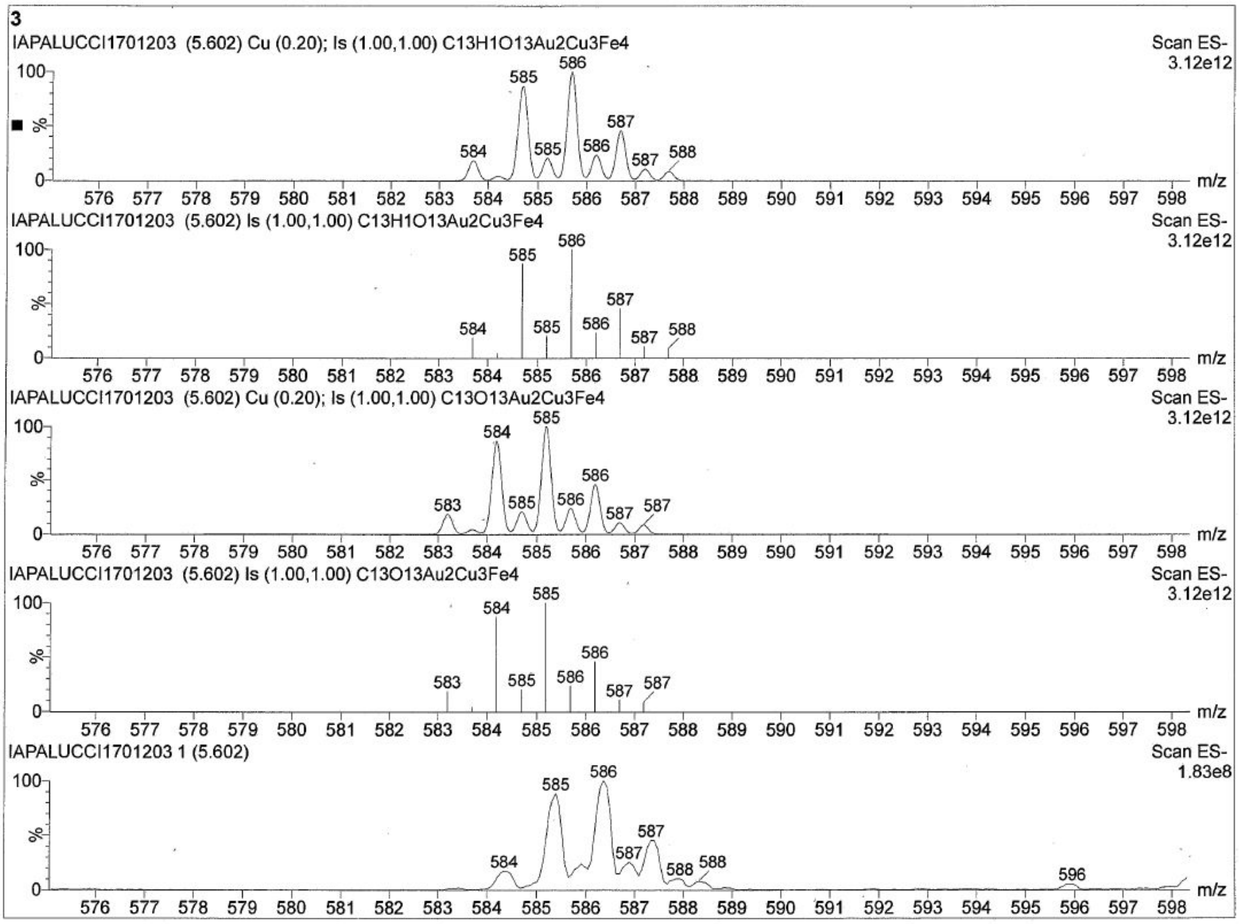

Figure S27

Isotopic pattern of the peak at m/z 628 of the ESI-MS spectrum in CH_3CN (ES–) of $[NEt_4]_3[Au_{2.48}Cu_{2.52}Fe_4(CO)_{16}]$. Upper trace: calculated isotopic patter for $[HAu_2Cu_3Fe_4(CO)_{16}]^{2-}$. Middle trace: calculated isotopic patter for $[Au_2Cu_3Fe_4(CO)_{16}]^{2-}$. Lower trace: experimental isotopic pattern.

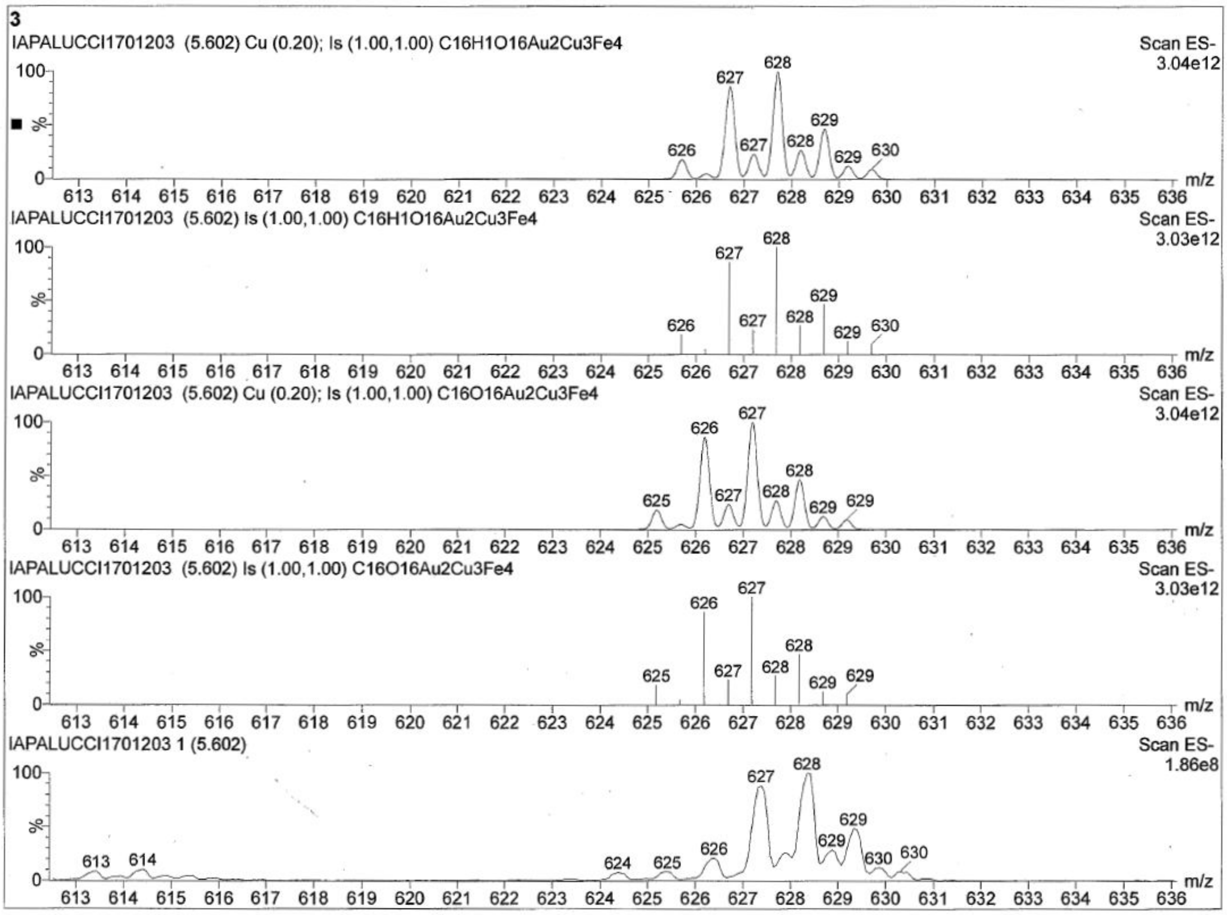

Figure S28

Isotopic pattern of the peak at m/z 638 of the ESI-MS spectrum in CH_3CN (ES–) of $[NEt_4]_3[Au_{2.48}Cu_{2.52}Fe_4(CO)_{16}]$. Upper trace: calculated isotopic patter for $[HAu_3Cu_2Fe_4(CO)_{12}]^{2-}$. Middle trace: calculated isotopic patter for $[Au_3Cu_2Fe_4(CO)_{12}]^{2-}$. Lower trace: experimental isotopic pattern.

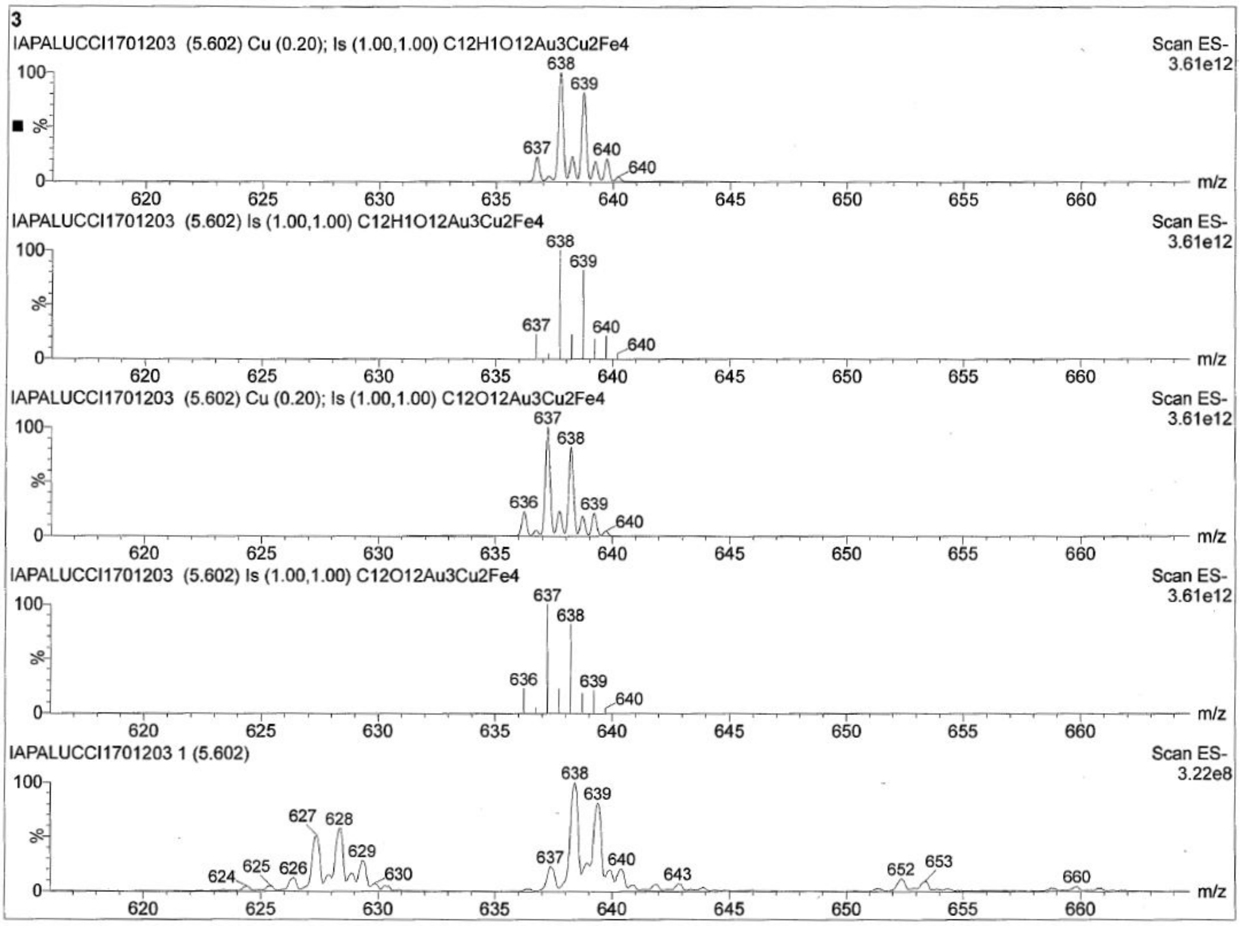

Figure S29

Isotopic pattern of the peak at m/z 694 of the ESI-MS spectrum in CH_3CN (ES–) of $[NEt_4]_3[Au_{2.48}Cu_{2.52}Fe_4(CO)_{16}]$. Upper trace: calculated isotopic patter for $[HAu_3Cu_2Fe_4(CO)_{16}]^{2-}$. Middle trace: calculated isotopic patter for $[Au_3Cu_2Fe_4(CO)_{16}]^{2-}$. Lower trace: experimental isotopic pattern.

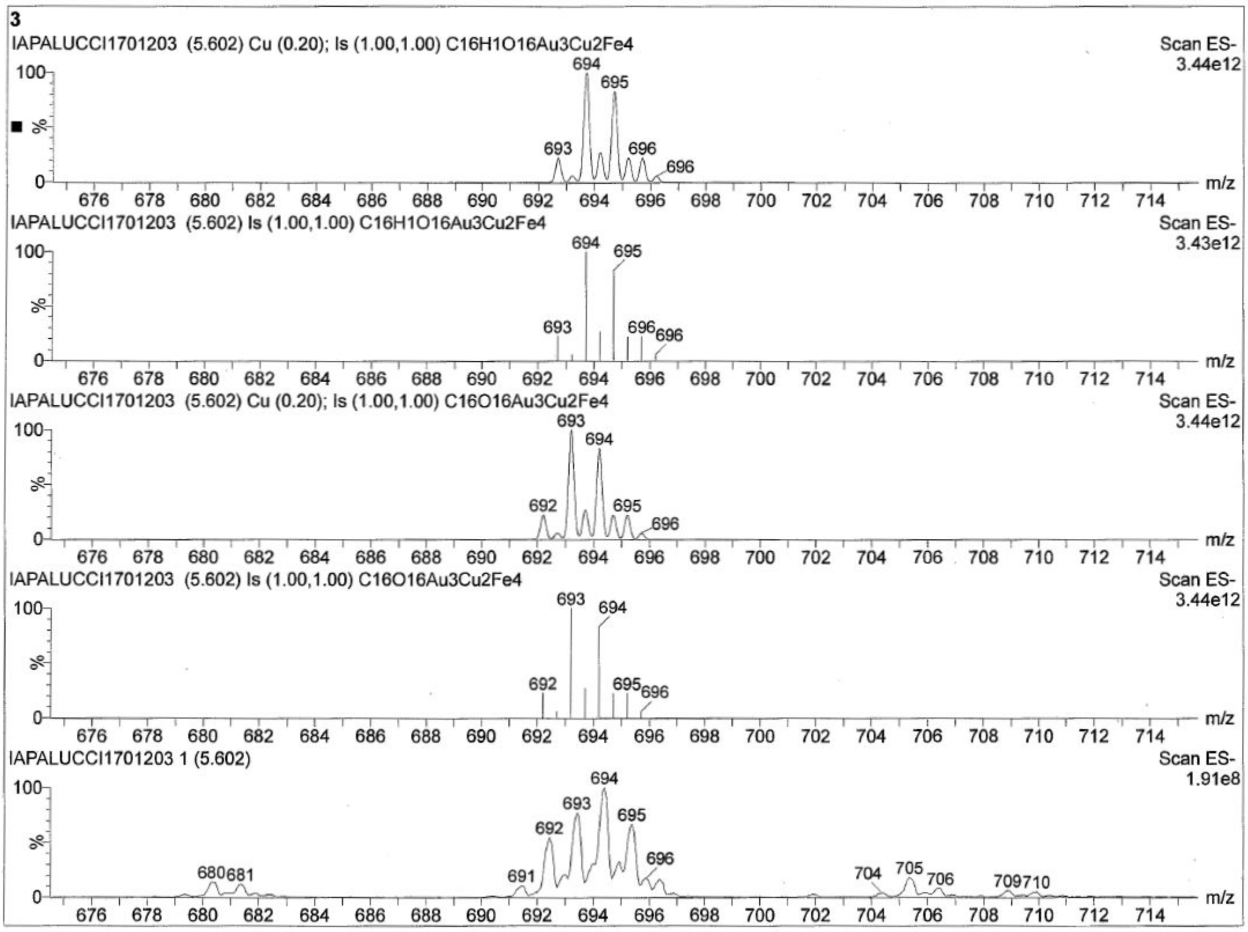

Figure S30

ESI-MS spectrum in CH_3CN (ES–) of $[NEt_4]_3[Au_{4.62}Cu_{0.38}Fe_4(CO)_{16}]$.

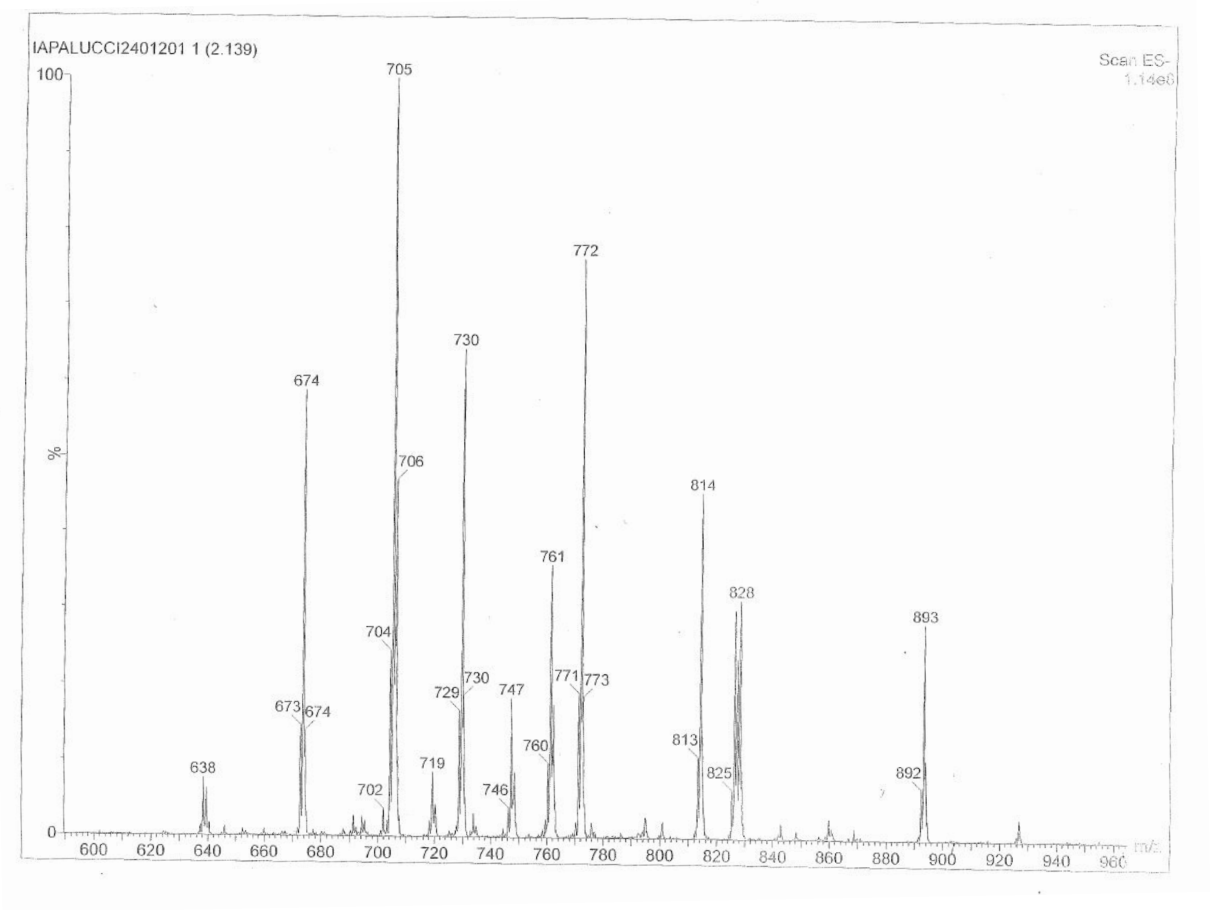

Table S9

Peak assignment of the ESI-MS spectrum (ES–) of $[NEt_4]_3[Au_{4.62}Cu_{0.38}Fe_4(CO)_{16}]$.

m/z	Relative intensity	Ion	Code
893	30	$\{[Au_5Fe_4(CO)_{16}][NEt_4]\}^{2-}$	Au_5+NEt_4
828	30	$[HAu_5Fe_4(CO)_{16}]^{2-}$	Au_5
826	30	$\{[Au_4CuFe_4(CO)_{16}][NEt_4]\}^{2-}$	Au_4Cu+NEt_4
814	45	$[HAu_5Fe_4(CO)_{15}]^{2-}$	Au_5(-1CO)
800	2	$[HAu_5Fe_4(CO)_{14}]^{2-}$	Au_5(-2CO)
772	80	$[HAu_5Fe_4(CO)_{12}]^{2-}$	Au_5(-4CO)
761	40	$[HAu_4CuFe_4(CO)_{16}]^{2-}$	Au_4Cu
747	20	$[HAu_4CuFe_4(CO)_{15}]^{2-}$	Au_4Cu(-1CO)
730	70	$[HAu_4CuFe_4(CO)_{14}]^{2-}$	Au_4Cu(-2CO)
719	10	$[HAu_4CuFe_4(CO)_{13}]^{2-}$	Au_4Cu(-3CO)
705	100	$[HAu_4CuFe_4(CO)_{12}]^{2-}$	Au_4Cu(-4CO)

Figure S31

Isotopic pattern of the peak at m/z 772 of the ESI-MS spectrum in CH_3CN (ES–) of $[NEt_4]_3[Au_{4.62}Cu_{0.38}Fe_4(CO)_{16}]$. Upper trace: calculated isotopic patter for $[HAu_5Fe_4(CO)_{12}]^{2-}$. Middle trace: calculated isotopic patter for $[Au_5Fe_4(CO)_{12}]^{2-}$. Lower trace: experimental isotopic pattern.

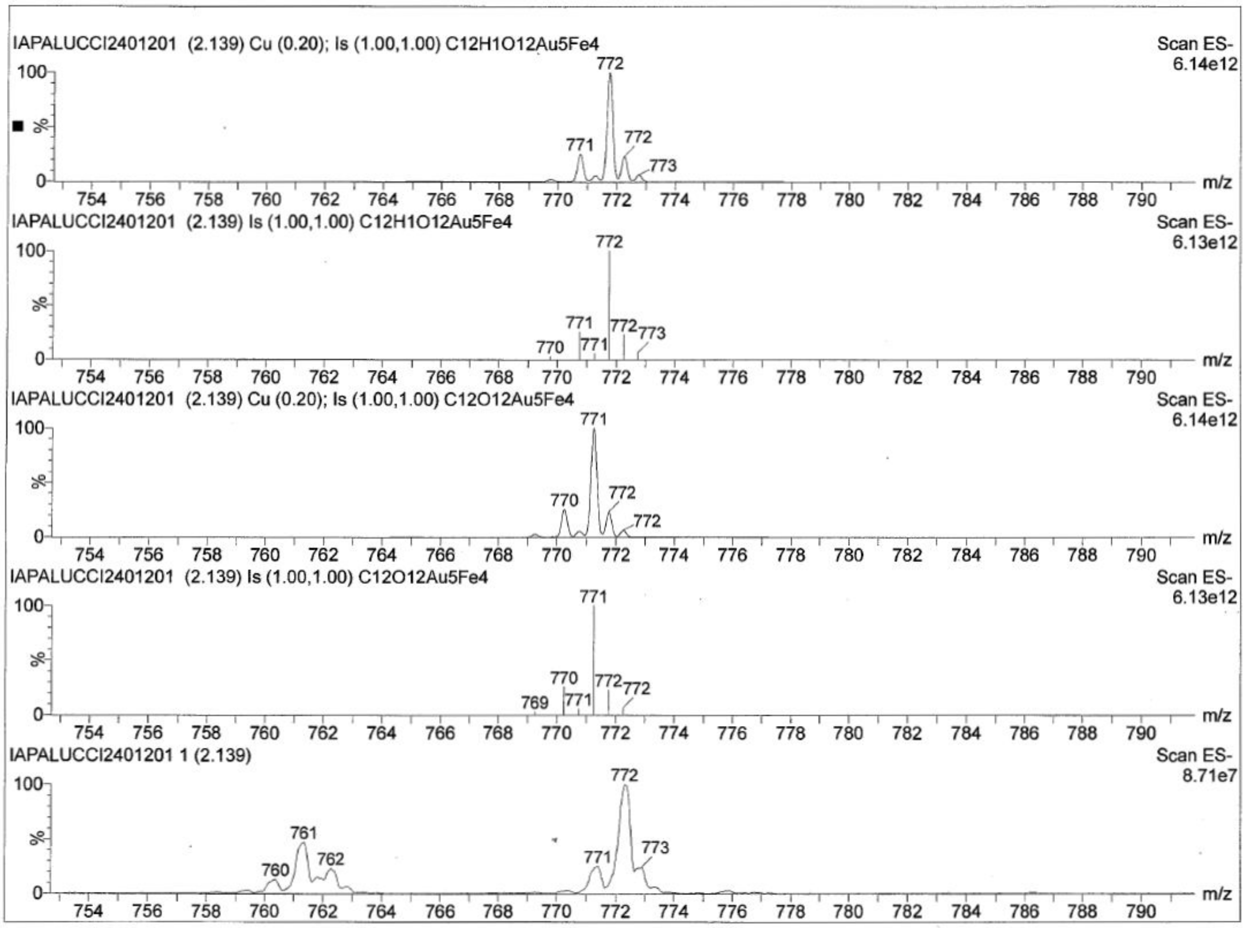

Figure S32

Isotopic pattern of the peak at m/z 705 of the ESI-MS spectrum in CH_3CN (ES–) of $[NEt_4]_3[Au_{4.62}Cu_{0.38}Fe_4(CO)_{16}]$. Upper trace: calculated isotopic patter for $[HAu_4CuFe_4(CO)_{12}]^{2-}$. Middle trace: calculated isotopic patter for $[Au_4CuFe_4(CO)_{12}]^{2-}$. Lower trace: experimental isotopic pattern.

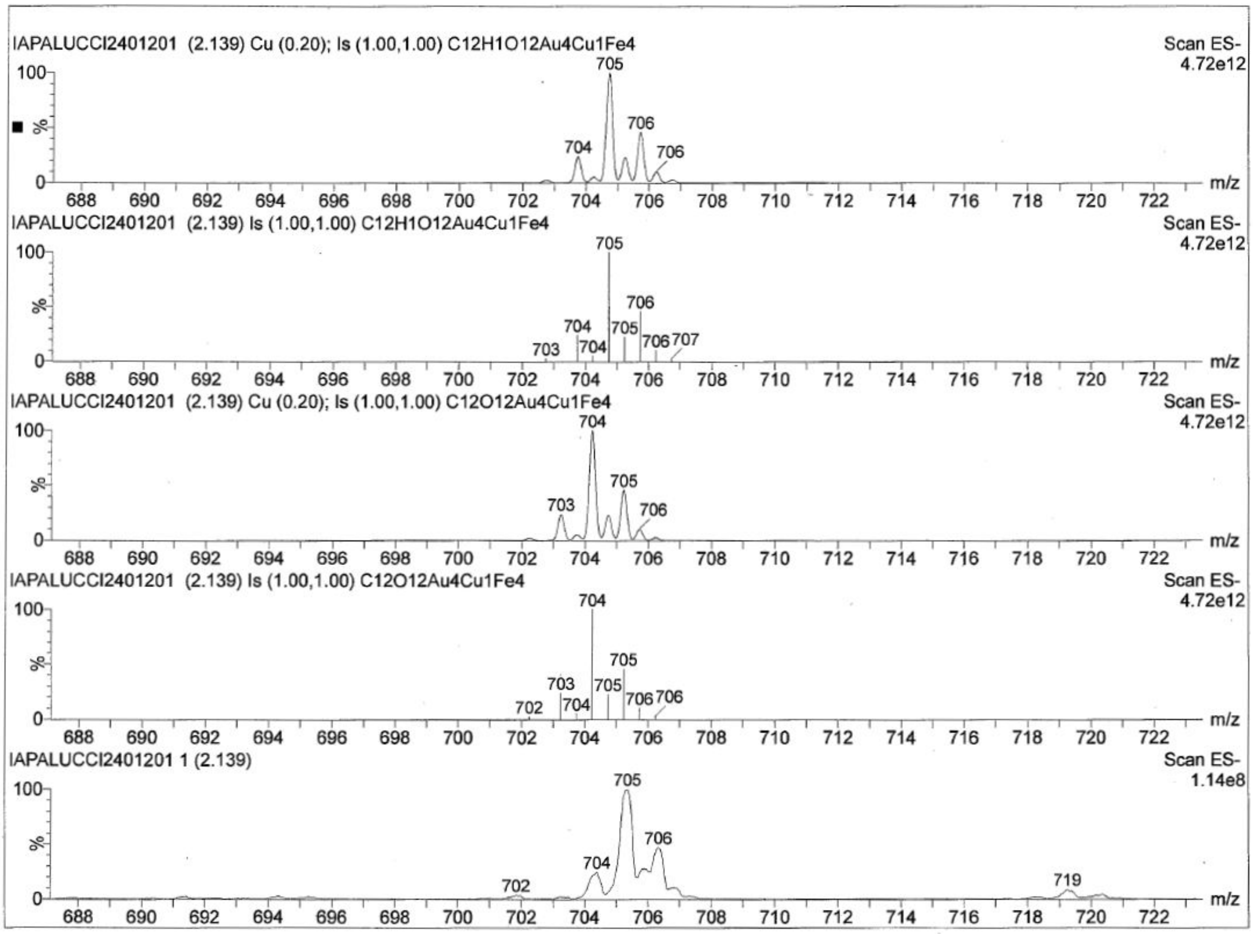

Figure S33

ESI-MS spectrum in CH_3CN (ES–) of $[NEt_4]_3[Au_{0.82}Ag_{4.18}Fe_4(CO)_{16}]$.

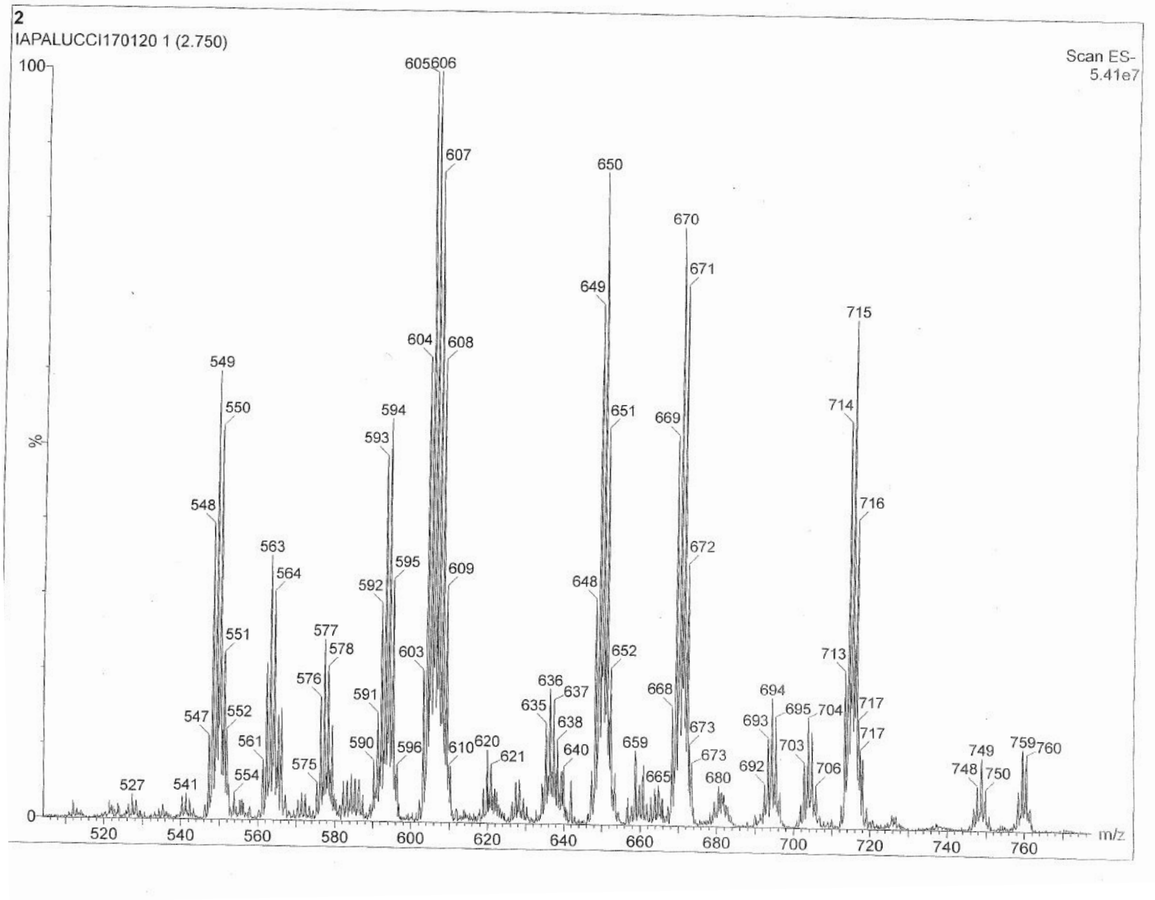

Table S10

Peak assignment of the ESI-MS spectrum (ES–) of $[NEt_4]_3[Au_{0.82}Ag_{4.18}Fe_4(CO)_{16}]$.

m/z	Relative intensity	Ion	Code
759	10	$\{[Au_2Ag_3Fe_4(CO)_{16}][NEt_4]\}^{2-}$	Au_2Ag_3+NEt_4
715	70	$\{[AuAg_4Fe_4(CO)_{16}][NEt_4]\}^{2-}$	$AuAg_4$+NEt_4
694	15	$[HAu_2Ag_3Fe_4(CO)_{16}]^{2-}$	Au_2Ag_3
680	5	$[HAu_2Ag_3Fe_4(CO)_{15}]^{2-}$	Au_2Ag_3(-1CO)
670	80	$\{[Ag_5Fe_4(CO)_{16}][NEt_4]\}^{2-}$	Ag_5+NEt_4
668	15	$[HAu_2Ag_3Fe_4(CO)_{14}]^{2-}$	Au_2Ag_3(-2CO)
650	90	$[HAuAg_4Fe_4(CO)_{16}]^{2-}$	$AuAg_4$
636	20	$[HAuAg_4Fe_4(CO)_{15}]^{2-}$	$AuAg_4$(-1CO)
620	10	$[HAuAg_4Fe_4(CO)_{14}]^{2-}$	$AuAg_4$(-2CO)
608	60	$[HAuAg_4Fe_4(CO)_{13}]^{2-}$	$AuAg_4$(-3CO)
605	100	$[HAg_5Fe_4(CO)_{16}]^{2-}$	Ag_5
595	30	$[HAuAg_4Fe_4(CO)_{12}]^{2-}$	$AuAg_4$(-4CO)
594	50	$[HAg_5Fe_4(CO)_{15}]^{2-}$	Ag_5(-1CO)
577	25	$[HAg_5Fe_4(CO)_{14}]^{2-}$	Ag_5(-2CO)
563	35	$[HAg_5Fe_4(CO)_{13}]^{2-}$	Ag_5(-3CO)
549	60	$[HAg_5Fe_4(CO)_{12}]^{2-}$	Ag_5(-4CO)

Figure S34

Isotopic pattern of the peak at m/z 549 of the ESI-MS spectrum in CH_3CN (ES–) of $[NEt_4]_3[Au_{0.82}Ag_{4.18}Fe_4(CO)_{16}]$. Upper trace: calculated isotopic patter for $[HAg_5Fe_4(CO)_{12}]^{2-}$. Middle trace: calculated isotopic patter for $[Ag_5Fe_4(CO)_{12}]^{2-}$. Lower trace: experimental isotopic pattern.

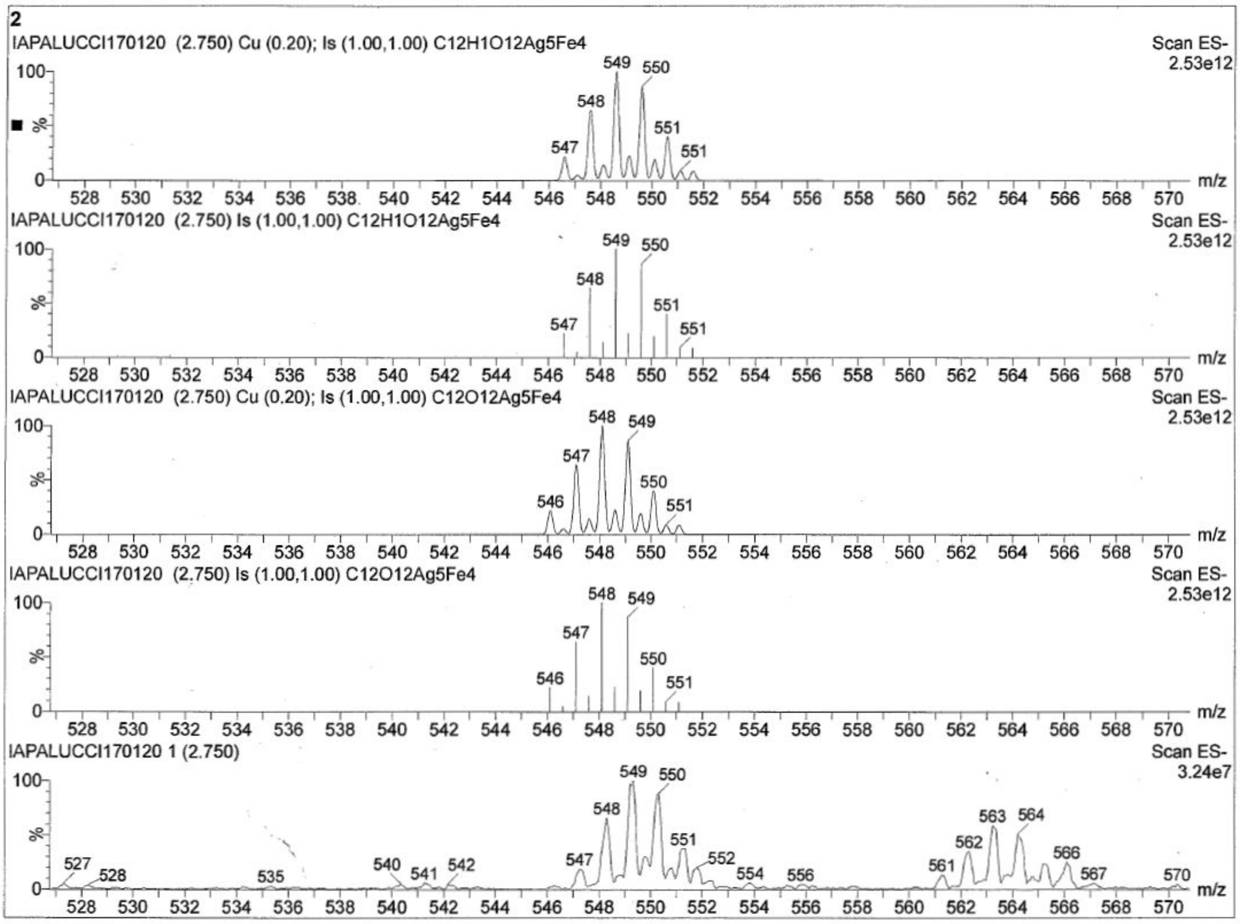

Figure S35

Isotopic pattern of the peak at m/z 605 of the ESI-MS spectrum in CH_3CN (ES–) of $[NEt_4]_3[Au_{0.82}Ag_{4.18}Fe_4(CO)_{16}]$. Upper trace: calculated isotopic patter for $[HAg_5Fe_4(CO)_{16}]^{2-}$. Middle trace: calculated isotopic patter for $[Ag_5Fe_4(CO)_{16}]^{2-}$. Lower trace: experimental isotopic pattern. There is a peak at m/z 608 that partially overlaps with the peak at m/z 605. Comparison of the calculated and experimental isotopic pattern confirms the presence of these two very close peaks.

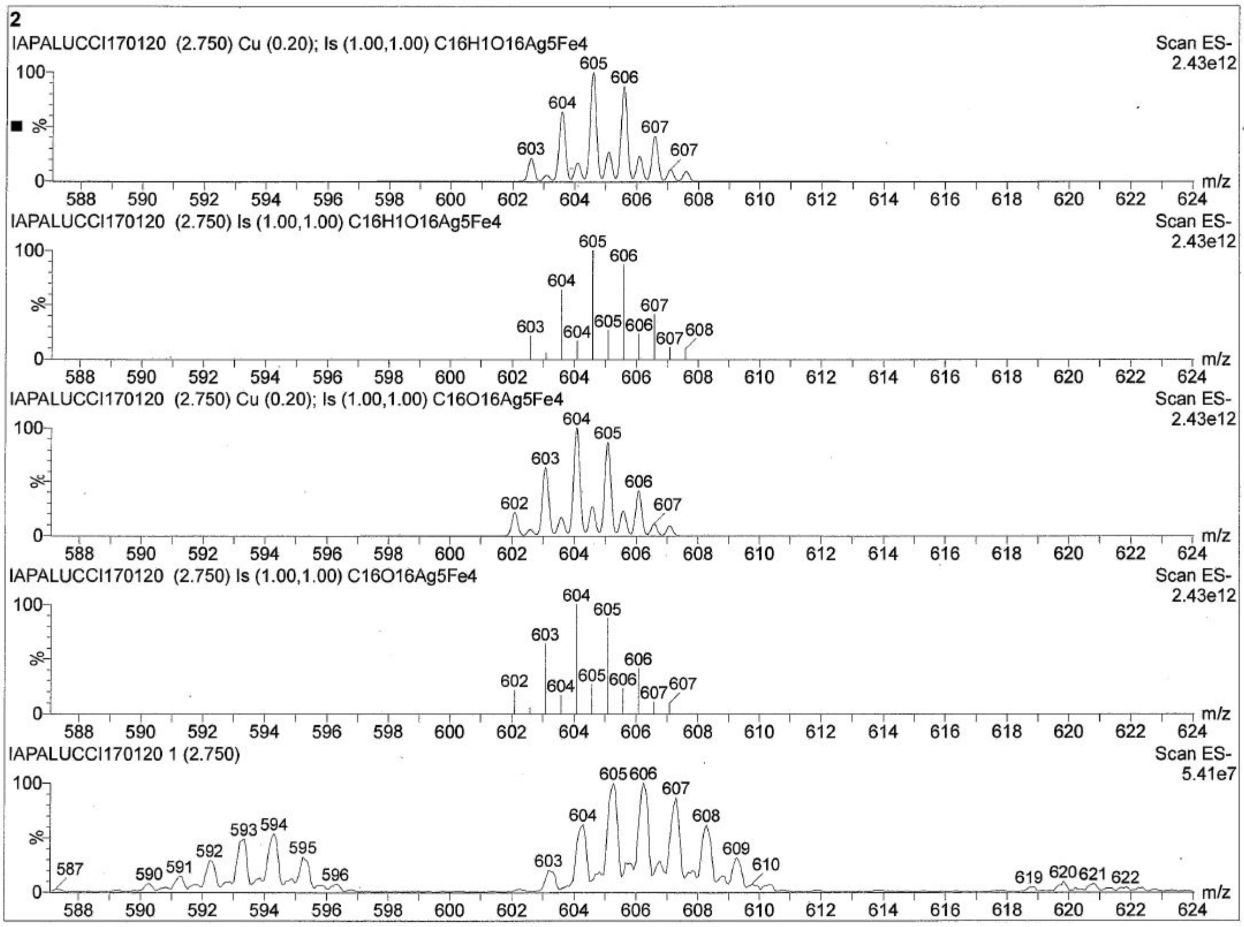

Figure S36

Isotopic pattern of the peak at m/z 608 of the ESI-MS spectrum in CH_3CN (ES–) of $[NEt_4]_3[Au_{0.82}Ag_{4.18}Fe_4(CO)_{16}]$. Upper trace: calculated isotopic patter for $[HAuAg_4Fe_4(CO)_{13}]^{2-}$. Middle trace: calculated isotopic patter for $[AuAg_4Fe_4(CO)_{13}]^{2-}$. Lower trace: experimental isotopic pattern. There is a peak at m/z 605 that partially overlaps with the peak at m/z 608. Comparison of the calculated and experimental isotopic pattern confirms the presence of these two very close peaks.

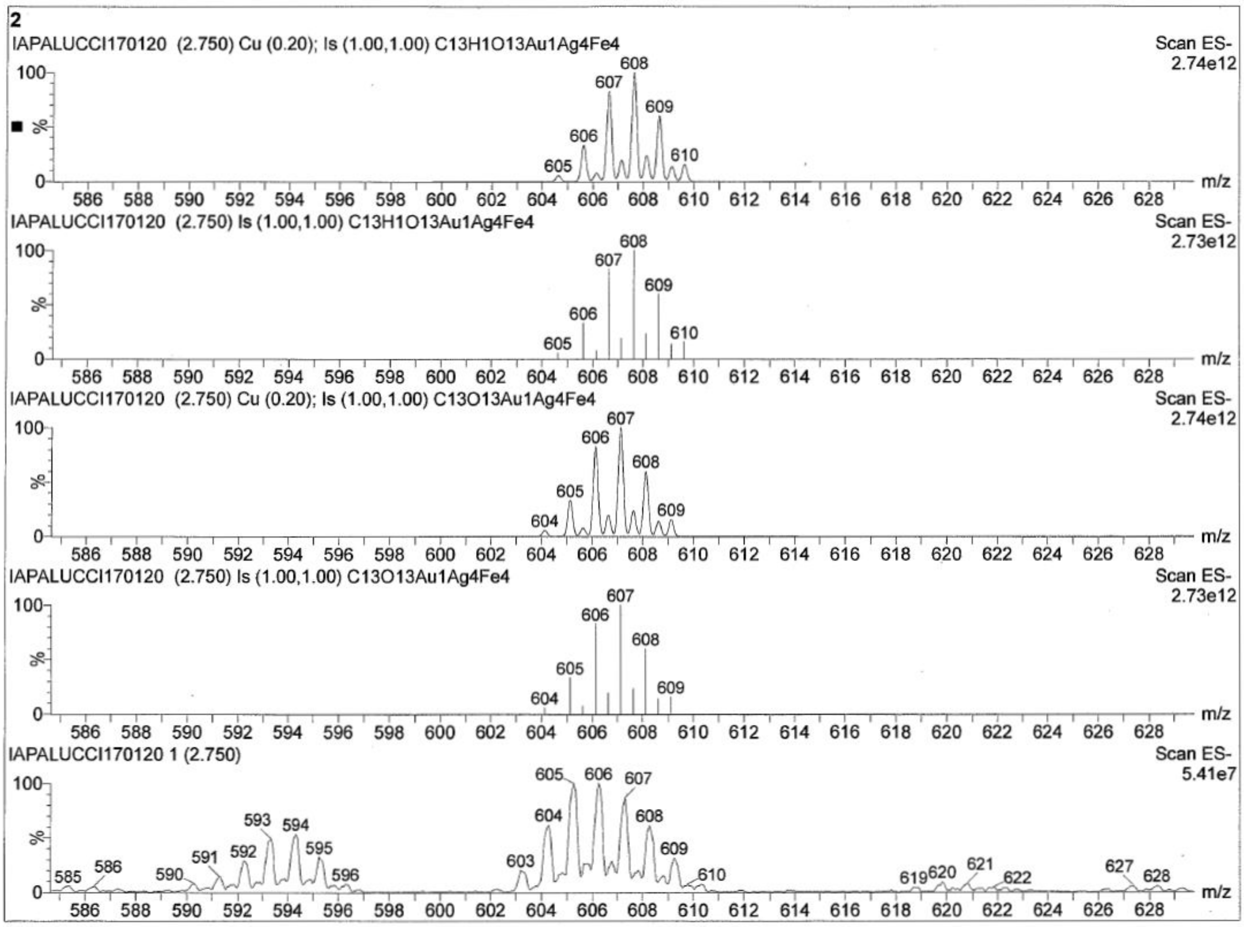

Figure S37

Isotopic pattern of the peak at m/z 650 of the ESI-MS spectrum in CH_3CN (ES–) of $[NEt_4]_3[Au_{0.82}Ag_{4.18}Fe_4(CO)_{16}]$. Upper trace: calculated isotopic patter for $[HAuAg_4Fe_4(CO)_{16}]^{2-}$. Middle trace: calculated isotopic patter for $[AuAg_4Fe_4(CO)_{16}]^{2-}$. Lower trace: experimental isotopic pattern.

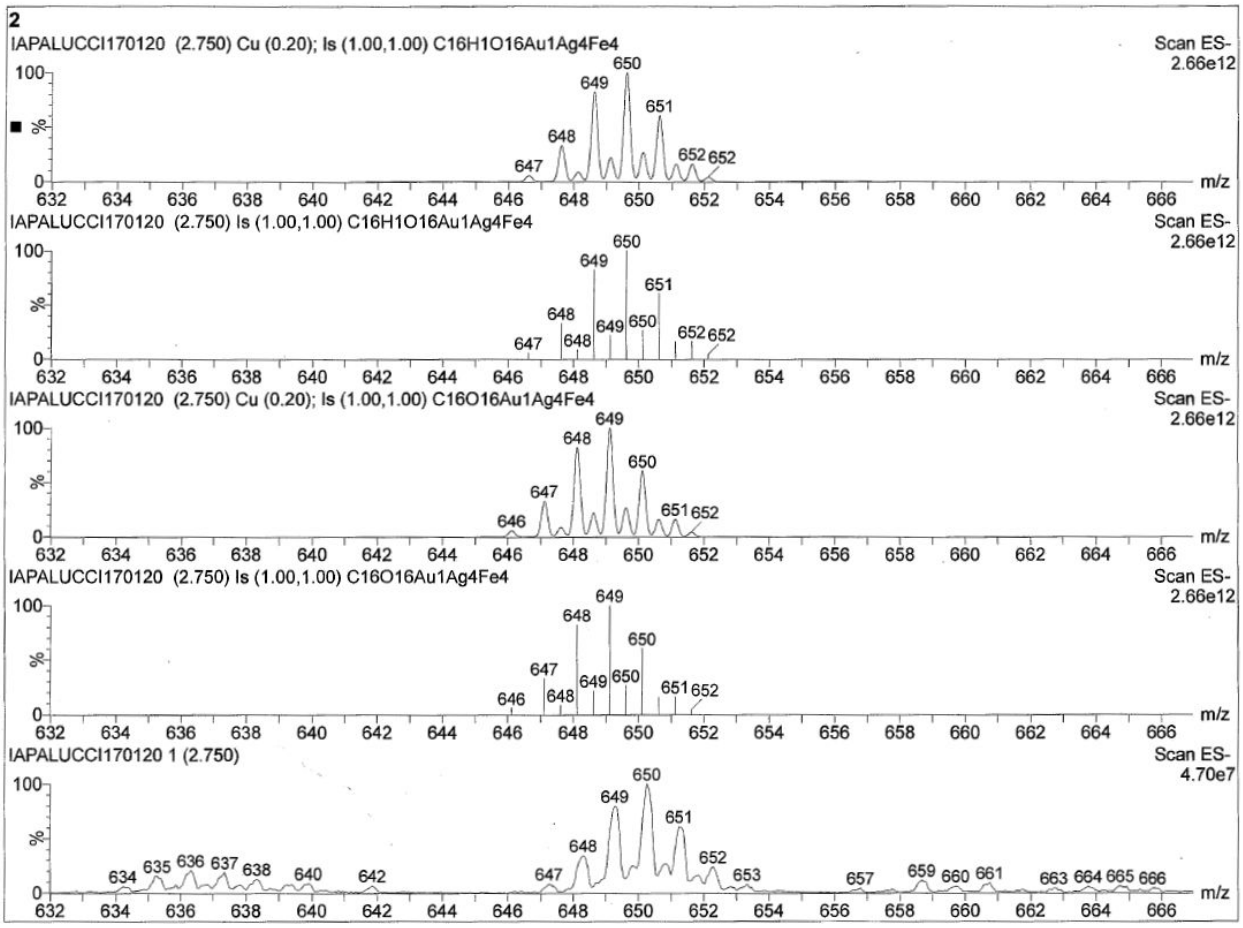

Figure S38

UV-visible absorption spectrum of $[NEt_4]_3[Cu_5Fe_4(CO)_{16}]$ in CH_3CN at 298 K (concentration 1.25×10^{-5} M).

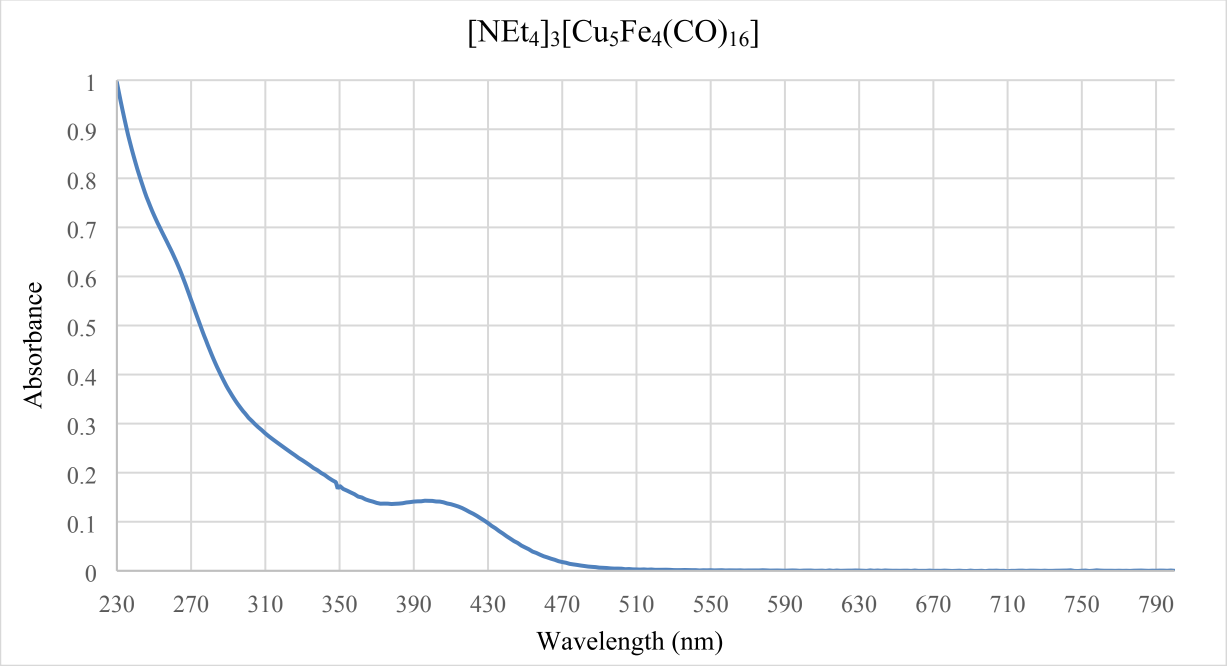

Figure S39

UV-visible absorption spectrum of $[NEt_4]_3[Ag_5Fe_4(CO)_{16}]$ in CH_3CN at 298 K (concentration 1.25×10^{-5} M).

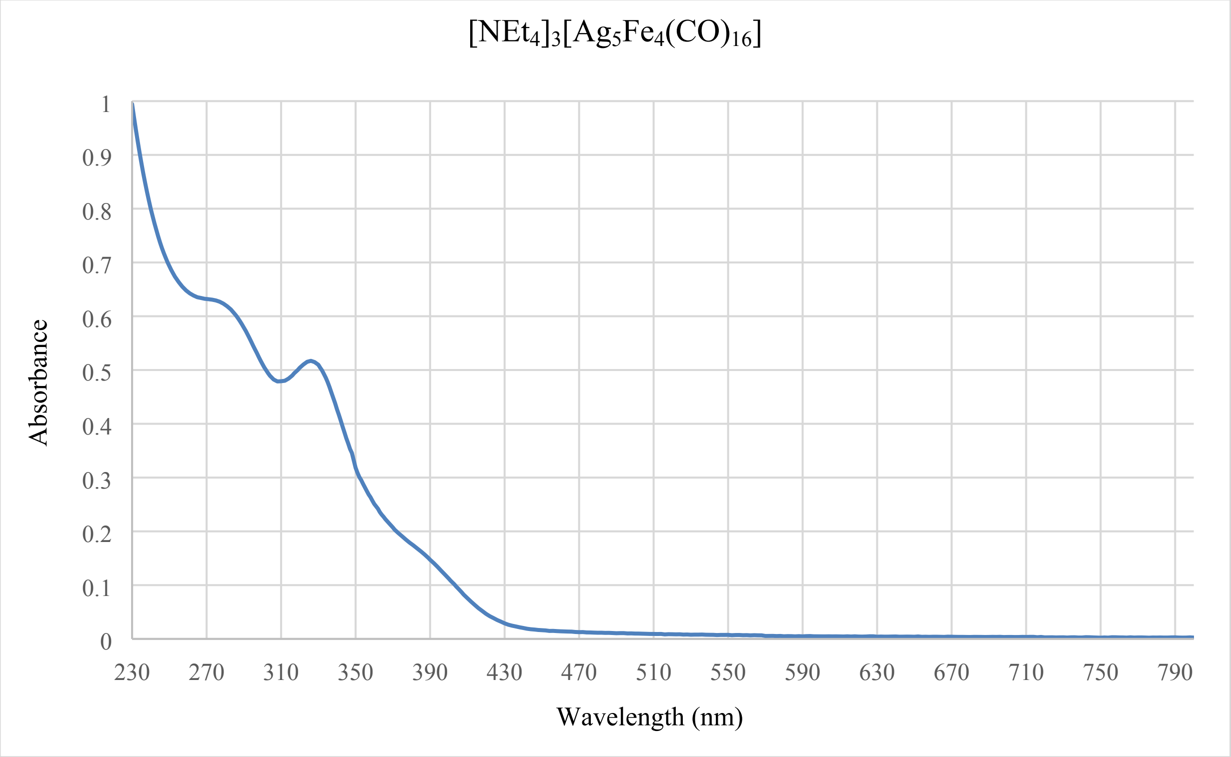

Figure S40

UV-visible absorption spectrum of $[NEt_4]_3[Au_{1.09}Cu_{3.91}Fe_4(CO)_{16}]$ in CH_3CN at 298 K (concentration 1.25×10^{-5} M).

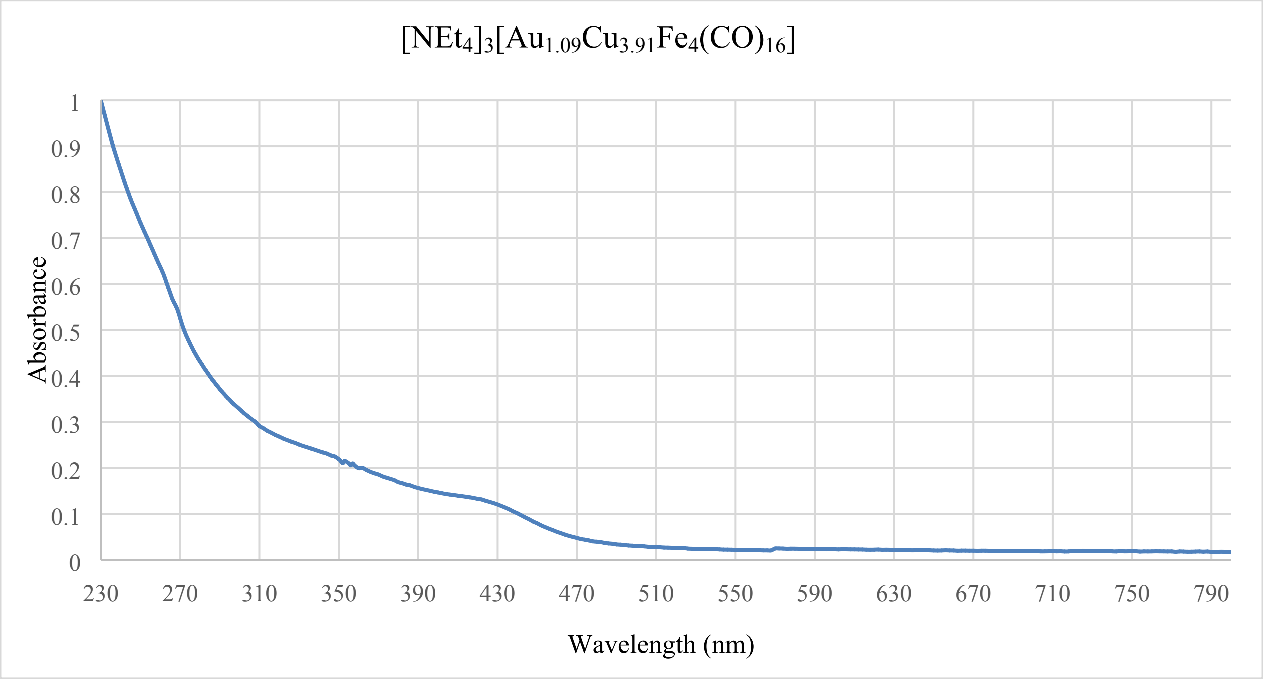

Figure S41

UV-visible absorption spectrum of $[NEt_4]_3[Au_{1.15}Cu_{3.82}Fe_4(CO)_{16}]$ in CH_3CN at 298 K (concentration 1.25×10^{-5} M).

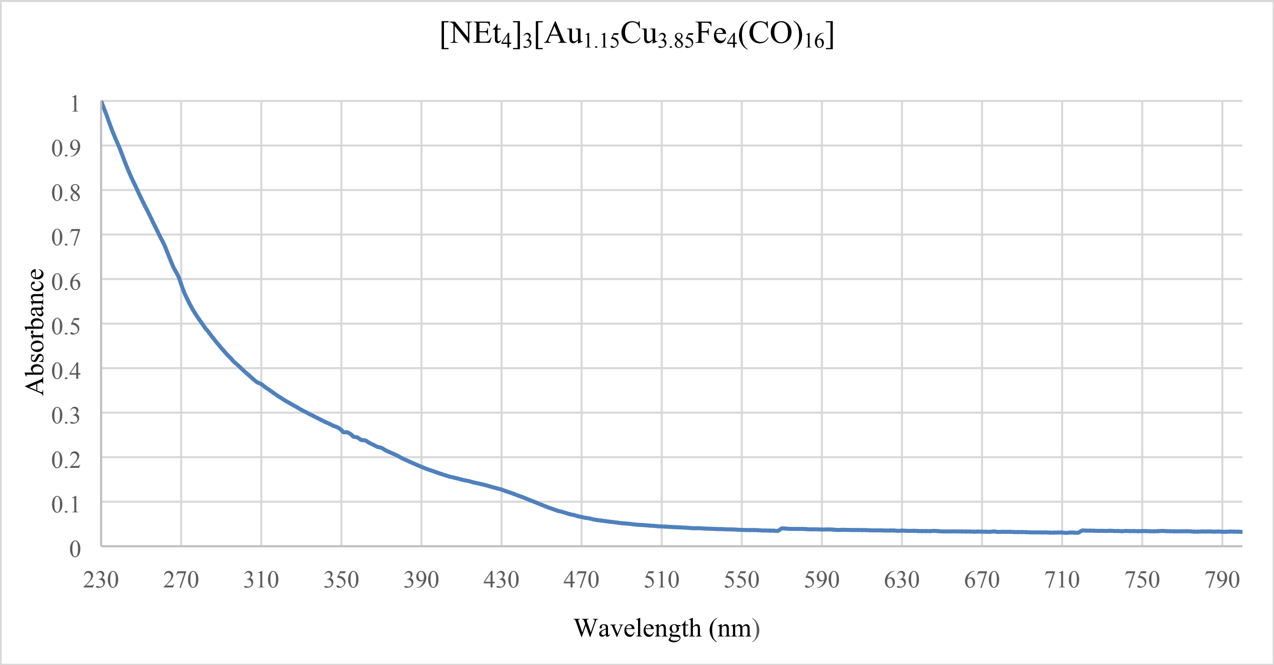

Figure S42

UV-visible absorption spectrum of $[NEt_4]_3[Au_{1.31}Cu_{3.69}Fe_4(CO)_{16}]$ in CH_3CN at 298 K (concentration 1.25×10^{-5} M).

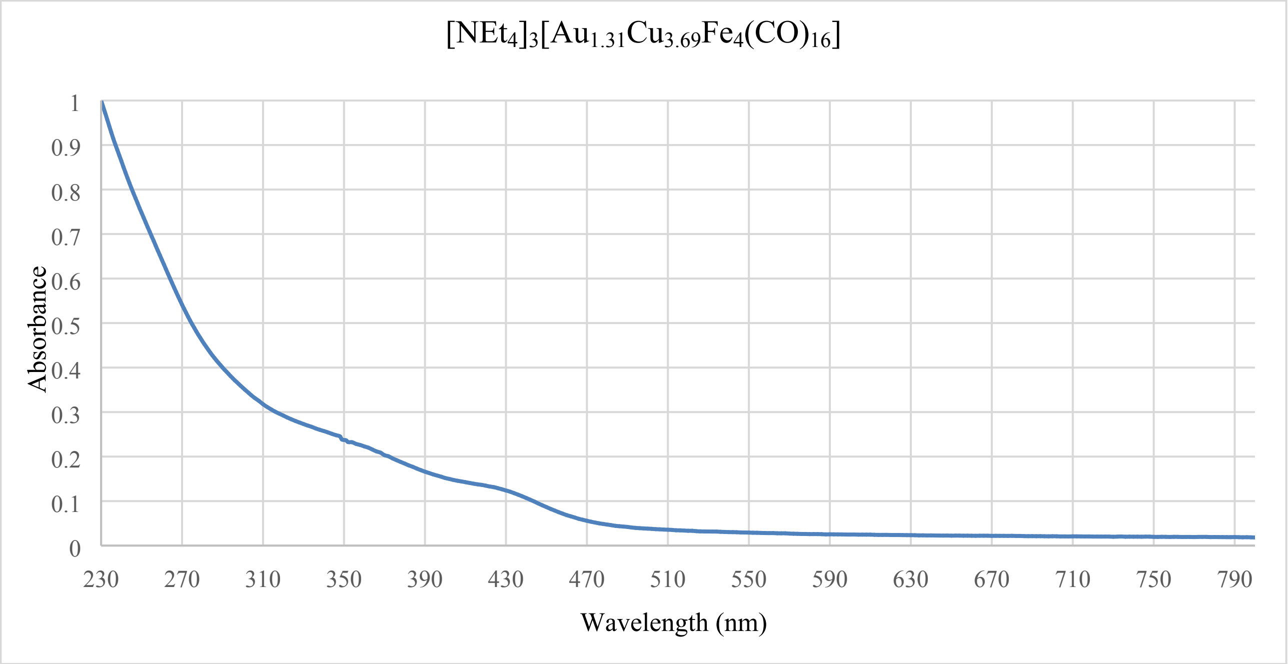

Figure S43

UV-visible absorption spectrum of $[NEt_4]_3[Au_{1.67}Cu_{3.33}Fe_4(CO)_{16}]$ in CH_3CN at 298 K (concentration 1.25×10^{-5} M).

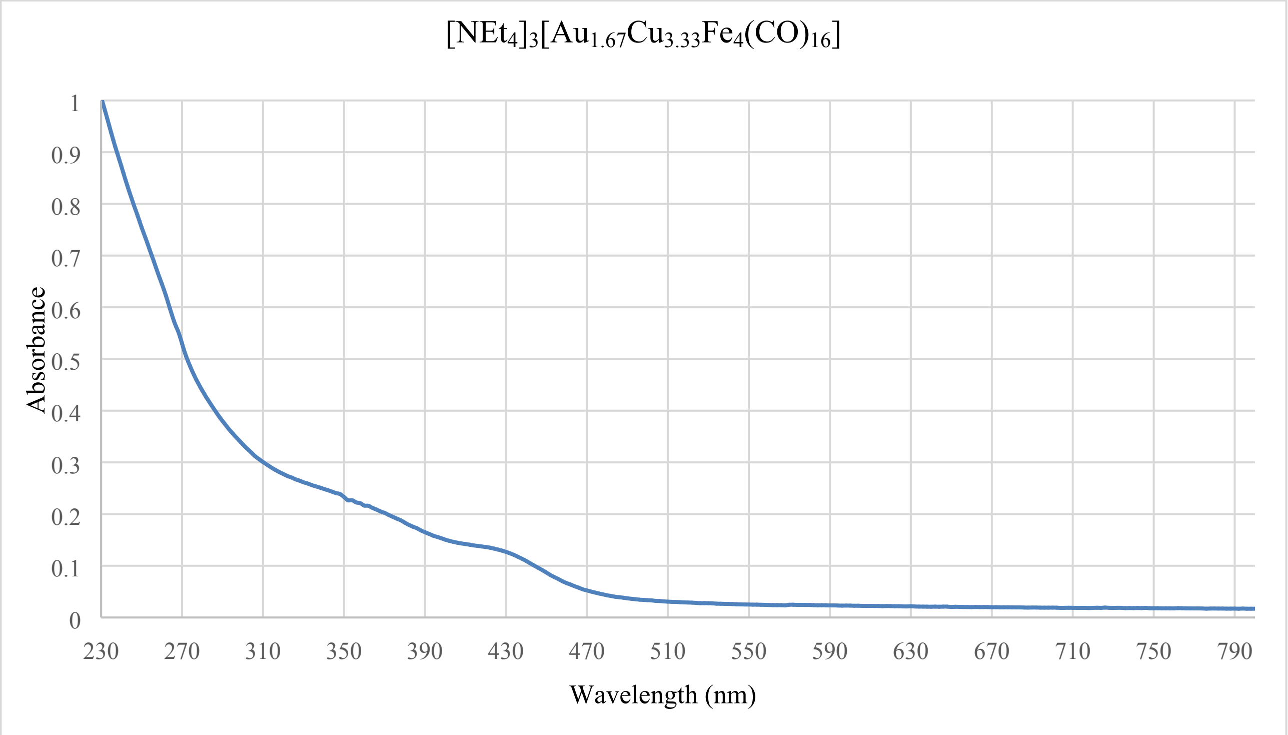

Figure S44

UV-visible absorption spectrum of $[NEt_4]_3[Au_{2.18}Cu_{2.82}Fe_4(CO)_{16}]$ in CH_3CN at 298 K (concentration 1.25×10^{-5} M).

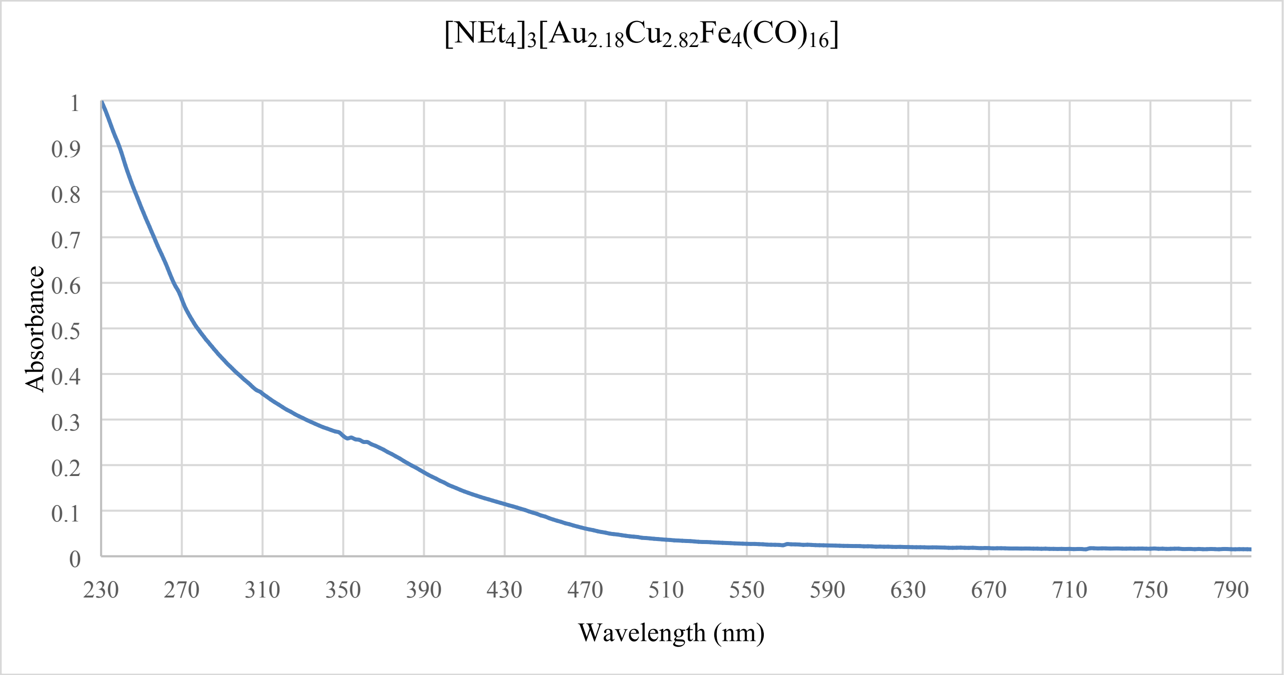

Figure S45

UV-visible absorption spectrum of $[NEt_4]_3[Au_{2.48}Cu_{2.52}Fe_4(CO)_{16}]$ in CH_3CN at 298 K (concentration 1.25×10^{-5} M).

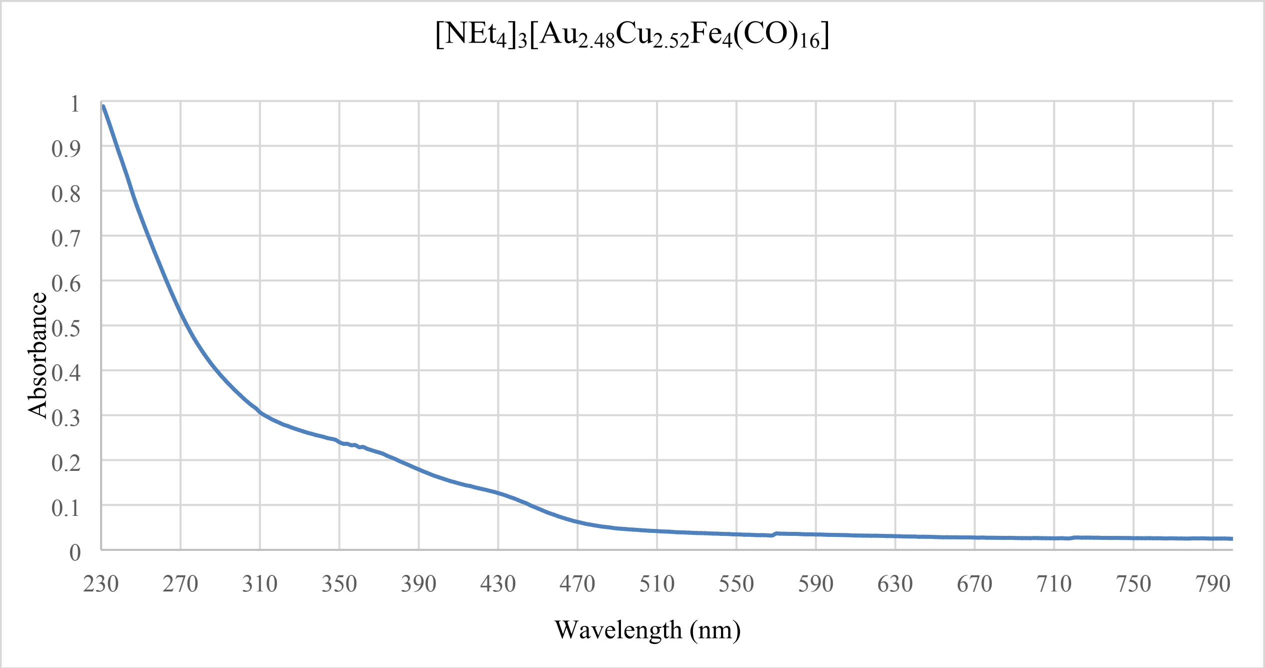

Figure S46

UV-visible absorption spectrum of [NEt$_4$]$_3$[Au$_{4.61}$Cu$_{0.39}$Fe$_4$(CO)$_{16}$] in CH$_3$CN at 298 K (concentration 1.25×10^{-5} M).

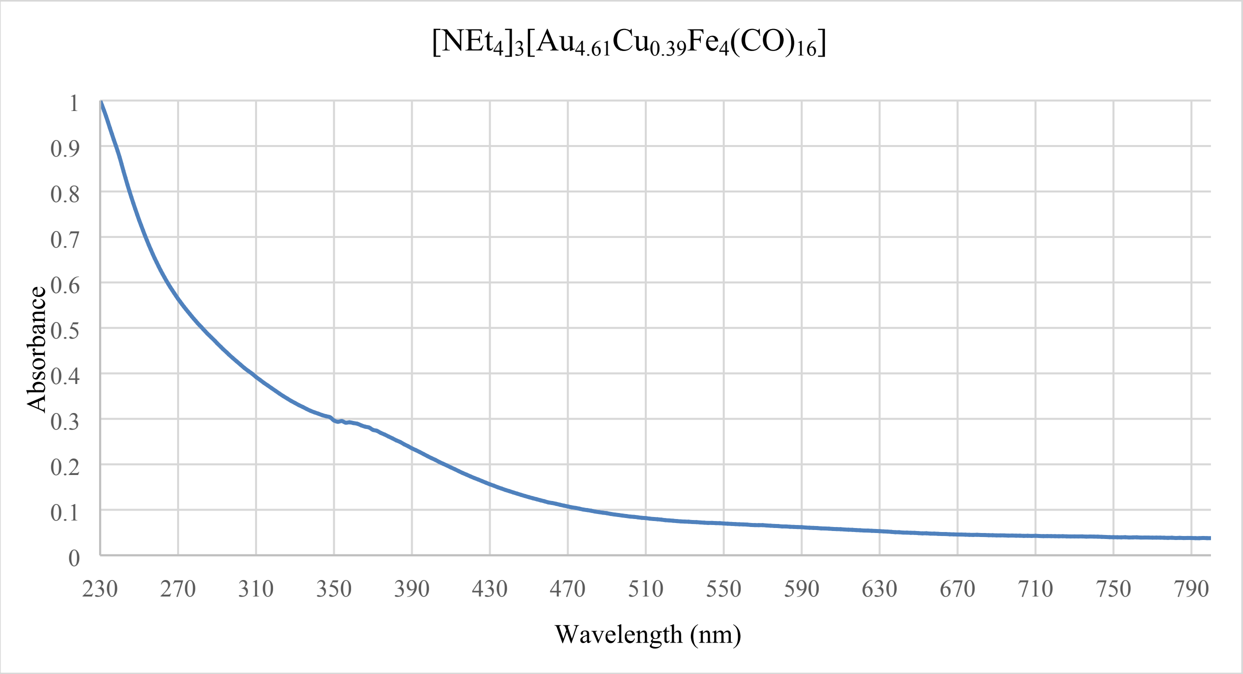

Figure S47

UV-visible absorption spectrum of [NEt$_4$]$_3$[Ag$_{1.02}$Cu$_{3.98}$Fe$_4$(CO)$_{16}$] in CH$_3$CN at 298 K (concentration 1.25×10^{-5} M).

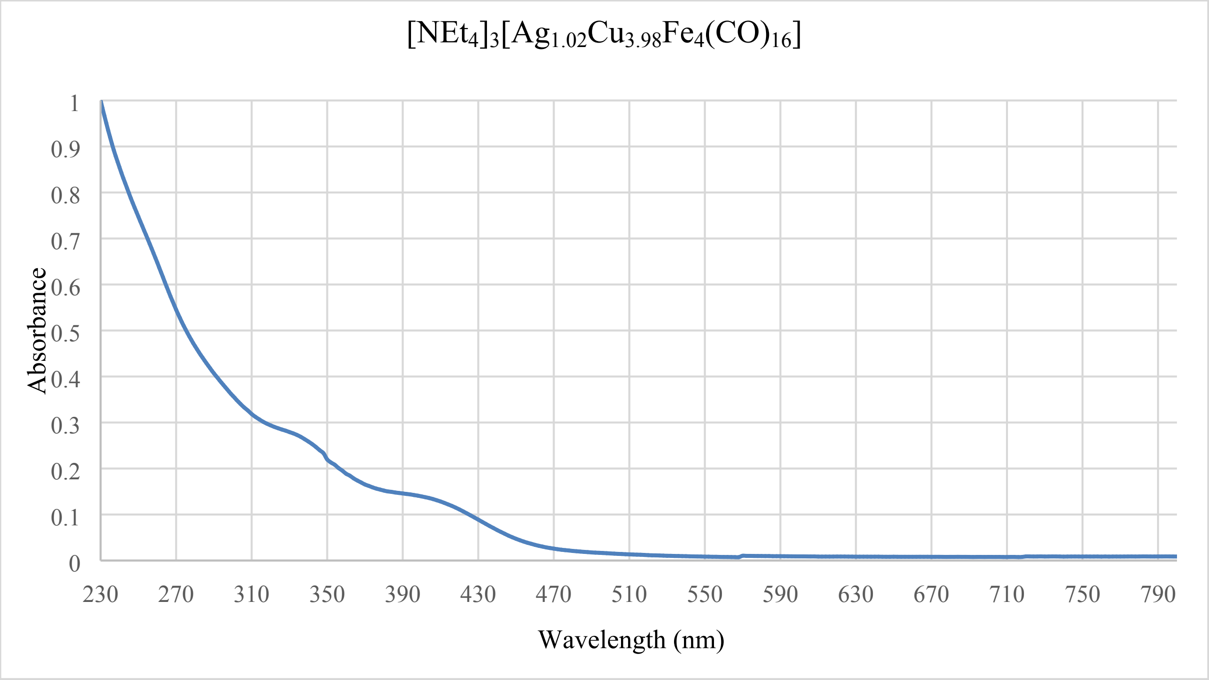

Figure S48

UV-visible absorption spectrum of $[NEt_4]_3[Ag_{3.31}Cu_{1.69}Fe_4(CO)_{16}]$ in CH_3CN at 298 K (concentration 1.25×10^{-5} M).

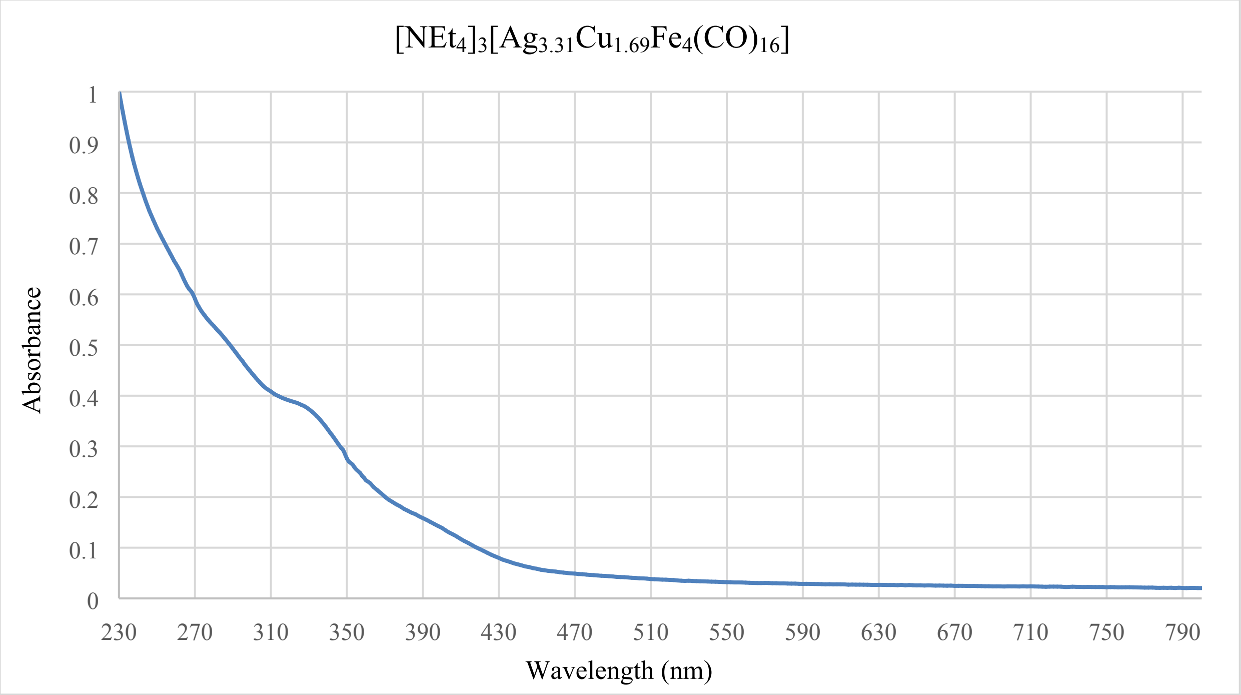

Figure S49

UV-visible absorption spectrum of $[NEt_4]_3[Ag_{3.45}Cu_{1.55}Fe_4(CO)_{16}]$ in CH_3CN at 298 K (concentration 1.25×10^{-5} M).

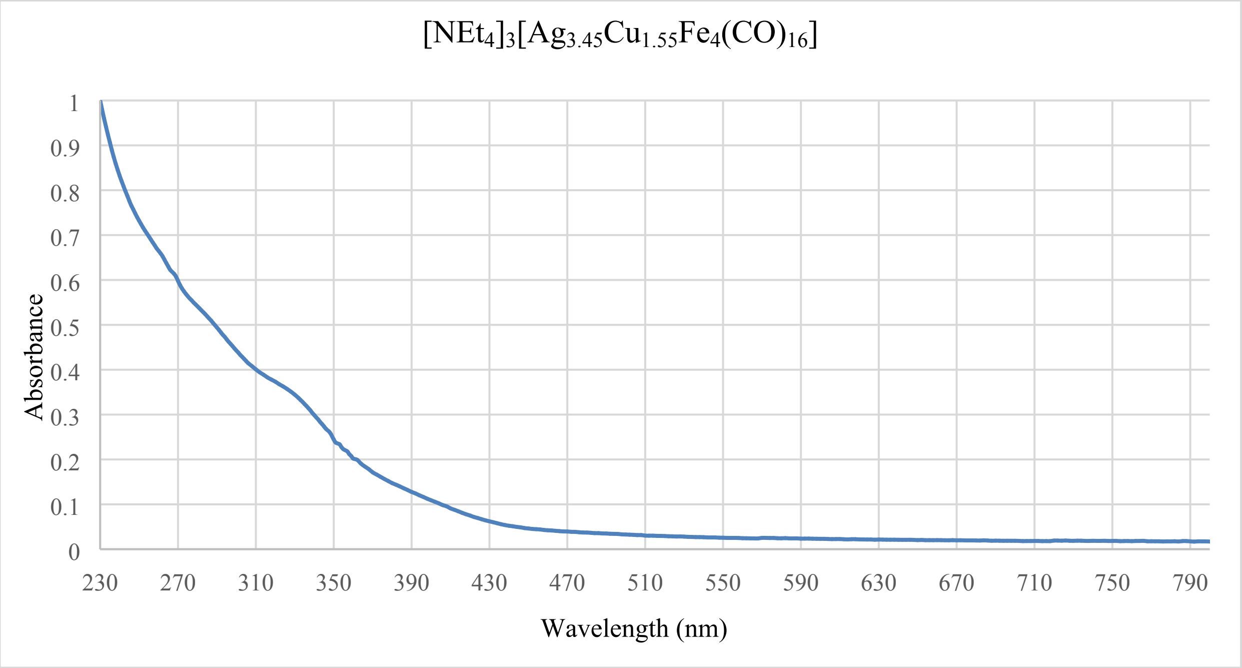

Figure S50

UV-visible absorption spectrum of $[NEt_4]_3[Ag_{4.25}Cu_{0.75}Fe_4(CO)_{16}]$ *in* CH_3CN *at 298 K (concentration* 1.25×10^{-5} *M).*

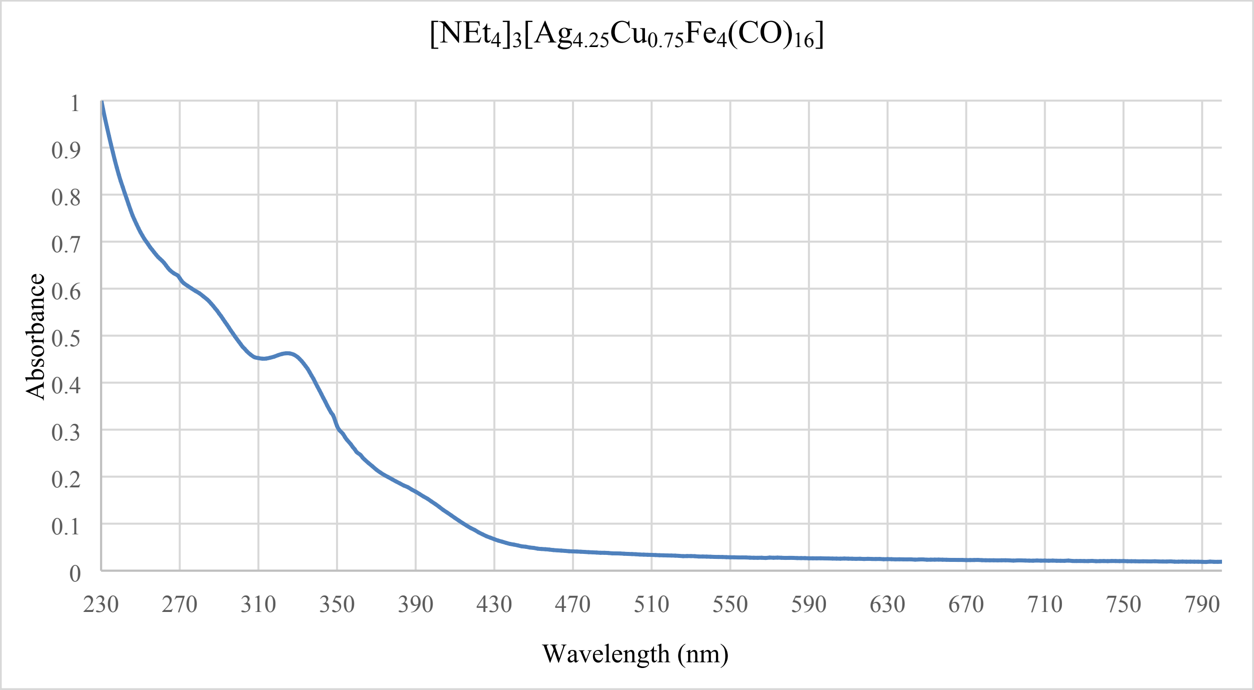

Figure S51

UV-visible absorption spectrum of $[NEt_4]_3[Ag_{4.37}Cu_{0.63}Fe_4(CO)_{16}]$ *in* CH_3CN *at 298 K (concentration* 1.25×10^{-5} *M).*

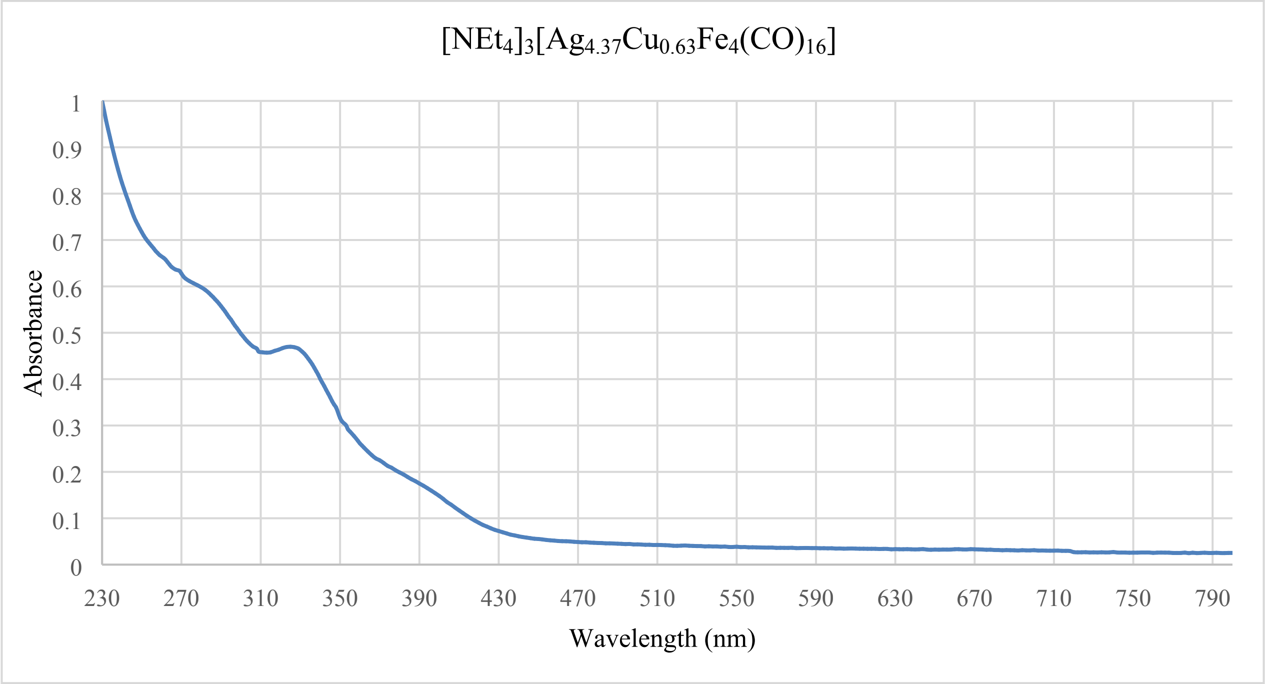

Figure S52

UV-visible absorption spectrum of $[NEt_4]_3[Ag_{5.00}Cu_{0.00}Fe_4(CO)_{16}]$ in CH_3CN at 298 K (concentration 1.25×10^{-5} M).

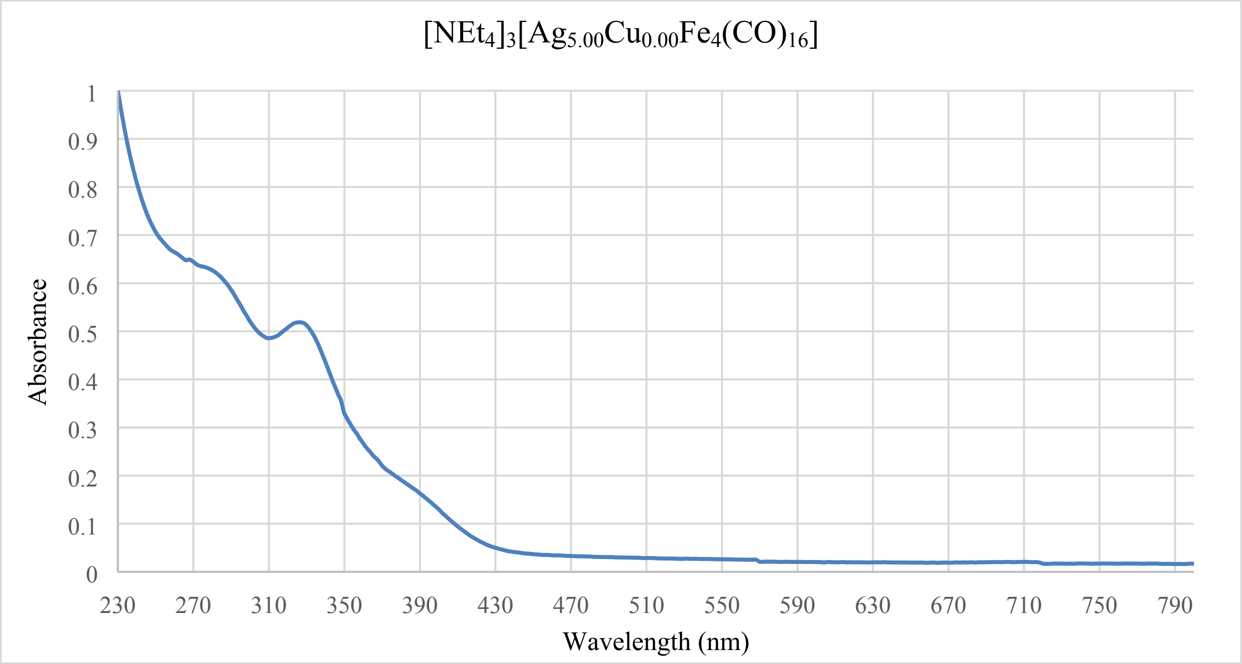

Figure S53

UV-visible absorption spectrum of $[NEt_4]_3[Au_{0.64}Ag_{4.36}Fe_4(CO)_{16}]$ in CH_3CN at 298 K (concentration 1.25×10^{-5} M).

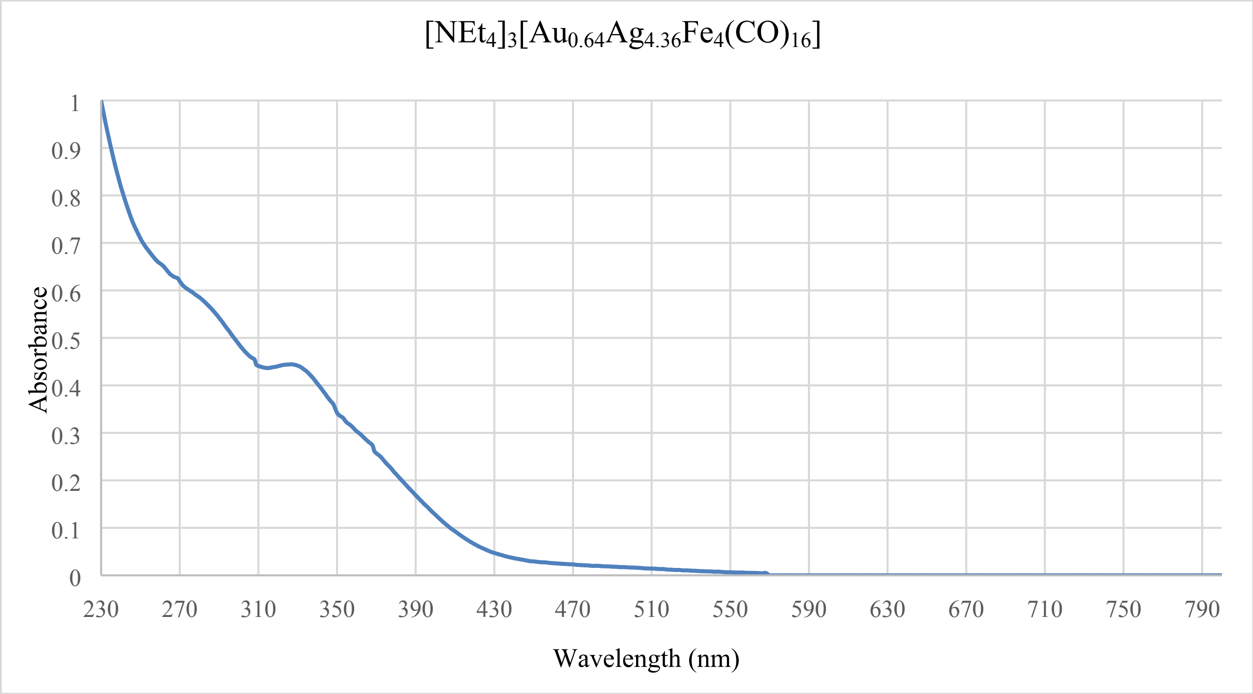

Figure S54

UV-visible absorption spectrum of $[NEt_4]_3[Au_{0.82}Ag_{4.18}Fe_4(CO)_{16}]$ in CH_3CN at 298 K (concentration 1.25×10^{-5} M).

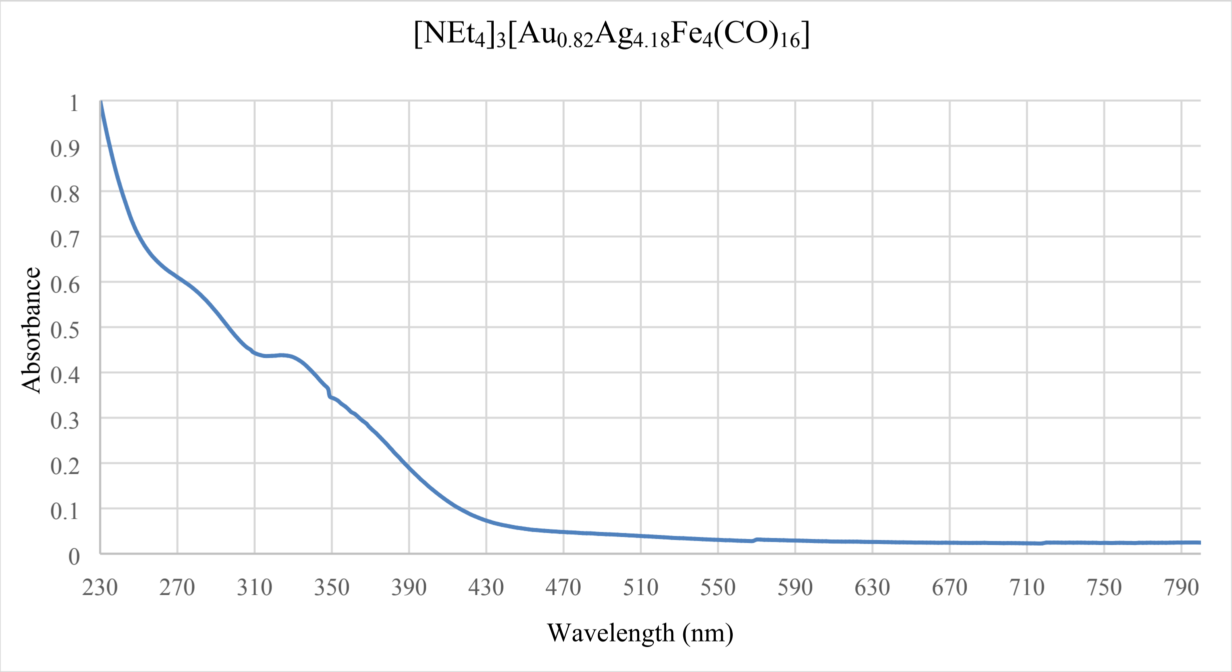

Table S11

UV-visible absorptions of $[NEt_4]_3[Au_xCu_{5-x}Fe_4(CO)_{16}]$ (x = 0-5) in CH_3CN solution (concentration 1.25×10^{-5} M). Weaker absorptions are given in parentheses. $\mathcal{E}$ (cm^{-1} M^{-1}) in red.

Entry*	Composition		Main absorption bands or shoulders (nm) with extinction coefficients		
	Au	Cu			
**	0.00	5.00	266 4.75×10^4	(331) 1.78×10^4	408 1.10×10^4
20	1.09	3.91	269 4.32×10^4	-	428 9.89×10^3
12	1.15	3.85	269 4.81×10^4	(348) 2.14×10^4	430 1.02×10^4
13	1.31	3.69	-	(347) 1.97×10^4	430 9.91×10^3
14	1.67	3.33	(268) 4.43×10^4	355 1.80×10^4	430 1.02×10^4
16	2.18	2.82	(268) 4.67×10^4	365 1.95×10^4	-
15	2.48	2.52	-	364 1.80×10^4	(435) 9.53×10^3
19	4.62	0.38	(269) 4.56×10^4	367 2.25×10^4	428 1.28×10^4
***	5.00	0.00	-	-	-

* See Table 2 in the main text. Entries are listed in order of increasing Au content.
** Prepared in accordance to Ref. 32 in the main text.
*** $[NEt_4]_3[Au_5Fe_4(CO)_{16}]$ is irreversibly oxidized after dilution and, therefore, its UV-vis spectrum has not been recorded

Table S12

UV-visible absorptions of $[NEt_4]_3[Ag_xCu_{5-x}Fe_4(CO)_{16}]$ (x = 0-5) in CH_3CN solution (concentration 1.25×10^{-5} M). Weaker absorptions are given in parentheses. $\mathcal{E}$ (cm^{-1} M^{-1}) in red.

Entry*	Composition		Main absorption bands or shoulders (nm) with extinction coefficients		
	Ag	Cu			
**	0.00	5.00	266<br>4.75×10^4	(331)<br>1.78×10^4	408<br>1.10×10^4
1	1.02	3.98	-	337<br>2.13×10^4	407<br>1.06×10^4
10	3.31	1.69	(286)<br>4.09×10^4	333<br>2.91×10^4	399<br>1.13×10^4
11	3.45	1.55	(290)<br>3.95×10^4	331<br>2.72×10^4	-
3	4.25	0.75	287<br>4.51×10^4	328<br>3.68×10^4	(399)<br>1.16×10^4
8	4.37	0.63	287<br>4.58×10^4	330<br>3.70×10^4	(401)<br>1.16×10^4
2,6	5.00	0.00	283<br>4.95×10^4	326<br>4.15×10^4	(399)<br>1.06×10^4

* See Table 1 in the main text. Entries are listed in order of increasing Ag content.
** Prepared in accordance to Ref. 32 in the main text.

Table S13

UV-visible absorptions of $[NEt_4]_3[Au_xAg_{5-x}Fe_4(CO)_{16}]$ (x = 0-5) in CH_3CN solution (concentration 1.25×10^{-5} M). Weaker absorptions are given in parentheses. $\mathcal{E}$ (cm^{-1} M^{-1}) in red.

Entry*	Composition		Main absorption bands or shoulders (nm) with extinction coefficients		
	Au	Ag			
2,6	0.00	5.00	283<br>4.90×10^4	326<br>4.14×10^4	(399)<br>9.27×10^3
21	0.64	4.36	290<br>4.34×10^4	328<br>3.55×10^4	-
22	0.82	4.18	284<br>4.50×10^4	330<br>3.47×10^4	-
**	5.00	0.00	-	-	-

* See Table 3 in the main text. Entries are listed in order of increasing Au content.
** $[NEt_4]_3[Au_5Fe_4(CO)_{16}]$ is irreversibly oxidized after dilution and, therefore, its UV-vis spectrum has not been recorded

Table S14

Selected properties of (3,-1) b.c.p. in the isomers of $[Cu_4AgFe_4(CO)_{16}]^{3-}$. All quantities in a.u.

bond	ρ	*V*	*E*	$\nabla^2\rho$
**Cu_4Ag-is1**				
Ag1-Cu6	0.036	-0.037	-0.009	0.074
Ag1-Cu9	0.036	-0.037	-0.009	0.074
Ag1-Cu7	0.036	-0.037	-0.009	0.074
Ag1-Cu8	0.036	-0.037	-0.009	0.074
Cu6-Fe5	0.046	-0.043	-0.016	0.040
Cu7-Fe5	0.046	-0.043	-0.016	0.041
Cu8-Fe3	0.046	-0.043	-0.016	0.040
Cu9-Fe3	0.046	-0.043	-0.016	0.041
Cu6-Fe4	0.052	-0.050	-0.017	0.062
Cu9-Fe4	0.052	-0.050	-0.017	0.062
Cu8-Fe2	0.052	-0.050	-0.017	0.062
Cu7-Fe2	0.052	-0.050	0.017	0.062
Ag1-Fe3	0.044	-0.040	-0.013	0.058
Ag1-Fe5	0.044	-0.040	-0.013	0.058
Cu6-Cu9	No (3,-1) b.c.p.			
Cu7-Cu8	No (3,-1) b.c.p.			
**Cu_4Ag-is2**				
Cu1-Ag6	0.033	-0.032	-0.008	0.064
Cu1-Cu9	No (3,-1) b.c.p.			
Cu1-Cu7	0.034	-0.034	0.012	0.040
Cu1-Cu8	0.035	-0.035	-0.012	0.040
Ag6-Fe5	0.044	-0.038	-0.013	0.049
Cu7-Fe5	0.049	-0.046	-0.016	0.041
Cu9-Fe3	0.049	-0.046	-0.017	0.051
Cu8-Fe3	0.049	-0.046	-0.017	0.050
Ag6-Fe4	0.049	-0.044	-0.014	0.062
Cu9-Fe4	0.050	-0.049	-0.017	0.062
Cu7-Fe2	0.052	-0.051	-0.018	0.063
Cu8-Fe2	0.052	-0.051	-0.018	0.064
Cu1-Fe3	0.047	-0.044	-0.016	0.048
Cu1-Fe5	0.045	-0.042	-0.016	0.043
Ag6-Cu9	No (3,-1) b.c.p.			
Cu7-Cu8	No (3,-1) b.c.p.			

Cu_4Ag-is1

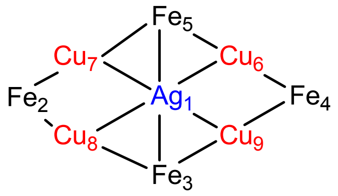

Cu_4Ag-is2

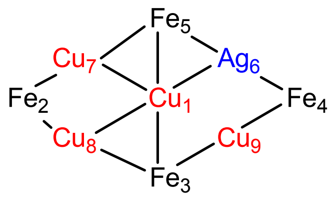

Table S15

Selected properties of (3,-1) b.c.p. in the isomers of $[Cu_3Ag_2Fe_4(CO)_{16}]^{3-}$. All quantities in a.u.

Cu_3Ag_2-is1

bond	ρ	*V*	*E*	$\nabla^2\rho$
Ag1-Ag6	0.034	-0.035	-0.006	0.094
Ag1-Cu9	0.034	-0.034	-0.009	0.065
Ag1-Cu7	0.036	-0.037	-0.009	0.073
Ag1-Cu8	0.036	-0.037	-0.009	0.073
Ag6-Fe5	0.041	-0.035	-0.012	0.044
Cu7-Fe5	0.046	-0.042	-0.016	0.041
Cu9-Fe3	0.046	-0.043	-0.016	0.041
Cu8-Fe3	0.046	-0.043	-0.016	0.041
Ag6-Fe4	0.049	-0.045	-0.014	0.064
Cu9-Fe4	0.050	-0.049	-0.017	0.059
Cu7-Fe2	0.052	-0.051	-0.017	0.063
Cu8-Fe2	0.052	-0.050	-0.017	0.062
Ag1-Fe3	0.044	-0.039	-0.012	0.058
Ag1-Fe5	0.042	-0.037	-0.012	0.054
Ag6-Cu9	No (3,-1) b.c.p.			
Cu7-Cu8	No (3,-1) b.c.p.			

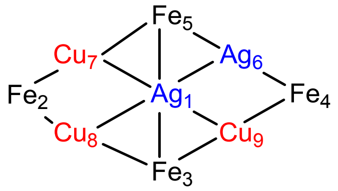

Cu_3Ag_2-is2

Cu1-Ag6	0.033	-0.032	-0.008	0,063
Cu1-Ag8	0.033	-0.032	-0.008	0,063
Cu1-Cu7	No (3,-1) b.c.p.			
Cu1-Cu9	No (3,-1) b.c.p.			
Cu7-Fe5	0.049	-0.046	-0.017	0.051
Cu9-Fe3	0.049	-0.046	-0.017	0.051
Ag6-Fe5	0.044	-0.038	-0.013	0.051
Ag8-Fe3	0.044	-0.038	-0.013	0.051
Cu9-Fe4	0.051	-0.050	-0.017	0.060
Cu7-Fe2	0.051	-0.050	-0.017	0.060
Ag6-Fe4	0.049	-0.045	-0.014	0.066
Ag8-Fe2	0.049	-0.045	-0.014	0.066
Cu1-Fe3	0.045	-0.042	-0.016	0.043
Cu1-Fe5	0.045	-0.042	-0.016	0.043
Ag6-Cu9	No (3,-1) b.c.p.			
Cu7-Ag8	No (3,-1) b.c.p.			

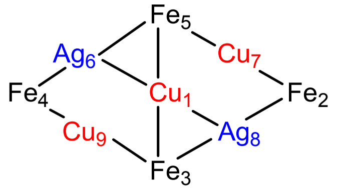

Cu_3Ag_2-is3

Cu1-Ag6	0.029	-0.026	-0.007	0.051
Cu1-Ag9	0.029	-0.027	-0.007	0.051
Cu1-Cu7	0.034	-0.033	-0.012	0.038
Cu1-Cu8	0.034	0.033	-0.012	0.038
Ag6-Fe5	0.044	-0.038	-0.013	0.051
Ag9-Fe3	0.044	-0.038	-0.013	0.051
Cu7-Fe5	0.049	-0.046	-0.017	0.051
Cu8-Fe3	0.049	-0.046	-0.017	0.051
Ag6-Fe4	0.047	-0.043	-0.014	0.061
Ag9-Fe4	0.047	-0.043	-0.014	0.061
Cu7-Fe2	0.052	-0.051	-0.018	0.064
Cu8-Fe2	0.052	-0.051	-0.018	0.064
Cu1-Fe3	0.045	-0.042	-0.016	0.043
Cu1-Fe5	0.045	-0.042	-0.016	0.043
Ag6-Ag9	0.030	-0.026	-0.004	0.073
Cu7-Cu8	No (3,-1) b.c.p.			

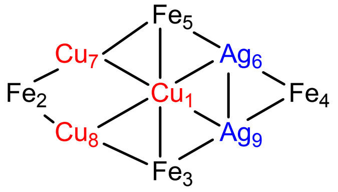

Cu_3Ag_2-is4

Cu1-Ag6	0.032	-0.031	-0.008	0.062
Cu1-Ag7	0.032	-0.031	-0.008	0.062
Cu1-Cu9	No (3,-1) b.c.p.			
Cu1-Cu8	No (3,-1) b.c.p.			
Ag6-Fe5	0.044	-0.038	-0.013	0.049
Ag7-Fe5	0.044	-0.038	-0.013	0.049
Cu9-Fe3	0.050	-0.047	-0.017	0.053
Cu8-Fe3	0.050	-0.047	-0.017	0.054
Ag6-Fe4	0.049	-0.044	-0.014	0.062
Ag7-Fe2	0.049	-0.044	-0.014	0.063
Cu9-Fe4	0.051	-0.050	-0.017	0.062
Cu8-Fe2	0.051	-0.050	-0.017	0.062
Cu1-Fe3	0.046	-0.044	-0.016	0.048
Cu1-Fe5	0.043	-0.040	-0.015	0.038
Ag6-Cu9	No (3,-1) b.c.p.			
Ag7-Cu8	No (3,-1) b.c.p.			

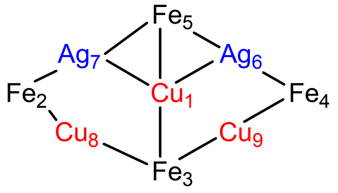

Table S16

Selected properties of (3,-1) b.c.p. in the isomers of $[Cu_2Ag_3Fe_4(CO)_{16}]^{3-}$. All quantities in a.u.

Cu_2Ag_3-is1

bond	ρ	V	E	$\nabla^2\rho$
Cu1-Cu6	No (3,-1) b.c.p.			
Cu1-Ag9	0.032	-0.032	-0.008	0.063
Cu1-Ag8	0.029	-0.027	-0.007	0.052
Cu1-Ag7	0.029	-0.026	-0.007	0.051
Cu6-Fe5	0.049	-0.047	-0.017	0.055
Ag7-Fe5	0.045	-0.039	-0.013	0.053
Ag9-Fe3	0.044	-0.039	-0.013	0.052
Ag8-Fe3	0.044	-0.039	-0.013	0.052
Cu6-Fe4	0.051	-0.050	-0.017	0.061
Ag9-Fe4	0.049	-0.045	-0.014	0.066
Ag8-Fe2	0.048	0.044	-0.014	0.063
Ag7-Fe2	0.048	-0.044	-0.014	0.063
Cu1-Fe3	0.042	-0.039	-0.015	0.037
Cu1-Fe5	0.044	-0.042	-0.015	0.043
Cu6-Ag9	No (3,-1) b.c.p.			
Ag7-Ag8	0.031	-0.028	-0.004	0.078

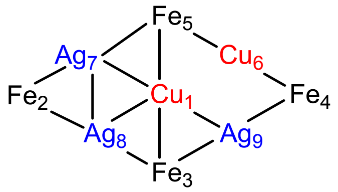

Cu_2Ag_3-is2

bond	ρ	V	E	$\nabla^2\rho$
Ag1-Cu6	0.034	-0.033	-0.009	0.064
Ag1-Cu8	0.034	-0.033	-0.009	0.064
Ag1-Ag7	0.034	-0.034	-0.005	0.093
Ag1-Ag9	0.034	-0.034	-0.005	0.093
Cu6-Fe5	0.046	-0.043	-0.016	0.042
Cu8-Fe3	0.046	-0.043	-0.016	0.042
Ag7-Fe5	0.041	-0.035	-0.012	0.045
Ag9-Fe3	0.041	-0.035	-0.012	0.045
Cu6-Fe4	0.051	-0.049	-0.017	0.061
Cu8-Fe2	0.051	-0.049	-0.017	0.061
Ag9-Fe4	0.049	-0.044	-0.014	0.063
Ag7-Fe2	0.049	-0.044	-0.014	0.063
Ag1-Fe3	0.042	-0.037	-0.012	0.054
Ag1-Fe5	0.042	-0.037	-0.012	0.054
Cu6-Ag9	No (3,-1) b.c.p.			
Ag7-Cu8	No (3,-1) b.c.p.			

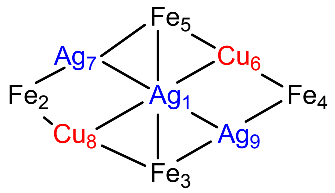

Cu_2Ag_3-is3

bond	ρ	V	E	$\nabla^2\rho$
Ag1-Cu6	0.036	-0.037	-0.009	0.072
Ag1-Cu9	0.036	-0.036	-0.009	0.072
Ag1-Ag7	0.032	-0.031	-0.005	0.085
Ag1-Ag8	0.032	-0.031	-0.005	0.085
Cu6-Fe5	0.046	-0.043	-0.016	0.043
Cu9-Fe3	0.046	-0.043	-0.016	0.043
Ag7-Fe5	0.042	-0.035	-0.012	0.045
Ag8-Fe3	0.042	-0.035	-0.012	0.045
Cu6-Fe4	0.052	-0.051	-0.017	0.063
Cu9-Fe4	0.052	-0.051	-0.017	0.063
Ag7-Fe2	0.048	-0.043	-0.014	0.062
Ag8-Fe2	0.048	-0.043	-0.014	0.062
Ag1-Fe3	0.042	-0.037	-0.012	0.054
Ag1-Fe5	0.042	-0.037	-0.012	0.054
Cu6-Cu9	No (3,-1) b.c.p.			
Ag7-Ag8	No (3,-1) b.c.p.			

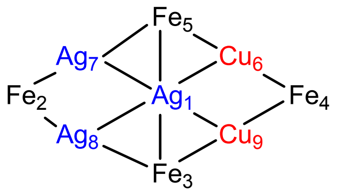

Cu_2Ag_3-is4

bond	ρ	V	E	$\nabla^2\rho$
Ag1-Cu6	0,034	-0,033	-0,009	0,064
Ag1-Cu7	0,034	-0,033	-0,009	0,064
Ag1-Ag9	0,034	-0,035	-0,005	0,094
Ag1-Ag8	0,034	-0,035	-0,006	0,094
Cu6-Fe5	0,047	-0,043	-0,016	0,042
Cu7-Fe5	0,047	-0,043	-0,016	0,042
Ag8-Fe3	0,041	-0,034	-0,012	0,044
Ag9-Fe3	0,041	-0,034	-0,012	0,044
Cu6-Fe4	0,050	-0,049	-0,017	0,060
Cu7-Fe2	0,050	-0,049	-0,017	0,060
Ag9-Fe4	0,049	-0,044	-0,014	0,063
Ag8-Fe2	0,049	-0,044	-0,014	0,063
Ag1-Fe3	0,040	-0,035	-0,011	0,050
Ag1-Fe5	0,044	-0,040	-0,013	0,059
Cu6-Ag9	No (3,-1) b.c.p.			
Cu7-Ag8	No (3,-1) b.c.p.			

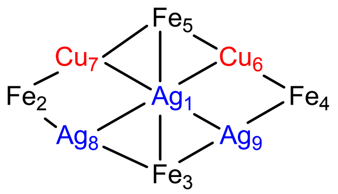

Table S17

Selected properties of (3,-1) b.c.p. in the isomers of $[CuAg_4Fe_4(CO)_{16}]^{3-}$. All quantities in a.u.

bond	ρ	*V*	*E*	$\nabla^2\rho$	
Cu1-Ag6	0.028	-0.025	-0.006	0.049	**CuAg$_4$-is1**
Cu1-Ag7	0.028	-0.025	-0.006	0.049	
Cu1-Ag9	0.028	-0.025	-0.006	0.049	
Cu1-Ag8	0.028	-0.025	-0.006	0.048	
Ag6-Fe5	0.044	-0.038	-0.013	0.053	
Ag8-Fe3	0.044	-0.039	-0.013	0.054	
Ag7-Fe5	0.044	-0.039	-0.013	0.053	
Ag9-Fe3	0.044	-0.039	-0.013	0.054	
Ag7-Fe2	0.048	-0.044	-0.014	0.063	
Ag8-Fe2	0.048	-0.044	-0.014	0.063	
Ag9-Fe4	0.048	-0.043	-0.014	0.063	
Ag6-Fe4	0.048	-0.044	-0.014	0.062	
Cu1-Fe3	0.042	-0.038	-0.015	0.036	
Cu1-Fe5	0.042	-0.038	-0.015	0.036	
Ag6-Ag9	0.032	-0.029	-0.005	0.078	
Ag7-Ag8	0.032	-0.029	-0.005	0.078	

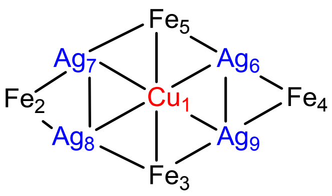

bond	ρ	*V*	*E*	$\nabla^2\rho$	
Ag1-Cu6	0.033	-0.033	-0.009	0.063	**CuAg$_4$-is2**
Ag1-Ag9	0.034	-0.034	-0.005	0.093	
Ag1-Ag7	0.031	-0.030	-0.005	0.083	
Ag1-Ag8	0.032	-0.030	-0.005	0.085	
Cu6-Fe5	0.046	-0.043	-0.016	0.044	
Ag7-Fe5	0.042	-0.035	-0.012	0.046	
Ag9-Fe3	0.041	-0.034	-0.012	0.046	
Ag8-Fe3	0.042	-0.035	-0.012	0.045	
Cu6-Fe4	0.051	-0.049	-0.017	0.061	
Ag9-Fe4	0.049	-0.045	-0.014	0.063	
Ag7-Fe2	0.048	-0.043	-0.014	0.062	
Ag8-Fe2	0.048	-0.043	-0.014	0.062	
Ag1-Fe3	0.040	-0.034	-0.011	0.050	
Ag1-Fe5	0.042	-0.037	-0.012	0.054	
Cu6-Ag9	No (3,-1) b.c.p.				
Ag7-Ag8	No (3,-1) b.c.p.				

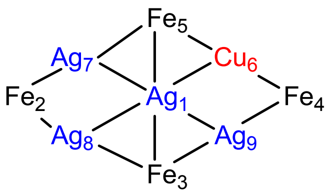

Table S18

Selected properties of (3,-1) b.c.p. in the isomers of $[Cu_4AuFe_4(CO)_{16}]^{3-}$. All quantities in a.u.

bond	ρ	*V*	*E*	$\nabla^2\rho$	
Au1-Cu6	0.040	-0.042	-0.009	0.097	**Cu_4Au-is1** 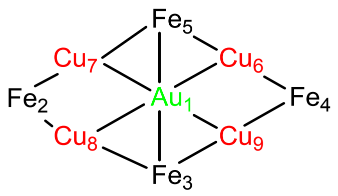
Au1-Cu7	0.041	0.042	-0.009	0.097	
Au1-Cu9	0.040	-0.042	-0.009	0.097	
Au1-Cu8	0.040	-0.042	0.009	0.097	
Cu6-Fe5	0.045	-0.042	-0.016	0.040	
Cu8-Fe3	0.045	-0.042	-0.016	0.040	
Cu7-Fe5	0.045	-0.041	-0.016	0.040	
Cu9-Fe3	0.045	-0.041	-0.016	0.040	
Cu7-Fe2	0.052	-0.050	-0.017	0.062	
Cu8-Fe2	0.052	-0.050	-0.017	0.061	
Cu9-Fe4	0.052	-0.050	-0.017	0.062	
Cu6-Fe4	0.051	-0.050	-0.017	0.061	
Au1-Fe3	0.051	-0.047	-0.014	0.074	
Au1-Fe5	0.051	-0.047	-0.014	0.074	
Cu6-Cu9	No (3,-1) b.c.p.				
Cu7-Cu8	No (3,-1) b.c.p.				
Cu1-Au6	0.040	-0.041	-0.009	0.096	**Cu_4Au-is2** 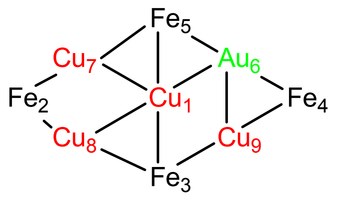
Cu1-Cu9	No (3,-1) b.c.p.				
Cu1-Cu7	0.035	-0,036	-0.013	0.042	
Cu1-Cu8	0.035	-0.035	-0.012	0.040	
Au6-Fe5	0.047	-0.042	-0.014	0.059	
Cu7-Fe5	0.048	-0.046	-0.017	0.047	
Cu9-Fe3	0.049	-0.047	-0.017	0.052	
Cu8-Fe3	0.049	-0.046	-0.017	0.050	
Au6-Fe4	0.056	-0.050	-0.015	0.075	
Cu9-Fe4	0.050	-0.049	-0.017	0.060	
Cu7-Fe2	0.052	-0.051	-0.018	0.063	
Cu8-Fe2	0.052	-0.051	-0.018	0.063	
Cu1-Fe3	0.046	-0.044	-0.016	0.047	
Cu1-Fe5	0.044	-0.041	-0.015	0.042	
Au6-Cu9	0.036	-0.035	-0.008	0.074	
Cu7-Cu8	No (3,-1) b.c.p.				

Table S19

Selected properties of (3,-1) b.c.p. in the isomers of $[Cu_3Au_2Fe_4(CO)_{16}]^{3-}$. All quantities in a.u.

bond	ρ	V	E	$\nabla^2\rho$
Au1-Au6	0.043	-0.044	-0.007	0,118
Au1-Cu9	0.039	-0.040	0.009	0,091
Au1-Cu7	0.041	-0.043	-0.009	0,101
Au1-Cu8	0.042	-0.044	-0.009	0,103
Cu7-Fe5	0.044	-0.041	-0.016	0,038
Cu8-Fe3	0.044	-0.041	-0.016	0,039
Cu9-Fe3	0.045	-0.042	-0.016	0,040
Au6-Fe5	0.047	-0.039	-0.012	0,055
Cu9-Fe4	0.050	-0.049	-0.017	0,059
Cu8-Fe2	0.051	-0.050	-0.017	0,061
Cu7-Fe2	0.052	-0.050	-0.017	0,062
Au6-Fe4	0.056	-0.051	-0.016	0,078
Au1-Fe3	0.051	-0.047	-0.014	0,075
Au1-Fe5	0.049	-0.044	-0.014	0,068
Au6-Cu9	No (3,-1) b.c.p.			
Cu7-Cu8	No (3,-1) b.c.p.			
Cu1-Au6	0.039	-0.040	-0.009	0.092
Cu1-Au8	0.039	-0.040	-0.009	0.092
Cu1-Cu7	No (3,-1) b.c.p.			
Cu1-Cu9	No (3,-1) b.c.p.			
Cu7-Fe5	0.049	-0.046	-0.017	0.050
Cu9-Fe3	0.049	-0.046	-0.017	0.050
Au6-Fe5	0.050	-0.043	-0.014	0.062
Au8-Fe3	0.050	-0.043	-0.014	0.062
Cu9-Fe4	0.050	-0.049	-0.017	0.059
Cu7-Fe2	0.050	-0.049	-0.017	0.059
Au6-Fe4	0.055	-0.050	-0.015	0.077
Au8-Fe2	0.055	-0.050	-0.015	0.077
Cu1-Fe3	0.043	-0.040	-0.015	0.040
Cu1-Fe5	0.043	-0.040	-0.015	0.040
Au6-Cu9	0.037	-0.036	-0.008	0.076
Cu7-Au8	0.039	-0.040	-0.009	0.092
Cu1-Au6	0,035	-0,035	-0,008	0,078
Cu1-Au9	0,035	-0,035	-0,076	0,078
Cu1-Cu7	0,034	-0,034	-0,012	0,038
Cu1-Cu8	0,034	-0,034	-0,012	0,038
Au6-Fe5	0,052	-0,045	-0,014	0,067
Au9-Fe3	0,052	-0,045	-0,014	0,067
Cu7-Fe5	0,049	-0,047	-0,017	0,050
Cu8-Fe3	0,049	-0,046	-0,017	0,050
Au6-Fe4	0,055	-0,050	-0,015	0,075
Au9-Fe4	0,055	-0,050	-0,015	0,075
Cu7-Fe2	0,052	-0,051	-0,018	0,063
Cu8-Fe2	0,052	-0,051	-0,018	0,063
Cu1-Fe3	0,043	-0,040	-0,015	0,040
Cu1-Fe5	0,043	-0,040	-0,015	0,040
Au6-Au9	0,035	-0,032	-0,004	0,093
Cu7-Cu8	No (3,-1) b.c.p.			
Cu1-Au6	0.040	-0.041	-0.008	0.097
Cu1-Au7	0.040	-0.041	-0.008	0.096
Cu1-Cu9	No (3,-1) b.c.p.			
Cu1-Cu8	No (3,-1) b.c.p.			
Au6-Fe5	0.050	-0.042	-0.014	0.058
Au7-Fe5	0.050	-0.042	-0.014	0.058
Cu9-Fe3	0.050	-0.048	-0.017	0.055
Cu8-Fe3	0.050	-0.048	-0.017	0.055
Au6-Fe4	0.056	-0.050	-0.015	0.076
Au7-Fe2	0.056	-0.050	-0.015	0.076
Cu9-Fe4	0.050	-0.049	-0.017	0.060
Cu8-Fe2	0.050	-0.049	-0.017	0.060
Cu1-Fe3	0.045	-0.043	-0.016	0.047
Cu1-Fe5	0.040	-0.037	-0.014	0.035
Au6-Cu9	0.037	-0.036	-0.008	0.076
Au7-Cu8	0.037	-0.036	-0.008	0.076

Cu_3Au_2-is1

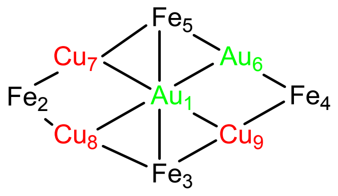

Cu_3Au_2-is2

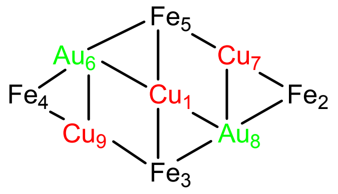

Cu_3Au_2-is3

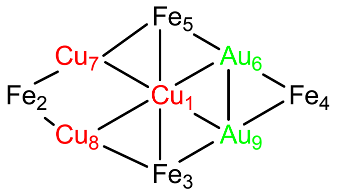

Cu_3Au_2-is4

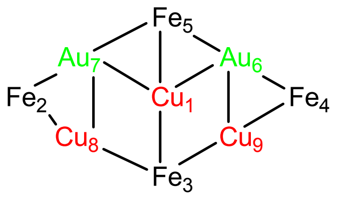

Table S20

Selected properties of (3,-1) b.c.p. in the isomers of $[Cu_2Au_3Fe_4(CO)_{16}]^{3-}$. All quantities in a.u.

Cu_2Au_3-is1

bond	ρ	V	E	$\nabla^2\rho$
Cu1-Cu6	No (3,-1) b.c.p.			
Cu1-Au9	0.038	-0.039	-0.008	0.089
Cu1-Au8	0.036	-0.036	-0.008	0.081
Cu1-Au7	0.035	-0.034	-0.008	0.075
Cu6-Fe5	0.050	-0.048	-0.017	0.055
Au7-Fe5	0.052	-0.046	-0.014	0.069
Au9-Fe3	0.050	-0.043	-0.014	0.061
Au8-Fe3	0.051	-0.045	-0.014	0.066
Cu6-Fe4	0.049	-0.049	-0.017	0.061
Au9-Fe4	0.055	-0.049	-0.015	0.074
Au8-Fe2	0.055	-0.049	-0.015	0.075
Au7-Fe2	0.055	-0.049	-0.015	0.074
Cu1-Fe3	0.039	-0.035	-0.013	0.033
Cu1-Fe5	0.042	-0.039	-0.015	0.039
Au7-Au8	0.036	-0.033	-0.005	0.096
Cu6-Au9	0.039	-0.039	-0.009	0.086

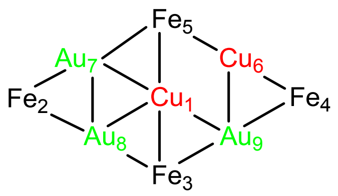

Cu_2Au_3-is2

bond	ρ	V	E	$\nabla^2\rho$
Au1-Cu6	0.040	-0.041	-0.009	0.094
Au1-Cu8	0.040	-0.041	-0.009	0.094
Au1-Au7	0.044	-0.046	-0.008	0.119
Au1-Au9	0.044	-0.046	-0.008	0.119
Cu6-Fe5	0.045	-0.041	-0.016	0.038
Cu8-Fe3	0.045	-0.041	-0.016	0.038
Au7-Fe5	0.046	-0.038	-0.012	0.055
Au9-Fe3	0.046	-0.038	-0.012	0.055
Cu6-Fe4	0.050	-0.049	-0.017	0.060
Cu8-Fe2	0.050	-0.049	-0.017	0.060
Au9-Fe4	0.056	-0.051	-0.016	0.078
Au7-Fe2	0.056	-0.051	-0.016	0.078
Au1-Fe3	0.049	-0.044	-0.013	0.068
Au1-Fe5	0.049	-0.044	-0.013	0.068
Cu6-Au9	No (3,-1) b.c.p.			
Au7-Cu8	No (3,-1) b.c.p.			

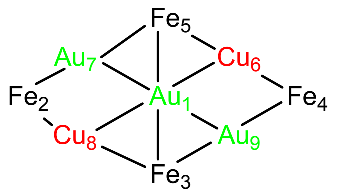

Cu_2Au_3-is3

bond	ρ	V	E	$\nabla^2\rho$
Au1-Cu6	0.042	-0.044	-0.009	0.103
Au1-Cu9	0.042	-0.044	-0.009	0.102
Au1-Au7	0.040	-0.040	-0.006	0.111
Au1-Au8	0.040	-0.040	-0.006	0.111
Cu6-Fe5	0.045	-0.041	-0.016	0.040
Cu9-Fe3	0.045	-0.041	-0.016	0.040
Au7-Fe5	0.048	-0.041	-0.013	0.059
Au8-Fe3	0.048	-0.041	-0.013	0.059
Cu6-Fe4	0.051	-0.050	-0.017	0.061
Cu9-Fe4	0.051	-0.050	-0.017	0.062
Au7-Fe2	0.055	-0.050	-0.015	0.076
Au8-Fe2	0.055	-0.050	-0.015	0.075
Au1-Fe3	0.049	-0.043	-0.013	0.067
Au1-Fe5	0.049	-0.043	-0.013	0.066
Cu6-Cu9	No (3,-1) b.c.p.			
Au7-Au8	No (3,-1) b.c.p.			

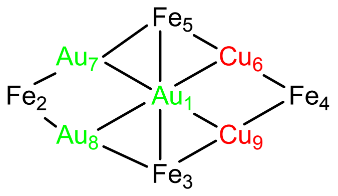

Cu_2Au_3-is4

bond	ρ	V	E	$\nabla^2\rho$
Au1-Cu6	0.040	-0.041	-0.009	0.095
Au1-Cu7	0.040	-0.041	-0.009	0.094
Au1-Au9	0.044	-0.046	-0.008	0.122
Au1-Au8	0.044	-0.046	-0.008	0.122
Cu6-Fe5	0.045	-0.041	-0.016	0.040
Cu7-Fe5	0.045	-0.041	-0.016	0.040
Au8-Fe3	0.046	-0.038	-0.012	0.054
Au9-Fe3	0.046	-0.038	-0.012	0.054
Cu6-Fe4	0.050	-0.048	-0.017	0.059
Cu7-Fe2	0.050	-0.048	-0.017	0.059
Au9-Fe4	0.056	-0.051	-0.016	0.079
Au8-Fe2	0.056	-0.051	-0.016	0.079
Au1-Fe3	0.046	-0.040	-0.013	0.060
Au1-Fe5	0.051	-0.047	-0.014	0.076
Cu6-Au9	No (3,-1) b.c.p.			
Cu7-Au8	No (3,-1) b.c.p.			

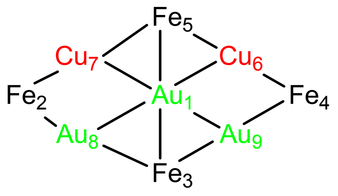

Table S21

Selected properties of (3,-1) b.c.p. in the isomers of $[CuAu_4Fe_4(CO)_{16}]^{3-}$. All quantities in a.u.

bond	ρ	*V*	*E*	$\nabla^2\rho$	
Cu1-Au6	0.034	-0.034	-0.007	0.076	**CuAu$_4$-is1**
Cu1-Au8	0.034	-0.033	-0.007	0.074	
Cu1-Au9	0.034	-0.033	-0.007	0.074	
Cu1-Au7	0.034	-0.033	-0.007	0.074	
Au6-Fe5	0.052	-0.046	-0.014	0.068	
Au7-Fe5	0.052	-0.046	-0.014	0.068	
Au8-Fe3	0.052	-0.046	-0.014	0.068	
Au9-Fe3	0.052	-0.046	-0.014	0.068	
Au6-Fe4	0.055	-0.049	-0.015	0.075	
Au9-Fe4	0.055	-0.049	-0.015	0.075	
Au7-Fe2	0.055	-0.049	-0.015	0.075	
Au8-Fe2	0.055	-0.049	-0.015	0.075	
Au6-Fe4	0.055	-0.049	-0.015	0.075	
Au9-Fe4	0.055	-0.049	-0.015	0.075	
Cu1-Fe3	0.036	-0.032	-0.012	0.030	
Cu1-Fe5	0.036	-0.032	-0.012	0.030	
Au1-Cu6	0.040	-0.041	-0.009	0.095	**CuAu$_4$-is2**
Au1-Au8	0.042	-0.042	-0.007	0.114	
Au1-Au9	0.044	-0.045	-0.008	0.119	
Au1-Au7	0.041	-0.041	-0.007	0.111	
Cu6-Fe5	0.045	-0.042	-0.016	0.041	
Au7-Fe5	0.048	-0.041	-0.013	0.060	
Au9-Fe3	0.046	-0.038	-0.012	0.055	
Au8-Fe3	0.047	-0.040	-0.012	0.059	
Cu6-Fe4	0.049	-0.048	-0.017	0.059	
Au9-Fe4	0.056	-0.050	-0.015	0.077	
Au7-Fe2	0.055	-0.050	-0.016	0.078	
Au8-Fe2	0.055	-0.050	-0.015	0.076	
Au1-Fe3	0.046	-0.040	-0.012	0.059	
Au1-Fe5	0.049	0.043	-0.013	0.067	
Ag6-Au9	No (3,-1) b.c.p.				
Au7-Au8	No (3,-1) b.c.p.				

CuAu$_4$-is1

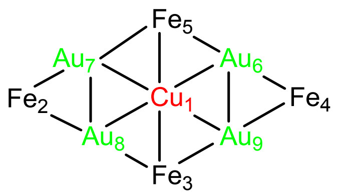

CuAu$_4$-is2

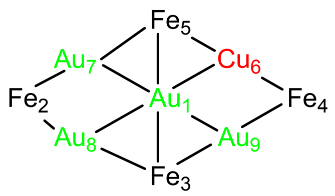

Table S22

Selected properties of (3,-1) b.c.p. in the isomers of [$Ag_4AuFe_4(CO)_{16}$]$^{3-}$. All quantities in a.u.

bond	ρ	*V*	*E*	$\nabla^2\rho$	
Au1-Ag6	0.035	-0.034	-0.005	0.096	**Ag$_4$Au-is1** 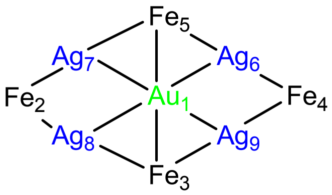
Au1-Ag7	0.035	-0.034	-0.005	0.096	
Au1-Ag9	0.035	-0.034	-0.005	0.096	
Au1-Ag8	0.035	-0.034	-0.005	0.096	
Ag6-Fe5	0.041	-0.034	-0.011	0.048	
Ag8-Fe3	0.041	-0.034	-0.011	0.048	
Ag7-Fe5	0.041	-0.034	-0.011	0.048	
Ag9-Fe3	0.041	-0.034	-0.011	0.048	
Ag7-Fe2	0.047	-0.043	-0.014	0.062	
Ag8-Fe2	0.047	-0.043	-0.014	0.062	
Ag9-Fe4	0.047	-0.043	-0.014	0.062	
Ag6-Fe4	0.047	-0.043	-0.014	0.062	
Au1-Fe3	0.047	-0.041	-0.013	0.062	
Au1-Fe5	0.047	-0.041	-0.013	0.061	
Ag6-Ag9	No (3,-1) b.c.p.				
Ag7-Ag8	No (3,-1) b.c.p.				
Ag1-Au6	0.037	-0.037	-0.058	0,103	**Ag$_4$Au-is2** 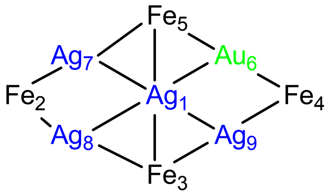
Ag1-Ag9	0.031	-0.030	-0.004	0,085	
Ag1-Ag7	0.031	-0.030	-0.046	0,084	
Ag1-Ag8	0.032	-0.030	-0.005	0,084	
Au6-Fe5	0.048	-0.040	-0.013	0.060	
Ag7-Fe5	0.042	-0.035	-0.012	0.045	
Ag9-Fe3	0.042	-0.035	-0.012	0.047	
Ag8-Fe3	0.042	-0.035	-0.012	0.046	
Au6-Fe4	0.055	-0.050	-0.015	0.077	
Ag9-Fe4	0.047	-0.043	-0.014	0.061	
Ag7-Fe2	0.048	-0.043	-0.014	0.062	
Ag8-Fe2	0.047	-0.043	-0.014	0.061	
Ag1-Fe3	0.039	-0.034	-0.011	0.049	
Ag1-Fe5	0.039	-0.034	-0.011	0.049	
Au6-Ag9	No (3,-1) b.c.p.				
Ag7-Ag8	No (3,-1) b.c.p.				

Table S23

Selected properties of (3,-1) b.c.p. in the isomers of $[Ag_3Au_2Fe_4(CO)_{16}]^{3-}$. All quantities in a.u.

bond	ρ	*V*	*E*	$\nabla^2\rho$
Au1-Au6	0.039	-0.038	-0.006	0.105
Au1-Ag9	0.036	-0.035	-0.005	0.099
Au1-Ag7	0.036	-0.035	-0.005	0.098
Au1-Ag8	0.036	-0.035	-0.005	0.099
Ag7-Fe5	0.040	-0.034	-0.011	0.046
Ag8-Fe3	0.040	-0.034	-0.011	0.047
Ag9-Fe3	0.041	-0.035	-0.011	0.048
Au6-Fe5	0.047	-0.040	-0.012	0.065
Ag9-Fe4	0.047	-0.043	0.014	0,061
Ag8-Fe2	0.047	-0.043	-0.014	0.061
Ag7-Fe2	0.047	-0.043	-0.014	0.062
Au6-Fe4	0.056	-0.050	-0.015	0.078
Au1-Fe3	0.047	-0.041	-0.013	0.062
Au1-Fe5	0.047	-0.040	-0.013	0.061
Au6-Ag9	No (3,-1) b.c.p.			
Ag7-Ag8	No (3,-1) b.c.p.			

Ag_3Au_2-is1

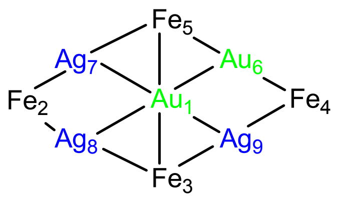

bond	ρ	*V*	*E*	$\nabla^2\rho$
Ag1-Au6	0.038	-0.038	-0.006	0.104
Ag1-Au8	0.038	-0.038	-0.006	0.104
Ag1-Ag7	0.031	-0.030	-0.004	0.085
Ag1-Ag9	0.031	-0.030	-0.004	0.085
Ag7-Fe5	0.042	-0.035	-0.012	0.046
Ag9-Fe3	0.042	-0.035	-0.012	0.046
Au6-Fe5	0.048	-0.040	-0.013	0,086
Au8-Fe3	0.048	-0.040	-0.013	0.059
Ag9-Fe4	0.047	-0.043	-0.014	0,061
Ag7-Fe2	0.047	-0.043	-0.014	0.061
Au6-Fe4	0.055	-0.050	-0.015	0.077
Au8-Fe2	0.055	-0.050	-0.015	0.077
Ag1-Fe3	0.038	-0.033	-0.010	0.048
Ag1-Fe5	0.038	-0.033	-0.010	0.048
Ag7-Au8	No (3,-1) b.c.p.			
Au6-Ag9	No (3,-1) b.c.p.			

Ag_3Au_2-is2

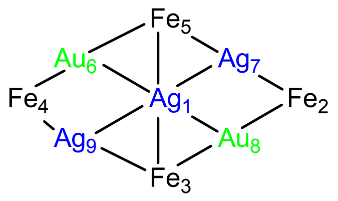

bond	ρ	*V*	*E*	$\nabla^2\rho$
Ag1-Au6	0.037	-0,037	-0,006	0,120
Ag1-Au9	0.037	-0,037	-0,056	0,103
Ag1-Ag7	0.032	-0,030	-0,047	0,084
Ag1-Ag8	0.032	-0,030	-0,047	0,084
Au6-Fe5	0.049	-0.041	-0.013	0.064
Au9-Fe3	0.049	-0.041	-0.013	0.064
Ag7-Fe5	0.042	-0.035	-0.012	0.045
Ag8-Fe3	0.042	-0.035	-0.012	0.045
Au6-Fe4	0.056	-0.050	-0.016	0.076
Au9-Fe4	0.056	-0.050	-0.016	0.076
Ag7-Fe2	0.047	-0.043	-0.014	0.062
Ag8-Fe2	0.047	-0.043	-0.014	0.062
Ag1-Fe3	0.039	-0.033	-0.010	0.048
Ag1-Fe5	0.039	-0.033	-0.010	0.048
Au6-Au9	No (3,-1) b.c.p.			
Ag7-Ag8	No (3,-1) b.c.p.			

Ag_3Au_2-is3

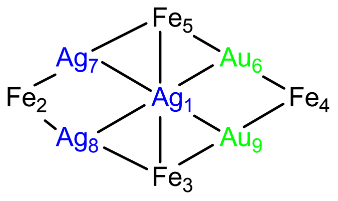

bond	ρ	*V*	*E*	$\nabla^2\rho$
Ag1-Au6	0.038	-0,039	-0,061	0,106
Ag1-Au7	0.038	-0,039	-0,061	0,106
Ag1-Ag9	0.031	-0,030	-0,005	0,085
Ag1-Ag8	0.031	-0,030	-0,005	0,085
Au6-Fe5	0.048	-0.040	-0.013	0.056
Au7-Fe5	0.048	-0.040	-0.013	0.057
Ag9-Fe3	0.042	-0.035	-0.012	0.047
Ag8-Fe3	0.042	-0.035	-0.012	0.047
Au6-Fe4	0.055	-0.050	-0.015	0.077
Au7-Fe2	0.055	-0.050	-0.015	0.077
Ag9-Fe4	0.047	-0.043	-0.014	0.061
Ag8-Fe2	0.047	-0.043	-0.014	0.061
Ag1-Fe3	0.039	-0.033	-0.010	0.048
Ag1-Fe5	0.037	-0.032	-0.010	0.047
Au6-Ag9	No (3,-1) b.c.p.			
Au7-Ag8	No (3,-1) b.c.p.			

Ag_3Au_2-is4

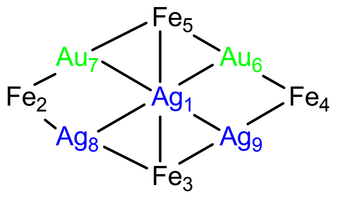

Table S24

Selected properties of (3,-1) b.c.p. in the isomers of $[Ag_2Au_3Fe_4(CO)_{16}]^{3-}$. All quantities in a.u.

bond	ρ	*V*	*E*	$\nabla^2\rho$	
Ag1-Ag6	0.032	-0.031	-0.005	0.086	**Ag_2Au_3-is1** 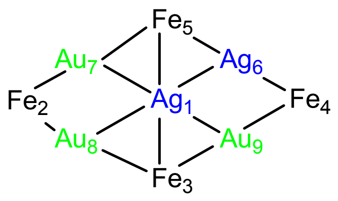
Ag1-Au9	0.038	-0.039	-0.006	0.106	
Ag1-Au8	0.037	-0.038	-0.006	0.106	
Ag1-Au7	0.037	-0.038	-0.006	0.104	
Ag6-Fe5	0.042	-0.036	-0.012	0.046	
Au7-Fe5	0.049	-0.041	-0.013	0.062	
Au9-Fe3	0.048	-0.040	-0.013	0.057	
Au8-Fe3	0.049	-0.041	-0.013	0.059	
Ag6-Fe4	0.047	-0.043	-0.014	0.061	
Au9-Fe4	0.055	-0.050	-0.015	0.076	
Au8-Fe2	0.055	-0.050	-0.016	0.076	
Au7-Fe2	0.055	-0.050	-0.016	0.076	
Ag1-Fe3	0.037	-0.031	-0.010	0.047	
Ag1-Fe5	0.038	-0.032	-0.010	0.047	
Ag6-Au9	No (3,-1) b.c.p.				
Au7-Au8	No (3,-1) b.c.p.				
Au1-Ag6	0.036	-0.035	-0.005	0.099	**Ag_2Au_3-is2** 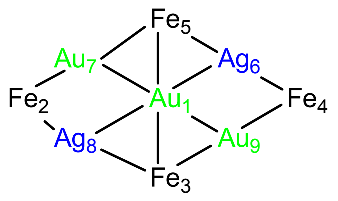
Au1-Ag8	0.036	-0.035	-0.005	0.099	
Au1-Au7	0.041	-0.040	-0.006	0.188	
Au1-Au9	0.040	-0.040	-0.006	0.108	
Ag6-Fe5	0.040	-0.034	-0.011	0.046	
Ag8-Fe3	0.040	-0.034	-0.011	0.046	
Au7-Fe5	0.047	-0.040	-0.012	0.063	
Au9-Fe3	0.048	-0.040	-0.012	0.063	
Ag6-Fe4	0.047	-0.043	-0.014	0.060	
Ag8-Fe2	0.047	-0.043	-0.014	0.060	
Au9-Fe4	0.055	-0.050	-0.015	0.077	
Au7-Fe2	0.055	-0.050	-0.015	0.077	
Au1-Fe3	0.046	-0.040	-0.013	0.061	
Au1-Fe5	0.046	-0.040	-0.013	0.061	
Ag6-Au9	No (3,-1) b.c.p.				
Au7-Ag8	No (3,-1) b.c.p.				
Au1-Ag6	0.036	-0.036	-0.006	0,099	**Ag_2Au_3-is3** 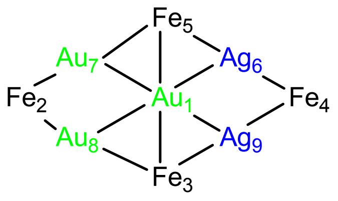
Au1-Ag9	0.036	-0.036	-0.006	0.099	
Au1-Au7	0.039	-0.039	-0.006	0.107	
Au1-Au8	0.039	-0.039	-0.006	0.107	
Ag6-Fe5	0.040	-0.034	-0.011	0.045	
Ag9-Fe3	0.040	-0.034	-0.011	0.045	
Au7-Fe5	0.048	-0.041	-0.012	0.069	
Au8-Fe3	0.048	-0.041	-0.012	0.069	
Ag6-Fe4	0.047	-0.043	-0.014	0.061	
Ag9-Fe4	0.047	-0.043	-0.014	0.061	
Au7-Fe2	0.056	-0.050	-0.016	0.076	
Au8-Fe2	0.056	-0.050	-0.016	0.076	
Au1-Fe3	0.047	-0.040	-0.013	0.061	
Au1-Fe5	0.046	-0.040	-0.013	0.061	
Ag6-Ag9	No (3,-1) b.c.p.				
Au7-Au8	No (3,-1) b.c.p.				
Au1-Ag6	0.036	-0.035	-0.005	0,099	**Ag_2Au_3-is4** 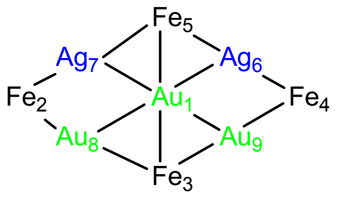
Au1-Ag7	0.036	-0.035	-0.005	0,099	
Au1-Au9	0.041	-0.040	-0.007	0,110	
Au1-Au8	0.041	-0.040	-0.006	0,110	
Ag6-Fe5	0.041	-0.034	-0.011	0.047	
Ag7-Fe5	0.041	-0.034	-0.011	0.047	
Au8-Fe3	0.047	-0.040	-0.013	0.059	
Au9-Fe3	0.047	-0.040	-0.013	0.059	
Ag6-Fe4	0.047	-0.043	-0.014	0.060	
Ag7-Fe2	0.047	-0.042	-0.014	0.060	
Au9-Fe4	0.055	-0.050	-0.015	0.078	
Au8-Fe2	0.055	-0.050	-0.015	0.078	
Au1-Fe3	0.046	-0.040	-0.012	0.060	
Au1-Fe5	0.047	-0.041	-0.013	0.062	
Ag6-Au9	No (3,-1) b.c.p.				
Ag7-Au8	No (3,-1) b.c.p.				

Table S25

Selected properties of (3,-1) b.c.p. in the isomers of $[AgAu_4Fe_4(CO)_{16}]^{3-}$. All quantities in a.u.

bond	ρ	*V*	*E*	$\nabla^2\rho$	
Ag1-Au6	0.038	-0.039	-0.006	0.107	**$AgAu_4$-is1** 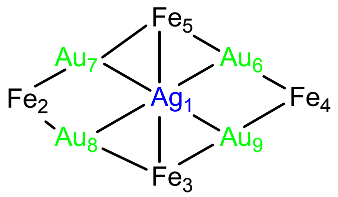
Ag1-Au8	0.038	-0.039	-0.006	0.106	
Ag1-Au9	0.038	-0.039	-0.006	0.106	
Ag1-Au7	0.037	-0.038	-0.006	0.104	
Au6-Fe5	0.049	-0.041	-0.013	0.059	
Au7-Fe5	0.049	-0.041	-0.013	0.059	
Au8-Fe3	0.049	-0.042	-0.013	0.059	
Au9-Fe3	0.049	-0.042	-0.013	0.059	
Au6-Fe4	0.055	-0.050	-0.015	0.075	
Au9-Fe4	0.055	-0.050	-0.015	0.075	
Au7-Fe2	0.055	-0.049	-0.015	0.075	
Au8-Fe2	0.055	-0.050	-0.015	0.076	
Ag1-Fe3	0.036	-0.030	-0.009	0.045	
Ag1-Fe5	0.036	-0.030	-0.009	0.050	
Au6-Au9	No (3,-1) b.c.p.				
Au7-Au8	No (3,-1) b.c.p.				
Au1-Ag6	0.037	-0.037	-0.006	0.101	**$AgAu_4$-is2** 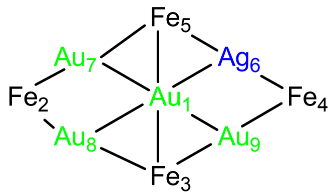
Au1-Au8	0.041	-0.041	-0.007	0.111	
Au1-Au9	0.041	-0.041	-0.007	0.111	
Au1-Au7	0.040	-0.040	-0.006	0.109	
Ag6-Fe5	0.041	-0.034	-0.011	0.046	
Au7-Fe5	0.048	-0.040	-0.011	0.074	
Au9-Fe3	0.047	-0.039	-0.012	0.058	
Au8-Fe3	0.048	-0.041	-0.013	0.062	
Ag6-Fe4	0.046	-0.042	-0.014	0.060	
Au9-Fe4	0.055	-0.050	-0.015	0.077	
Au7-Fe2	0.055	-0.050	-0.016	0.076	
Au8-Fe2	0.055	-0.050	-0.015	0.076	
Au1-Fe3	0.045	-0.039	-0.012	0.059	
Au1-Fe5	0.046	-0.040	-0.012	0.061	
Ag6-Au9	No (3,-1) b.c.p.				
Au7-Au8	No (3,-1) b.c.p.				

Table S26

Crystal data and experimental details for $[NEt_4]_3[Ag_xCu_{5-x}Fe_4(CO)_{16}]$

	x = 1.02	x = 3.30	x = 3.45
Formula	$C_{40}H_{60}Ag_{1.02}Cu_{3.98}Fe_4N_3O_{16}$	$C_{40}H_{60}Ag_{3.30}Cu_{1.70}Fe_4N_3O_{16}$	$C_{40}H_{60}Ag_{3.45}Cu_{1.55}Fe_4N_3O_{16}$
Fw	1425.44	1526.30	1532.72
T, K	100(2)	100(2)	100(2)
λ, Å	0.71073	0.71073	0.71073
Crystal system	Tetragonal	Tetragonal	Tetragonal
Space Group	$P4_2/mnm$	$P4_2/mnm$	$P4_2/mnm$
a, Å	13.5295(12)	13.7039(4)	13.7170(3)
b, Å	13.5295(12)	13.7039(4)	13.7170(3)
c, Å	14.2521(13)	14.1151(4)	14.1238(3)
Cell Volume, Å^3	2608.8(5)	2650.77(17)	2657.48(13)
Z	2	2	2
D_c, g cm^{-3}	1.815	1.912	1.915
μ, mm^{-1}	3.105	2.984	2.972
F(000)	1433	1515	1520
Crystal size, mm	0.24 × 0.18 × 0.14	0.23 × 0.21 × 0.16	0.22 × 0.20 × 0.15
θ limits, °	2.075-26.998	2.071-25.999	2.100-26.993
Reflections collected	35198	34949	40231
R_{int}	0.0558	0.0397	0.0454
Data / restraints / parameters	1578 / 60 / 135	1445 / 48 / 136	1603 / 66 / 126
Goodness on fit on F^2	1.162	1.269	1.252
R_1 (I > 2σ(I))	0.0475	0.0385	0.0385
wR_2 (all data)	0.1047	0.0812	0.0850
Absolute structure parameter	-	-	-
Largest diff. peak and hole, e Å^{-3}	0.800 / -0.819	0.703 / -0.650	0.960 / -0.790

	x = 4.25	x = 4.37	x = 4.81	x = 4.88
Formula	$C_{40}H_{60}Ag_{4.25}Cu_{0.75}Fe_4N_3O_{16}$	$C_{40}H_{60}Ag_{4.37}Cu_{0.63}Fe_4N_3O_{16}$	$C_{40}H_{60}Ag_{4.81}Cu_{0.20}Fe_4N_3O_{16}$	$C_{40}H_{60}Ag_{4.88}Cu_{0.12}Fe_4N_3O_{16}$
Fw	1568.63	1573.73	1593.01	1596.34
T, K	100(2)	100(2)	100(2)	100(2)
λ, Å	0.71073	0.71073	0.71073	0.71073
Crystal system	Tetragonal	Tetragonal	Tetragonal	Tetragonal
Space Group	$P\bar{4}2_1m$	$P\bar{4}2_1m$	$P\bar{4}2_1m$	$P\bar{4}2_1m$
a, Å	13.7951(10)	13.7996(9)	13.8437(10)	13.8577(6)
b, Å	13.7951(10)	13.7996(9)	13.8437(10)	13.8577(6)
c, Å	14.0408(11)	14.0318(9)	13.9515(11)	13.9705(7)
Cell Volume, Å^3	2672.0(4)	2672.1(4)	2673.8(4)	2682.8(3)
Z	2	2	2	2
D_c, g cm^{-3}	1.950	1.956	1.979	1.976
μ, mm^{-1}	2.930	2.927	2.911	2.899
F(000)	1549	1553	1569	1572

Crystal size, mm	0.19 × 0.18 × 0.14	0.25 × 0.21 × 0.18	0.24 × 0.21 × 0.18	0.22 × 0.18 × 0.12
θ limits, °	1.450-28.000	2.087-27.996	1.460-25.992	1.458-26.997
Reflections collected	40226	44337	35173	37946
R_{int}	0.0345	0.0438	0.0376	0.0405
Data / restraints / parameters	3409 / 34 / 203	3403 / 82 / 213	2790 / 16 / 213	3107 / 16 / 219
Goodness on fit on F^2	1.100	1.201	1.187	1.164
R_1 ($I > 2\sigma(I)$)	0.0255	0.0338	0.0194	0.0201
wR_2 (all data)	0.0597	0.0763	0.0447	0.0433
Absolute structure parameter	0.48(5)	0.52(6)	0.49(4)	0.50(3)
Largest diff. peak and hole, e Å^{-3}	0.911 / -0.593	0.828 / -0.604	0.273 / -0.5724	0.290 / -0.644

	**x = 4.90**	**x = 4.92**	**x = 5**
Formula	$C_{40}H_{60}Ag_{4.90}Cu_{0.10}Fe_4N_3O_{16}$	$C_{40}H_{60}Ag_{4.92}Cu_{0.08}Fe_4N_3O_{16}$	$C_{40}H_{60}Ag_5Cu_0Fe_4N_3O_{16}$
Fw	1597.22	1598.11	1601.66
T, K	100(2)	100(2)	100(2)
λ, Å	0.71073	0.71073	0.71073
Crystal system	Tetragonal	Tetragonal	Tetragonal
Space Group	$P\bar{4}2_1m$	$P\bar{4}2_1m$	$P\bar{4}2_1m$
a, Å	13.8637(8)	13.8468(8)	13.8450(7)
b, Å	13.8637(8)	13.8468(8)	13.8450(7)
c, Å	13.9770(8)	13.9725(9)	13.9743(8)
Cell Volume, Å^3	2686.4(3)	2679.0(4)	2678.7(3)
Z	2	2	2
D_c, g cm^{-3}	1.975	1.981	1.986
μ, mm^{-1}	2.894	2.902	2.900
F(000)	1572	1573	1576
Crystal size, mm	0.22 × 0.19 × 0.16	0.21 × 0.16 × 0.14	0.25 × 0.16 × 0.14
θ limits, °	1.457-26.996	1.457-26.997	1.457-27.991
Reflections collected	38184	37735	40908
R_{int}	0.0390	0.0348	0.0378
Data / restraints / parameters	3111 / 16 / 215	3413 / 129 / 207	3413 / 129 / 207
Goodness on fit on F^2	1.130	1.115	1.105
R_1 ($I > 2\sigma(I)$)	0.0192	0.0179	0.0208
wR_2 (all data)	0.0422	0.0423	0.0446
Absolute structure parameter	0.47(3)	0.45(3)	0.50(3)
Largest diff. peak and hole, e Å^{-3}	0.268 / -0.682	0.369 / -0.604	0.334/ -0.522

Table S27

Crystal data and experimental details for $[NEt_4]_3[Au_xCu_{5-x}Fe_4(CO)_{16}]$

	**x = 1.09**	**x = 1.15**	**x = 1.31**
Formula	$C_{40}H_{60}Au_{1.09}Cu_{3.91}Fe_4N_3O_{16}$	$C_{40}H_{60}Au_{1.15}Cu_{3.85}Fe_4N_3O_{16}$	$C_{40}H_{60}Au_{1.31}Cu_{3.69}Fe_4N_3O_{16}$
Fw	1525.44	1534.11	1555.46
T, K	100(2)	100(2)	100(2)
λ, Å	0.71073	0.71073	0.71073
Crystal system	Tetragonal	Tetragonal	Tetragonal
Space Group	*P*4$_2$*/mnm*	*P*4$_2$*/mnm*	*P*4$_2$*/mnm*
a, Å	13.5323(6)	13.5442(6)	13.5598(7)
b, Å	13.5323(6)	13.5442(6)	13.5598(7)
c, Å	14.2471(7)	14.2462(7)	14.2358(7)
Cell Volume, Å^3	2609.0(3)	2613.4(3)	2617.5(3)
Z	2	2	2
D_c, g cm^{-3}	1.942	1.950	1.974
μ, mm^{-1}	5.755	5.901	6.275
F(000)	1505	1512	1528
Crystal size, mm	0.21 × 0.16 × 0.14	0.21 × 0.18 × 0.15	0.18 × 0.16 × 0.12
θ limits, °	2.076-27.992	2.075-27.992	2.074-27.989
Reflections collected	38104	37872	38067
R_{int}	0.0426	0.0414	0.0517
Data / restraints / parameters	1730 / 54 / 125	1732 / 72 / 135	1736 / 54 / 135
Goodness on fit on F^2	1.295	1.293	1.298
R_1 (I > 2σ(I))	0.0511	0.0407	0.0443
wR_2 (all data)	0.0969	0.0859	0.0917
Absolute structure parameter	-	-	-
Largest diff. peak and hole, e Å^{-3}	1.128 / -1.105	0.744 / -0.743	0.640 / -1.434

	**x = 1.67**	**x = 2.18**	**x = 2.48**
Formula	$C_{40}H_{60}Au_{1.67}Cu_{3.33}Fe_4N_3O_{16}$	$C_{40}H_{60}Au_{2.18}Cu_{2.83}Fe_4N_3O_{16}$	$C_{40}H_{60}Au_{2.48}Cu_{2.52}Fe_4N_3O_{16}$
Fw	1602.83	1670.21	1710.90
T, K	100(2)	100(2)	100(2)
λ, Å	0.71073	0.71073	0.71073
Crystal system	Tetragonal	Tetragonal	Tetragonal
Space Group	*P*4$_2$*/mnm*	*P*4$_2$*/mnm*	*P*4$_2$*/mnm*
a, Å	13.5914(9)	13.6169(14)	13.6673(10)
b, Å	13.5914(9)	13.6169(14)	13.6673(10)
c, Å	14.1855(10)	14.1414(14)	14.1095(11)
Cell Volume, Å^3	2620.4(4)	2622.1(6)	2635.6(4)
Z	2	2	2
D_c, g cm^{-3}	2.031	2.115	2.156
μ, mm^{-1}	7.116	8.318	9.001
F(000)	1563	1614	1644

Crystal size, mm	0.18 × 0.16 × 0.13	0.22 × 0.18 × 0.16	0.18 × 0.16 × 0.12
θ limits, °	2.075-27.993	2.076-25.989	2.075-25.996
Reflections collected	37818	26179	32404
R_{int}	0.0565	0.0683	0.0495
Data / restraints / parameters	1737 / 60 / 135	1432 / 102 / 135	1441 / 72 / 129
Goodness on fit on F^2	1.381	1.301	1.366
R_1 (I > 2σ(I))	0.0555	0.0774	0.0404
wR_2 (all data)	0.1053	0.1452	0.0891
Absolute structure parameter	-	-	-
Largest diff. peak and hole, e Å^{-3}	1.051 / -1.484	1.764 / -2.488	1.252 / -1.510

	**x = 2.73**	**x = 4.59**	**x = 4.61**
Formula	$C_{40}H_{60}Au_{2.73}Cu_{2.28}Fe_4N_3O_{16}$	$C_{40}H_{60}Au_{4.59}Cu_{0.42}Fe_4N_3O_{16}$	$C_{40}H_{60}Au_{4.61}Cu_{0.38}Fe_4N_3O_{16}$
Fw	1743.59	1991.77	1995.77
T, K	100(2)	100(2)	100(2)
λ, Å	0.71073	0.71073	0.71073
Crystal system	Tetragonal	Tetragonal	Tetragonal
Space Group	$P4_2/mnm$	$P\bar{4}2_1m$	$P\bar{4}2_1m$
a, Å	13.6687(6)	13.8018(9)	13.8200(9)
b, Å	13.6687(6)	13.8018(9)	13.8200(9)
c, Å	14.1028(7)	13.9293(9)	13.9448(9)
Cell Volume, Å^3	2634.9(3)	2653.4(4)	2663.4(4)
Z	2	2	2
D_c, g cm^{-3}	2.198	2.493	2.489
μ, mm^{-1}	9.586	13.910	13.928
F(000)	1668	1854	1858
Crystal size, mm	0.18 × 0.16 × 0.12	0.16 × 0.14 × 0.11	0.22 × 0.19 × 0.15
θ limits, °	2.075-25.998	1.462-25.998	1.460-27.985
Reflections collected	33368	33650	40502
R_{int}	0.0452	0.0666	0.0499
Data / restraints / parameters	1440 / 84 / 141	2766 / 148 / 209	3394 / 82 / 203
Goodness on fit on F^2	1.277	1.132	1.165
R_1 (I > 2σ(I))	0.0506	0.0349	0.0343
wR_2 (all data)	0.0965	0.0743	0.0665
Absolute structure parameter	-	0.49(2)	0.50(2)
Largest diff. peak and hole, e Å^{-3}	1.517 / -2.166	1.651 / -1.087	1.890 / -1.364

Table S28

Crystal data and experimental details for $[NEt_4]_3[Au_xAg_{5-x}Fe_4(CO)_{16}]$

	**x = 0.64**	**x = 0.81**
Formula	$C_{40}H_{60}Au_{0.64}Ag_{4.36}Fe_4N_3O_{16}$	$C_{40}H_{60}Au_{0.81}Ag_{4.20}Fe_4N_3O_{16}$
Fw	1658.68	1673.38
T, K	100(2)	100(2)
λ, Å	0.71073	0.71073
Crystal system	Tetragonal	Tetragonal
Space Group	$P\bar{4}2_1m$	$P\bar{4}2_1m$
a, Å	13.8386(3)	13.8167(11)
b, Å	13.8386(3)	13.8167(11)
c, Å	13.9868(3)	13.9832(12)
Cell Volume, $Å^3$	2678.57(13)	2669.4(5)
Z	2	2
D_c, g cm^{-3}	2.057	2.082
μ, mm^{-1}	4.417	4.8241
F(000)	1617	1628
Crystal size, mm	0.18 × 0.16 × 0.13	0.22 × 0.16 × 0.13
θ limits, °	2.070-27.997	2.072-25.499
Reflections collected	46765	28174
R_{int}	0.0496	0.0874
Data / restraints / parameters	3415 / 71 / 203	2608 / 152 / 203
Goodness on fit on F^2	1.098	1.256
R_1 ($I > 2\sigma(I)$)	0.0210	0.0815
wR_2 (all data)	0.0442	0.2040
Absolute structure parameter	0.49(2)	0.44(10)
Largest diff. peak and hole, e $Å^{-3}$	0.496 / -0.479	3.540 / -1.597

Table S29

Crystal data and experimental details for $Cu_3Br_3(dppe)_3$ and $[Cu(dppe)_2]_3[Ag_{13}Fe_8(CO)_{32}]$·solv

	$Cu_3Br_3(dppe)_3$	$[Cu(dppe)_2]_3[Ag_{13}Fe_8(CO)_{32}]$·solv
Formula	$C_{78}H_{72}Br_3Cu_3P_6$	$C_{188}H_{144}Ag_{13}Cu_3Fe_8O_{32}P_{12}$
Fw	1625.52	5323.39
T, K	100(2)	100(2)
λ, Å	0.71073	0.71073
Crystal system	Monoclinic	Monoclinic
Space Group	$P2_1/c$	$C2/c$
a, Å	24.822(5)	28.3874(13)
b, Å	16.776(4)	26.2708(13)
c, Å	18.518(4)	25.5460(12)
β, °	109.629(5)	93.5040(10)
Cell Volume, $Å^3$	7263(3)	19015.6(16)
Z	4	4
D_c, g cm^{-3}	1.487	1.861
μ, mm^{-1}	2.695	2.389
F(000)	3288	10456
Crystal size, mm	0.18 × 0.16 × 0.14	0.22 × 0.20 × 0.16
θ limits, °	1.494-26.000	1.057-25.999
Reflections collected	75872	123534
R_{int}	0.1435	0.0515
Data / restraints / parameters	14069 / 588 / 811	18679 / 2065 / 1400
Goodness on fit on F^2	1.216	1.250
R_1 ($I > 2\sigma(I)$)	0.1339	0.0910
wR_2 (all data)	0.2595	0.1834
Largest diff. peak and hole, e $Å^{-3}$	1.938 / -1.879	4.111 / -2.873
